# Total Synthesis of Mycinamicin IV as Integral Part of a Collective Approach to Macrolide Antibiotics

**DOI:** 10.1002/chem.202104400

**Published:** 2022-01-10

**Authors:** Georg Späth, Alois Fürstner

**Affiliations:** ^1^ Max-Planck-Institut für Kohlenforschung 45470 Mülheim/Ruhr Germany

**Keywords:** antibiotics, collective total synthesis, deoxy sugars, glycosylation, hydroformylation, macrolides

## Abstract

The total synthesis of the 16‐membered macrolide mycinamicin IV is outlined, which complements our previously disclosed, largely catalysis‐based route to the aglycone. This work must also be seen in the context of our recent conquest of aldgamycin N, a related antibiotic featuring a similar core but a distinctly different functionalization pattern. Taken together, these projects prove that the underlying blueprint is integrative and hence qualifies for a collective approach to this prominent class of natural products. In both cases, the final glycosylation phase mandated close attention and was accomplished only after robust de novo syntheses of the (di)deoxy sugars of the desosamine, chalcose, mycinose and aldgarose types had been established. Systematic screening of the glycosidation promoter was also critically important for success.

## Graphical abstract

**Final act**: While a “collective” total synthesis of complex macrolide antibiotics illustrates the power of catalysis, it also shows that the glycosylation of elaborate targets remains far from routine. The success critically hinged on the development of robust new approaches to the peripheral (di)deoxy sugars and the proper choice amongst a set of only seemingly equivalent promoters.

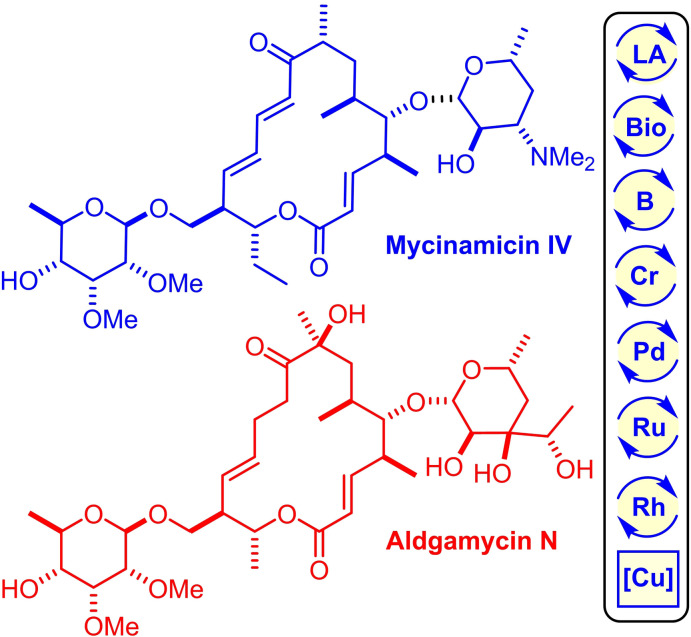

An unmistakable trend in contemporary natural product synthesis is the shift away from the pursuit of individual compounds to the conquest of entire target families.[^1^, ^2^, ^3^] It was within this conceptual framework that we pursued a “collective” synthesis of a class of macrolide antibiotics comprised of several dozen members, for which mycinamicin IV (**1**)[^4^, ^5^] and aldgamycin N (**2**)[^6^, ^7^] are representative (Scheme 1).[^8^, ^9^] Any endeavor chasing this challenging chemical estate must ensure ready access to the common 16‐membered macrolide frame, yet be flexible enough to decorate this core in various ways. In this context, it is pointed out that the carbon framework of **1** is one C‐atom longer than that of **2** (Et‐ versus Me‐ branching off C15) but lacks the hydroxy group at C8 and shows a higher level of unsaturation in the “western” sector. The different glycosylation patterns of these antibiotics made up by rare deoxy sugars present yet another formidable challenge. It seems reasonable to believe that a synthesis blueprint able to encompass these “odd twins” will also bring many additional siblings (and non‐natural analogues thereof) into reach upon deliberate editing of the modules and proper permutation of the assembly process.^[10]^

**Scheme 1:**
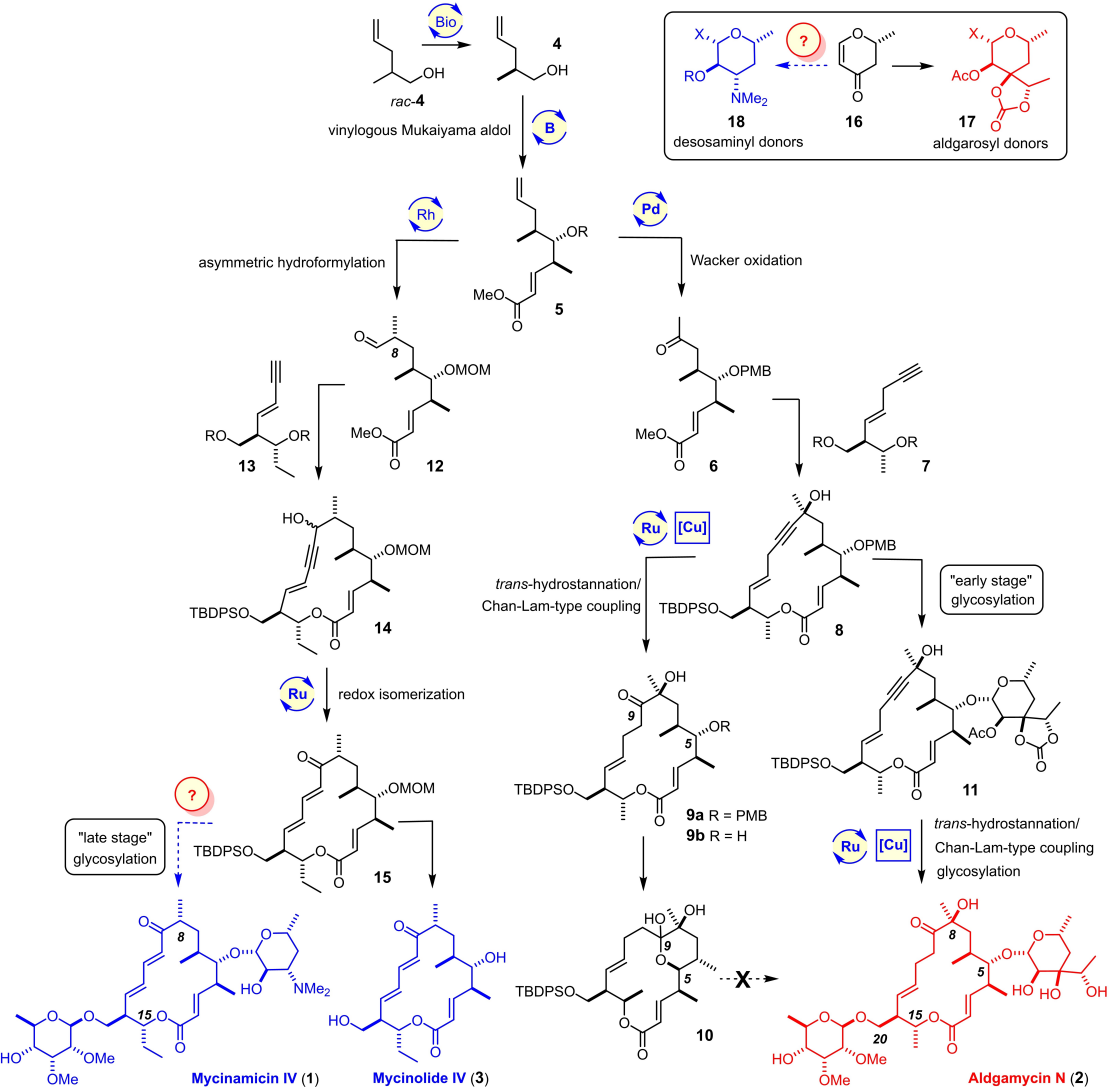
Towards a collective total synthesis of a large family of 16‐membered macrolide antibiotics, represented by the “odd twins” mycinamicin IV (**1**) and aldgamycin N (**2**); MOM=methoxymethyl; PMB=*p*‐methoxybenzyl; TBDPS=*tert*‐butyldiphenylsilyl.

The successful conquest of aldgamycin N (**2**)[^9^, ^11^] as well as the bare macrolide mycinolide IV (**3**),^[8]^ which itself had been a prominent target in the past,[^12^, ^13^, ^14^, ^15^] can be taken as proof‐of‐concept. It relied on the use of compound **5** as the common starting point en route to both product subsets, for which a practical synthesis viable on decagram scale could be established.^[8]^ The alkene terminus of **5** served as the actual branching point, in that it was either subjected to a Tsuji‐Wacker oxidation to give ketone **6** or to a rhodium‐catalyzed asymmetric hydroformylation^[16,17]]^ to produce aldehyde **12**. This latter transformation had been basically without precedent in the context of natural product total synthesis;[^18^, ^19^] it critically hinged upon the use of a MOM‐acetal at the C5‐OH group of **5**, which exerts the proper directing effect and favors formation of the branched product.^[20]^ The subsequent fragment coupling steps took advantage of the fact that the alkyne termini of the “western” segments **7** and **13** are appropriate pre‐nucleophiles. After macrocyclization of the resulting adducts,^[21]^ the stage was set to harness the synthetic equivalence of the triple bonds with the carbonyl groups of the targets through contemporary π‐acid catalysis (**8**→**9**; **14**→**15**).[^22^, ^23^, ^24^, ^25^]

While the assembly of the macrocyclic cores **9** and **15** along this integral blueprint proceeded well, the final glycosylation phase proved far from trivial. Most notable is the fact that all attempts at unveiling the free alcohol **9 b** and reacting it with an appropriate glycosyl donor met with failure because of competing transannular ketalization with irreversible formation of **10**.^[8]^ To remedy this issue, the eponymous aldgarose^[26]^ had to be introduced at an earlier stage (**8**→**11**) and carried through several steps of the longest linear sequence; such a tactic, however, can only be justified if the de novo synthesis of this intricate branched octopyranose is short and efficient. Although this boundary condition was met and the first total synthesis of aldgamycin N (**2**) accomplished,^[9]^ the preparation and proper mounting of the peripheral sugars required considerably more attention than we had anticipated at the outset.

When seen against this backdrop, our parallel conquest of mycinolide IV (**3**) rather than of the fully glycosylated antibiotic mycinamicin IV (**1**) could be seen as missing out on the final challenges.^[8]^ This objection is partly invalidated by the only previous total synthesis of **1**, in which bare **3** had been successfully converted into the target antibiotic.^[12]^ Yet, we still felt the need to complete the project, not least because the literature precedent had explicitly referred to the necessary glycosylations as an “extremely hard problem”.^[12]^ Moreover, the development of a practical alternative access to appropriate D‐desosaminyl donors (**18**) to be attached to the secondary C5‐OH group of the aglycone seemed desirable: acid‐catalyzed degradation of erythromycin produced by fermentation is the currently best source of this valuable dideoxyamino sugar.^[27]^ A remarkably short de novo synthesis of desosamine is also known but proved troublesome in our hands.[^28^, ^29^] Therefore, an entirely new approach was conceived, in which we attempted to derive adequate donors **18** from the exact same building block **16** ^[9]^ that had previously served us very well en route to aldgarose (**17**) (see the Insert in Scheme 1). If successful, this strategy concurs very well with the original plan of a collective approach that requires just a few well‐chosen building blocks to reach an ensemble of diverse and elaborate targets.

As previously described, the asymmetric hetero‐Diels‐Alder reaction of **20** with acetaldehyde catalyzed by the chiral chromium complex **32** ^[30]^ provides multigram amounts of pyranone **16** with an ee of 93 % after acid catalyzed hydrolysis of the crude cycloadduct to facilitate isolation (Scheme 2).^[31]^ Treatment with H_2_O_2_/aq. NaOH in MeOH allowed the equatorially oriented 2‐OH group and methyl glycoside to be set with impeccable selectivity. Although the resulting ketol **21** is in equilibrium with the corresponding dimer **22**,^[9]^ the material could be transformed into the corresponding oxime **25** without incident. Very much to our dismay, however, attempted stereoselective reduction with a variety of metal hydride reagents largely met with failure. Hydrogenation/reductive amination over Pd(OH)_2_/C was to no avail either as it furnished an inseparable mixture of the 2,3‐*cis* configured amino‐alcohol **26** and the desired 2,3‐*trans* configured desosamine derivative **18 b**. We can only speculate about the cause for the surprising epimerization at C2 leading to the formation of **26**, but intervention of a transient enamine/enol form **A** provides a reasonable explanation.

**Scheme 2:**
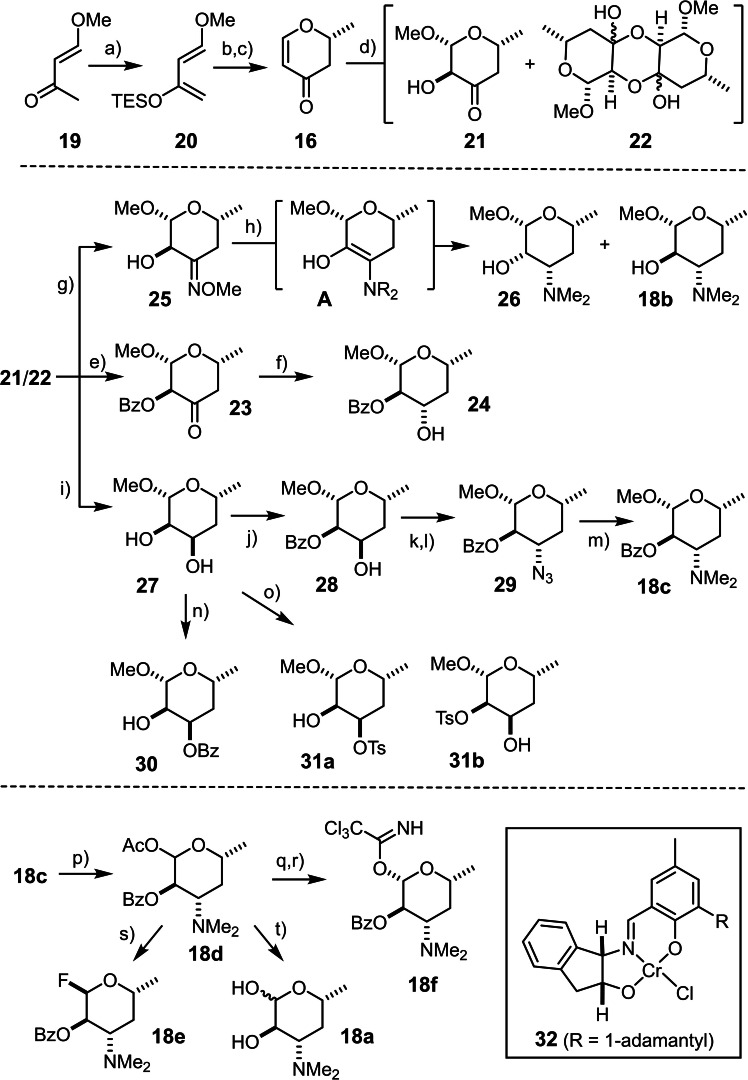
a) TESOTf, Et_3_N, Et_2_O, −20 °C, 92 %; b) **32** (1.5 mol %), MeCHO (neat), −20 °C→RT; c) TFA, CH_2_Cl_2_, 61 % (93 % ee); d) H_2_O_2_, MeOH, aq. NaOH, −45 °C; e) benzoic acid anhydride, pyridine, DMAP cat., CH_2_Cl_2_, 98 % (from **21**/**22**); f) L‐Selectride, THF, −78 °C, 75 %; g) H_2_NOMe⋅HCl, pyridine, MeOH, 88 % (from **21**/**22**); h) H_2_, Pd(OH)_2_/C cat., MeOH, HOAc, then aq. H_2_CO, (dr≈3 : 1); i) Dibal‐H, THF/toluene, 58 % (over both steps); j) benzoyl chloride, DMAP cat., pyridine, CH_2_Cl_2_, 84 %; k) methanesulfonyl chloride, DMAP, CH_2_Cl_2_, quant.; l) NaN_3_, DMF, 90 °C, 82 %; m) H_2_, Pd(OH)_2_/C cat., MeOH, EtOAc, then aq. H_2_CO, 98 %; n) (i) Bu_2_SnO, toluene, reflux; (ii) benzoyl chloride, RT, 80 %; o) (i) Bu_2_SnO, toluene, reflux; (ii) tosyl chloride, DMF, RT, 55 % (**31 a**,+37 % of **31 b**); p) Ac_2_O, H_2_SO_4_, 87 %; q) (i) NH_3_, MeOH, THF, 0 °C, 85 % (from **18 c**); r) Cl_3_CCN, DBU, CH_2_Cl_2_, 80 %; s) HF⋅pyridine, CH_2_Cl_2_, 0 °C, 67 %; t) K_2_CO_3_, MeOH, 96 %; DBU=1,8‐diazabicyclo[5.4.0]undec‐7‐ene; Dibal‐H=diisobutylaluminum hydride; DMAP=4‐dimethylamino‐pyridine; L‐Selectride=lithium tri‐*sec*‐butyl(hydrido)borate; TES=triethylsilyl; Tf=trifluoromethanesulfonyl; TFA=trifluoroacetic acid.

Yet another peculiarity was observed when the derived benzoate **23** was reacted with L‐selectride in THF, which gave the corresponding *trans*‐configured diol derivative **24** as the only product. Puzzled by this again unforeseen course, the mixture of **21**/**22** was directly reduced with Dibal‐H; as this reaction led to the opposite stereochemical outcome, the selective formation of **24** is tentatively ascribed to an intervention of the adjacent benzoate in **23**. As expected, treatment of diol **27** with one equivalent of benzoyl chloride and catalytic DMAP in CH_2_Cl_2_/pyridine resulted in selective acylation of the equatorial ‐OH group to give **28** in good yield, whereas benzoylation of a transient stannylene acetal^[32]^ furnished the regioisomeric ester **30** exclusively. The mesylate derived from **28** reacted with NaN_3_ in DMF at elevated temperature to provide **29**, which was transformed into the desired dimethylamine derivative **18 c** by hydrogenolysis/reductive amination in a one‐pot operation. This route proved much more efficient than the conceivable alternative sequence commencing with tosylation of the axial −OH group of **27** followed by conversion of **31 a** into **29**, because the sulfonylation reaction was only modestly selective.

Compound **18 c** was elaborated into presumably adequate glycosyl donors by cleavage of the methyl glycoside under acylating conditions; exposure of the resulting anomeric acetate **18 d** to HF⋅pyridine gave glycosyl fluoride **18 e**.^[33]^ Alternatively, aminolysis of **18 d** paved the way to the corresponding trichloroacetimidate **18 f**.^[34]^ Of course, the parent D‐desosamine (**18 a**) itself is also readily available from **18 d** upon concomitant cleavage of both esters with K_2_CO_3_ in MeOH. This sugar is not only present in the mycinamicin series discussed herein, but in a large number of iconic macrolide antibiotics and innumerous semisynthetic derivatives thereof (Figure 1).[^35^, ^36^, ^37^] Although the current synthesis is longer than the shortest known access routes to desosaminyl donors,^[38]^ we found it significantly more robust, practical, high yielding, and flexible:^[39]^ if desirable, one could easily divert it towards the preparation of various regio‐ and stereomers of desosamine. Moreover, some intermediates themselves (or simple derivatives thereof) are part of other natural products, ranging from the bespoken macrolide antibiotics to various steroidal glycosides (Figure 1). Since asymmetric catalysis is the gatekeeper, it is equally facile to obtain the enantiomers of all of these valuable sugars; because some of them actually appear in nature (for example, **27**/*ent*‐**27**), this aspect is also truly relevant.

**Figure 1:**
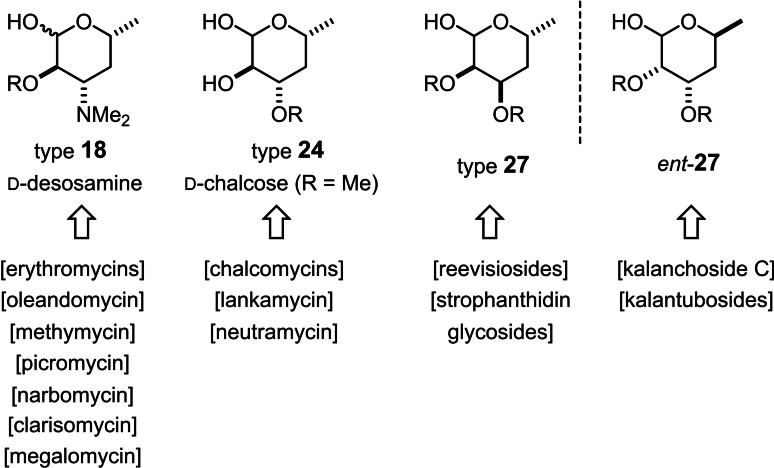
The pedigree of selected 4,6‐dideoxy sugars.

With ample quantities of appropriate glycosyl donors in hand, the current project entered into the critical glycosylation phase. The course of action emanates from the protecting group pattern of aglycone **15** in that introduction of desosamine at the secondary C5‐OH should *precede* the attachment of mycinose at the primary C21‐OH position; if carried out in the reverse order, the MOM‐acetal would need to be deprotected with a (Lewis) acid in the presence of a pre‐existing glycosidic bond, which might jeopardize success. As mentioned above, the choice of these particular protecting groups reflects inherent constraints encountered during the branch‐selective asymmetric hydroformylation of the key building block **5**.[^8^, ^20^]

When seen against this backdrop, the end game had to start with the selective removal of the MOM‐acetal. Unexpectedly, treatment of **15** with aqueous HCl in MeOH at ambient temperature cleaved the TBDPS‐ether faster than the MOM‐group to give the primary alcohol derivative **33** as the major product (Scheme 3). The use of Me_2_BBr followed by work‐up of the resulting mixture with aq. Na_2_CO_3_ in THF remedied the issue and furnished compound **34** in high yield.[^40^, ^41^] Attempted introduction of the desosamine residue to the liberated site in analogy to the only previous total synthesis of mycinamicin IV was met with poor results. In this literature precedent, an almost identical substrate had been reacted with great success with the glycosyl fluoride **18 e** in the presence of Cp_2_HfCl_2_/AgClO_4_ as fluorophilic activating agent (β : α=6 : 1, 72 %).[^12^, ^42^] Unfortunately, we have neither been able to reach a similarly good anomer ratio nor has the yield been anywhere close. Although a full optimization was not undertaken, none of our orienting trials was overly promising. Therefore we were prompted to explore the use of the trichloroacetimidate **18 f**, not least because this methodology^[34]^ had proven superior to the use of glycosyl fluoride donors in our total synthesis of aldgamycin N (**2**).^[9]^ In line with this prior experience, treatment of **34** with excess **18 f** in the presence of TBSOTf furnished the desired β‐glycoside **35** as the only anomer in 84 % yield after cleavage of the terminal silyl ether with TBAF to facilitate purification of the product. It is important to note that this excellent outcome mandated the use of TBS‐OTf as promoter; the otherwise more commonly used TMS‐OTf preferentially activated the ketone of **34** and entailed transannular cyclization with formation of enol ether **36** when the reaction was performed at or below 0 °C; at ambient temperature, TMS‐OTf as well as TES‐OTf simply led to silylation of the secondary C5‐OH group to give **37**. These observations provide a striking illustration for the arguably underappreciated fact that systematic screening of the silyl triflate promotor is worth the effort as it can obviously exert a dramatic effect and thus decide on success or failure of a projected glycosylation in a challenging setting.^[43]^

**Scheme 3:**
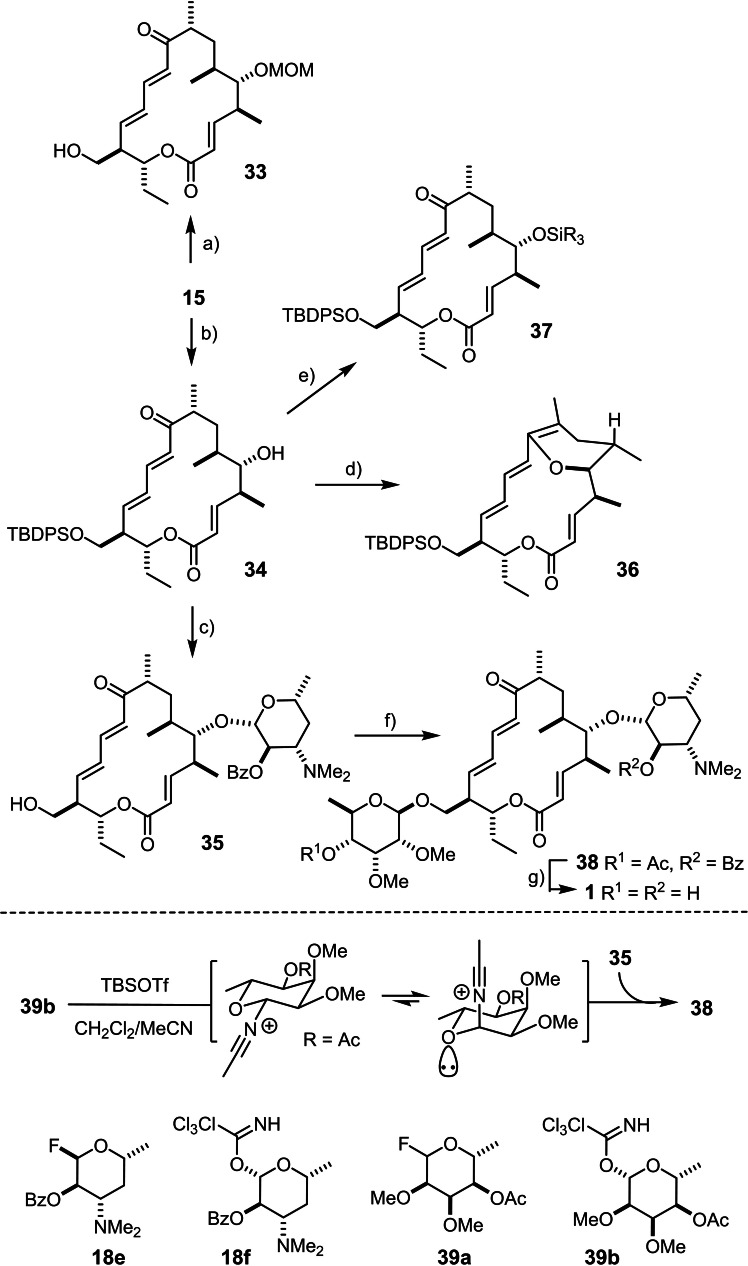
a) aq. HCl, MeOH, 52 %; b) Me_2_BBr, CH_2_Cl_2_, −78 °C, then aq. Na_2_CO_3_, THF, RT, 80 %; c) (i) **18 f**, TBSOTf, CH_2_Cl_2_; (ii) TBAF, THF, 84 %; d) **18 f**, TMSOTf, CH_2_Cl_2_, −30 °C→RT, 30 %; e) **18 f**, TMSOTf, CH_2_Cl_2_, RT, 44 % (R=Me); f) **39 b**, TBSOTf, CH_2_Cl_2_/MeCN (1 : 1), 33 %; g) Et_3_N, MeOH, H_2_O, 70 °C, 71 %; TBAF=tetra‐*n*‐butylammonium fluoride.

Similar problems were faced in the final introduction of the yet missing mycinose at the primary −OH terminus when resorting to the fluoride donor **39 a**.^[9]^ Although the literature reports an almost exclusive and high yielding formation of the desired β‐anomer (α : β=1 : 26, 86 %),^[12]^ it was the α‐anomer that was slightly favored in our hands for reasons that are not entirely clear, even though we tried to follow the reported conditions as closely as possible.^[44]^ In the end, we again resorted to the use of the trichloroacetimidate **39 b** in combination with TBSOTf, which gave the β‐glycoside exclusively, albeit in modest yield, when the reaction was performed under high dilution conditions in CH_2_Cl_2_/MeCN.^[45]^ Since the glycosyl donor carries a “non‐participating” methyl ether at the C2‐position, this remarkable selectivity has to be ascribed to the intervention of MeCN that is thought to coordinate to the transient oxocarbenium intermediate, preferentially in axial orientation for stereoelectronic reasons (“nitrile effect”).[^46^, ^47^] The final cleavage of the two different acyl groups at the two sugar residues of product **38** thus formed was accomplished with Et_3_N in MeOH/H_2_O,^[12]^ whereas aq. LiOH or Ba(OH)_2_ in THF saponified only the acetate even when the reaction was carried out at 50 °C overnight. The analytical and spectral data of synthetic mycinamicin IV (**1**) were in excellent agreement with those of the natural product previously reported in the literature (see the Supporting Information).

When seen from close up, the second conquest of the emblematic macrolide antibiotic mycinamicin IV (**1**) completed herein may be taken as an illustration for the methodological advances in organic chemistry since the time when the first total synthesis of this demanding target was disclosed. Not only is the new route considerably shorter than its ancestor (16 versus 32 steps, longest linear sequence),^[12]^ but it is also largely catalysis‐based rather than relying on the “chiral pool”. As such, it features the first branch‐selective asymmetric and fully catalyst‐controlled hydroformylation of an ordinary terminal alkene substrate in the context of total synthesis, an advanced application of a ruthenium catalyzed redox isomerization, as well as a rare example of direct transesterification for the closure of a macrolactone ring.^[8]^ In parallel, robust yet flexible new approaches to the peripheral (di)deoxysugars of the desosamine, chalcose, aldgamycin,^[9]^ and mycinose^[9]^ type were developed, which are enabling in other context too since these sugars are prominently featured in a considerable number of bioactive natural products of, in part, different chemotypes.

When assessed at the meta‐level, the current total synthesis of mycinamcin IV complements our previous work on aldgamycin N;^[9]^ because these two targets are representative for a large number of antibiotics and because the underlying blueprint is integrative and modular, a solid foundation for a collective synthesis of this important class of natural products and their analogues is laid out. Further work in our laboratory intends to take advantage of this notion.

## Conflict of interest

The authors declare no conflict of interest.

## Supporting information

As a service to our authors and readers, this journal provides supporting information supplied by the authors. Such materials are peer reviewed and may be re‐organized for online delivery, but are not copy‐edited or typeset. Technical support issues arising from supporting information (other than missing files) should be addressed to the authors.

Supporting Information

## Data Availability

Research data are not shared.

## References

[1a] L. Li , Z. Chen , X. Zhang , Y. Jia , Chem. Rev. 2018, 118, 3752–3832; 29516724 10.1021/acs.chemrev.7b00653

[1b] K. E. Kim , A. N. Kim , C. J. McCormick , B. M. Stoltz , J. Am. Chem. Soc. 2021, 143, 16890–16901. 34614361 10.1021/jacs.1c08920 PMC9285880

[2] For leading references, see the following and literature cited therein:

[2a] A. Hirose , A. Watanabe , K. Ogino , M. Nagatomo , M. Inoue , J. Am. Chem. Soc. 2021, 143, 12387–12396; 34319739 10.1021/jacs.1c06450

[2b] J. H. Kim , H. Jeon , C. Park , S. Park , S. Kim , Angew. Chem. Int. Ed. 2021, 60, 12060–12065; 10.1002/anie.202101766 33733565

[2c] W. Liu , R. Patouret , S. Barluenga , M. Plank , R. Loewith , N. Winssinger , ACS Cent. Sci. 2021, 7, 954–962; 34235256 10.1021/acscentsci.1c00056 PMC8227592

[2d] M. J. Anketell , T. M. Sharrock , I. Paterson , Angew. Chem. Int. Ed. 2020, 59, 1572–1576; 10.1002/anie.201914042 31743574

[2e] S.-H. Wang , R.-Q. Si , Q.-B. Zhuang , X. Guo , T. Ke , X.-M. Zhang , F.-M. Zhang , Y.-Q. Tu , Angew. Chem. Int. Ed. 2020, 59, 21954–21958; 10.1002/anie.202009238 32851781

[2f] Y. Zou , X. Li , Y. Yang , S. Berritt , J. Melvin , S. Gonzales , M. Spafford , A. B. Smith , J. Am. Chem. Soc. 2018, 140, 9502–9511; 30028603 10.1021/jacs.8b04053 PMC6085755

[2g] C. R. Jamison , J. J. Badillo , J. M. Lipshutz , R. J. Comito , D. W. C. MacMillan , Nat. Chem. 2017, 9, 1165; 29168485 10.1038/nchem.2825 PMC9815142

[2h] H. Cheng , Z. Zhang , H. Yao , W. Zhang , J. Yu , R. Tong , Angew. Chem. Int. Ed. 2017, 56, 9096–9100; 10.1002/anie.201704628 28574668

[2i] W. Zi , Z. Zuo , D. Ma , Acc. Chem. Res. 2015, 48, 702–711; 25667972 10.1021/ar5004303

[2j] O. Wagnières , Z. Xu , Q. Wang , J. Zhu , J. Am. Chem. Soc. 2014, 136, 15102–15108; 25270053 10.1021/ja509329x

[2k] Ren , Y. Bian , Z. Zhang , H. Shang , P. Zhang , Y. Chen , Z. Yang , T. Luo , Y. Tang , Angew. Chem. Int. Ed. 2012, 51, 6984–6988; 10.1002/anie.201202643 22674838

[2l] S. B. Jones , B. Simmons , A. Mastracchio , D. W. C. MacMillan , Nature 2011, 475, 183–188. 21753848 10.1038/nature10232 PMC3439143

[3] For examples from this laboratory, see:

[3a] L. E. Löffler , C. Wirtz , A. Fürstner , Angew. Chem. Int. Ed. 2021, 60, 5316–5322; 10.1002/anie.202015243 PMC7986786 33289954

[3b] S. Schulthoff , J. Y. Hamilton , M. Heinrich , Y. Kwon , C. Wirtz , A. Fürstner , Angew. Chem. Int. Ed. 2021, 60, 446–454; 10.1002/anie.202011472 PMC7821135 32946141

[3c] Z. Meng , S. M. Spohr , S. Tobegen , C. Farès , A. Fürstner , J. Am. Chem. Soc. 2021, 143, 14402–14414; 34448391 10.1021/jacs.1c07955 PMC8431342

[3d] M. Heinrich , J. J. Murphy , M. K. Ilg , A. Letort , J. T. Flasz , P. Philipps , A. Fürstner , J. Am. Chem. Soc. 2020, 142, 6409–6422; 32142305 10.1021/jacs.0c01700 PMC7307910

[3e] J. Willwacher , B. Heggen , C. Wirtz , W. Thiel , A. Fürstner , Chem. Eur. J. 2015, 21, 11387–11392; 26094957 10.1002/chem.201501491

[3f] A. Larivée , J. B. Unger , M. Thomas , C. Wirtz , C. Dubost , S. Handa , A. Fürstner , Angew. Chem. Int. Ed. 2011, 50, 304–309; 10.1002/anie.201005850 21082641

[3g] K. Micoine , P. Persich , J. Llaveria , M.-H. Lam , A. Maderna , F. Loganzo , A. Fürstner , Chem. Eur. J. 2013, 19, 7370–7383; 23595541 10.1002/chem.201300393

[3h] J. Gagnepain , E. Moulin , C. Nevado , M. Waser , A. Maier , G. Kelter , H.-H. Fiebig , A. Fürstner , Chem. Eur. J. 2011, 17, 6973–6984; 21557355 10.1002/chem.201100180

[3i] A. Fürstner , L. C. Bouchez , L. Morency , J.-A. Funel , V. Liepins , F.-H. Porée , R. Gilmour , D. Laurich , F. Beaufils , M. Tamiya , Chem. Eur. J. 2009, 15, 3983–4010; 19241433 10.1002/chem.200802067

[3j] A. Fürstner , D. De Souza , L. Turet , M. D. B. Fenster , L. Parra-Rapado , C. Wirtz , R. Mynott , C. W. Lehmann , Chem. Eur. J. 2007, 13, 115–134; 17091520 10.1002/chem.200601135

[3k] A. Fürstner , J. W. J. Kennedy , Chem. Eur. J. 2006, 12, 7398–7410; 16881031 10.1002/chem.200600592

[3l] A. Fürstner , M. M. Domostoj , B. Scheiper , J. Am. Chem. Soc. 2006, 128, 8087–8094. 16771525 10.1021/ja0617800

[4a] M. Hayashi , M. Ohno , K. Kinoshita , S. Satoi , M. Suzuki , K. Harada , J. Antibiot. 1981, 34, 346–349; 10.7164/antibiotics.34.346 7275814

[4b] M. Hayashi , H. Ohara , M. Ohno , H. Sakakibara , S. Satoi , K. Harada , M. Suzuki , J. Antibiot. 1981, 34, 1075–1077; 10.7164/antibiotics.34.1075 7332707

[4c] S. Satoi , N. Muto , M. Hayashi , T. Fuji , M. Otani , J. Antibiot. 1980, 33, 364–376. 10.7164/antibiotics.33.364 7410205

[5] K. Kinoshita , S. Satoi , M. Hayashi , K. Nakatsu , J. Antibiot. 1989, 42, 1003–1005. 10.7164/antibiotics.42.1003 2737943

[6] C.-X. Wang , R. Ding , S.-T. Jiang , J.-S. Tang , D. Hu , G.-D. Chen , F. Lin , K. Hong , X.-S. Yao , H. Gao , J. Nat. Prod. 2016, 79, 2446–2454. 27690254 10.1021/acs.jnatprod.6b00200

[7] For other aldgamycins, see:

[7a] M. P. Kunstmann , L. A. Mitscher , E. L. Patterson , Antimicrob. Agents Chemother. 1964, 10, 87–90; 14288037

[7b] G. A. Ellestad , M. P. Kunstmann , J. E. Lancaster , L. A. Mitscher , G. Morton , Tetrahedron 1967, 23, 3893–3902; 6065624 10.1016/s0040-4020(01)97899-8

[7c] H. Achenbach , W. Karl , Chem. Ber. 1975, 108, 780–789;

[7d] S. Mizobuchi , J. Mochizuki , H. Soga , H. Tanba , H. Inoue , J. Antibiot. 1986, 39, 1776–1778; 10.7164/antibiotics.39.1776 3818451

[7e] J.-S. Park , H. O. Yang , H. C. Kwon , J. Antibiot. 2009, 62, 171–175. 10.1038/ja.2009.6 19218982

[8] B. Herlé , G. Späth , L. Schreyer , A. Fürstner , Angew. Chem. Int. Ed. 2021, 60, 7893–7899; 10.1002/anie.202016475 PMC8048839 33448619

[9] G. Späth , A. Fürstner , Angew. Chem. Int. Ed. 2021, 60, 7900–7905; 10.1002/anie.202016477 PMC8048874 33448589

[10] For the arguably most convincing case of a modular synthesis of (mostly non-natural) macrolide antibiotics known to date, see: I. B. Seiple , Z. Zhang , P. Jakubec , A. Langlois Mercier , P. M. Wright , D. T. Hog , K. Yabu , S. Allu , T. Fukuzaki , P. Carlsen , Y. Kitamura , X. Zhou , M. L. Gondakes , F. T. Szczypinski , W. D. Green , A. G. Myers , Nature 2016, 513, 338–345. 10.1038/nature17967 PMC6526944 27193679

[11] For a study towards aldgamycin M, see: K. Muralikrishna , V. Satyanarayana , G. C. Kumar , J. S. Yadav , ChemistrySelect 2019, 4, 3002–3005.

[12] For the only previous total synthesis of mycinamicin IV and VII, see:

[12a] K. Suzuki , T. Matsumoto , K. Tomooka , K. Matsumoto , G. Tsuchihashi , Chem. Lett. 1987, 16, 113–116;

[12b] T. Matsumoto , H. Maeta , K. Suzuki , G. Tsuchihashi , Tetrahedron Lett. 1988, 29, 3575–3578.

[13] Protomycinolide IV:

[13a] M. Honda , T. Katsuki , M. Yamaguchi , Tetrahedron Lett. 1984, 25, 3857–3860;

[13b] K. Suzuki , K. Tomooka , E. Katayama , T. Matsumoto , G. Tsuchihashi , J. Am. Chem. Soc. 1986, 108, 5221–5229.

[14] K. Ditrich , T. Bube , R. Stürmer , R. W. Hoffmann , Angew. Chem. Int. Ed. Engl. 1986, 25, 1028–1030;

[15] For the synthesis of various fragments, see:

[15a] K. Ditrich , R. W. Hoffmann , Liebigs Ann. Chem. 1990, 15–21;

[15b] K. Tomooka , K. Matsumoto , K. Suzuki , G. Tsuchihashi , Synlett 1992, 129–130;

[15c] Y. Sekiguchi , K. Ogasawara , S. Takano , Heterocycles 1992, 33, 743–755;

[15d] Y. Ogawa , K. Kuroda , T. Mukaiyama , Chem. Lett. 2005, 34, 698–699;

[15e] F. K. Meng , F. Haeffner , A. H. Hoveyda , J. Am. Chem. Soc. 2014, 136, 11304–11307. 25089917 10.1021/ja5071202 PMC4140502

[16a] G. M. Noonan , J. A. Fuentes , C. J. Cobley , M. L. Clarke , Angew. Chem. Int. Ed. 2012, 51, 2477–2480; 10.1002/anie.201108203 22287282

[16b] P. Dingwall , J. A. Fuentes , L. Crawford , A. M. Z. Slawin , M. Bühl , M. L. Clarke , J. Am. Chem. Soc. 2017, 139, 15921–15932; 29068679 10.1021/jacs.7b09164

[16c] L. Iu , J. A. Fuentes , M. E. Janka , K. J. Fontenot , M. L. Clarke , Angew. Chem. Int. Ed. 2019, 58, 2120–2124; 10.1002/anie.201811888 30561885

[17a] J. R. Coombs , J. P. Morken , Angew. Chem. Int. Ed. 2016, 55, 2636–2649; 10.1002/anie.201507151 PMC4913282 26764019

[17b] G. W. Wong , C. R. Landis , Aldrichimica Acta 2014, 47, 29–38.

[18] A total synthesis of ambruticin features the arguably most advanced example of an asymmetric hydroformylation; in this case, however, a 1,3-diene was used, which has an inherently higher bias for branch-selectivity than an ordinary terminal alkene because the reaction passes through a favorable allylrhodium intermediate:

[18a] P. Liu , E. N. Jacobsen , J. Am. Chem. Soc. 2001, 123, 10772–10773; for a related branch-selective hydroformylation of a 1,3-diene in a study toward tedanolide C, see:11674024 10.1021/ja016893s

[18b] T. E. Smith , S. J. Fink , Z. G. Levine , K. A. McClelland , A. A. Zackheim , M. E. Daub , Org. Lett. 2012, 14, 1452–1455. 22375885 10.1021/ol300194x PMC3312041

[19] Asymmetric hydroformylation of simple acrolein acetals and acrylic acid orthoesters were used to prepare some building blocks for the syntheses of dictyostatin and the Prelog-Djerassi lactone, see:

[19a] S. Ho , C. Bucher , J. L. Leighton , Angew. Chem. Int. Ed. 2013, 52, 6757–6761; 10.1002/anie.201302565 PMC3812691 23666786

[19b] R. M. Risi , S. D. Burke , Org. Lett. 2012, 14, 2572–2575. 22559226 10.1021/ol3008765

[20] The free alcohol **5** (R=H) also gave appreciable amounts of the desired branched aldehyde, which exists exclusively in lactol from. Further attempts at optimizing this promising result were eventually discontinued when we noticed that this lactol failed to react with alkynylmetal reagents derived from enyne **13** under a variety of conditions and a detour would have been necessary to overcome this impasse. More elaborate auxiliaries covalently attached to the hydroxy group known to favor the branched product (Ph_2_PO−, *o*-(Ph_2_P)C_6_H_4_COO−) proved impractical and partly unstable and were hence disregarded. Amongst the “ordinary” protecting groups, the MOM-acetal gave by far the best results.

[21] A. Fürstner , Acc. Chem. Res. 2021, 54, 861–874. 33507727 10.1021/acs.accounts.0c00759 PMC7893715

[22] A. Fürstner , J. Am. Chem. Soc. 2019, 141, 11–24. 30422659 10.1021/jacs.8b09782

[23] H. Sommer , J. Y. Hamilton , A. Fürstner , Angew. Chem. Int. Ed. 2017, 56, 6161–6165; 10.1002/anie.201701391 28436199

[24a] S. M. Rummelt , K. Radkowski , D.-A. Rosca , A. Fürstner , J. Am. Chem. Soc. 2015, 137, 5506–5519; 25822126 10.1021/jacs.5b01475

[24b] D.-A. Rosca , K. Radkowski , L. M. Wolf , M. Wagh , R. Goddard , W. Thiel , A. Fürstner , J. Am. Chem. Soc. 2017, 139, 2443–2455. 28169542 10.1021/jacs.6b12517

[25] S. Schaubach , K. Gebauer , F. Ungeheuer , L. Hoffmeister , M. K. Ilg , C. Wirtz , A. Fürstner , Chem. Eur. J. 2016, 22, 8494–8507. 27203803 10.1002/chem.201601163

[26] M. P. Kunstmann , L. A. Mitscher , N. Bohonos , Tetrahedron Lett. 1966, 7, 839–846.

[27] P. C. Hogan , C.-L. Chen , K. M. Mulvihill , J. E. Lawrence , E. Moorhead , J. Rickmeier , A. G. Myers , J. Antibiot. 2018, 71, 318–325. 10.1038/ja.2017.116 29018266

[28] Z. Zhang , T. Fukuzaki , A. G. Myers , Angew. Chem. Int. Ed. 2016, 55, 523–527; 10.1002/anie.201507357 26612347

[29] For yet another short route starting from d-glucose and for a survey of earlier syntheses, see: V. Velvadapu , R. B. Andrade , Carbohydr. Res. 2008, 343, 145–150. 17977522 10.1016/j.carres.2007.10.004

[30] A. G. Dossetter , T. F. Jamison , E. N. Jacobsen , Angew. Chem. Int. Ed. 1999, 38, 2398–2400; 10.1002/(sici)1521-3773(19990816)38:16<2398::aid-anie2398>3.0.co;2-e 10458800

[31] For *rac*-**16**, see: J. F. Kerwin , S. J. Danishefsky , J. Org. Chem. 1982, 47, 1597–1598.

[32] S. David , S. Hanessian , Tetrahedron 1985, 41, 643–663.

[33] K. C. Nicolaou , H. Ueno , in Preparative Carbohydrate Chemistry (S. Hanessian, Ed.), Dekker, New York, 1997, pp. 313–338.

[34a] R. R. Schmidt , Angew. Chem. Int. Ed. Engl. 1986, 25, 212–235;

[34b] R. R. Schmidt , K.-H. Jung , in Preparative Carbohydrate Chemistry (S. Hanessian, Ed.), Dekker, New York, 1997, pp. 283–312.

[35] S. Omura , Macrolide Antibiotics. Chemistry, Biology and Practice, 2^nd^ Ed, Academic Press, 2002.

[36] F. v. Nussbaum , M. Brands , B. Hinzen , S. Weigand , D. Häbich , Angew. Chem. Int. Ed. 2006, 45, 5072–5129; 10.1002/anie.200600350 16881035

[37] S. I. Elshahawi , K. A. Shaaban , M. K. Kharel , J. S. Thorson , Chem. Soc. Rev. 2015, 44, 7591–7697. 25735878 10.1039/c4cs00426d PMC4560691

[38] Degradation of erythromycin followed by two chemical steps is the shortest route, cf. Ref. [27]; the shortest known purely chemical approach comprises four steps to free desosamine followed by two steps for its functionalization, cf. [28, 29].

[39] Such flexibility is highly desirable in the medicinal chemistry context; for a recent case study targeting a panel of isomeric 4,6-dideoxyhexoses by degradation of streptomycin, see: Y. Zhang , J. Zhang , L. V. Ponomareva , Z. Cui , S. G. van Lanen , J. S. Thorson , J. Am. Chem. Soc. 2020, 142, 9389–9395. 32330028 10.1021/jacs.9b13766 PMC7453097

[40] Y. Guidon , C. Yoakim , H. E. Morton , J. Org. Chem. 1984, 49, 3912–3920.

[41] For use in total synthesis from this laboratory, see Ref. [3d] and the following:

[41a] D. Mailhol , J. Willwacher , N. Kausch-Busies , E. E. Rubitski , Z. Sobol , M. Schuler , M.-H. Lam , S. Musto , F. Loganzo , A. Maderna , A. Fürstner , J. Am. Chem. Soc. 2014, 136, 15719–15729; 25347620 10.1021/ja508846g

[41b] K. Lehr , S. Schulthoff , Y. Ueda , R. Mariz , L. Leseurre , B. Gabor , A. Fürstner , Chem. Eur. J. 2015, 21, 219–227. 25367701 10.1002/chem.201404873

[42] For the development of this promoter system, see: K. Suzuki , H. Maeta , T. Matsumoto , G.-I. Tsuchihashi , Tetrahedron Lett. 1988, 29, 3571–3574.

[43] A similarly dramatic example is contained in our total synthesis of aldgamycin, cf. Ref. [9]; for yet another case, see: W. R. Roush , S. Narayan , Org. Lett. 1999, 1, 899–902.10823220

[44] The stereochemical outcome was independent of whether the α- or the β-anomer of glycosyl fluoride **39 a** was used.

[45] For precedent showing that high dilution can favor β-glycoside formation with glycosyl donors devoid of “participating” 2-acyl substituents, see:

[45a] C.-S. Chao , C.-W. Li , M.-C. Chen , S.-S. Chang , K.-K. T. Mong , Chem. Eur. J. 2009, 15, 10972–10982; 19746471 10.1002/chem.200901119

[45b] C.-S. Chao , C.-Y. Lin , S. Mulani , W.-C. Hung , K.-K. T. Mong , Chem. Eur. J. 2011, 17, 12193–12202. 21915924 10.1002/chem.201100732

[46] R. R. Schmidt , M. Behrendt , A. Toepfer , Synlett 1990, 694–696.

[47] For previous exploitation of this effect in total synthesis by our laboratory, see:

[47a] A. Fürstner , J. Mlynarski , M. Albert , J. Am. Chem. Soc. 2002, 124, 10274–10275; 12197718 10.1021/ja027346p

[47b] A. Fürstner , M. Albert , J. Mlynarski , M. Matheu , E. DeClercq , J. Am. Chem. Soc. 2003, 125, 13132–13142; 14570487 10.1021/ja036521e

[47c] A. Fürstner , J. Ruiz-Caro , H. Prinz , H. Waldmann , J. Org. Chem. 2004, 69, 459–467; 14725460 10.1021/jo035079f

[47d] J. Mlynaski , J. Ruiz-Caro , A. Fürstner , Chem. Eur. J. 2004, 10, 2214–2222. 15112210 10.1002/chem.200305588

