# Supplementary material for: Total Synthesis of Mycinamicin IV as Integral Part of a Collective Approach to Macrolide Antibiotics
Source: Chemistry. 2022 Jan 10;28(11):e202104400. doi: 10.1002/chem.202104400 (PMC9305142; doi:10.1002/chem.202104400)
Supplement: Supplementary file 1 — Supporting Information [file CHEM-28-0-s001.pdf]

Chemistry–A European Journal

Supporting Information

Total Synthesis of Mycinamicin IV as Integral Part of a Collective Approach to Macrolide Antibiotics

Georg Späth and Alois Fürstner*

General

Unless otherwise stated, all reactions were performed in oven-dried (80 °C) or flame-dried glassware in anhydrous solvents under argon, applying standard Schlenk techniques. Dry argon (>99.5%) was purchased from Air Liquide.

The following solvents were purified by distillation over the indicated drying agents and transferred under argon: tetrahydrofuran and diethyl ether (Mg/anthracene), dichloromethane (CaH_2), hexanes and toluene (Na/K), methanol (Mg, stored over 3 Å molecular sieves). Acetonitrile, dimethyl sulfoxide, dimethylformamide, pyridine and triethylamine were dried using an adsorption (molecular sieves) solvent purification system. During work up, solvents were removed under reduced pressure below 40 °C using a rotary evaporator.

Dimethylboron bromide was obtained following a literature procedure and stored as a solution in dichloromethane at −20 °C under an argon atmosphere.[1]

Thin layer chromatography (TLC) was performed on Macherey-Nagel precoated plates (POLYGRAM® SIL/UV254); the compounds were detected by UV light (254 nm) or heating of the plate with a heat gun after treatment with stain solutions comprising either potassium permanganate or phosphomolybdic acid. Flash chromatography was performed with VWR silica gel 60 (40 – 63 µm). Automated column chromatography was conducted on a Biotage® Isolera™ or a Biotage® Selekt instrument, using the chromatography cartridges indicated in the respective procedure. Diastereomeric ratios (d.r.) of intermediates were determined by ^{1}H NMR spectroscopy from the relative integrals of sufficiently separated, characteristic signals of the respective compound.

NMR spectra were recorded on Bruker AV 400, AV 500 or AVIII 600 spectrometers in the solvents indicated. The solvent signals were used as references, chemical shifts were converted to the TMS scale and reported as follows: chemical shift in ppm (multiplicity, coupling constant *J* in Hz, number of protons). Multiplets are designated by the following abbreviations: s for singlet, d for doublet, t for triplet, q for quartet, quint for quintet, m for complex pattern (multiplet); the abbreviation br indicates a broad signal. ^{13}C NMR spectra were recorded in {^{1}H}-decoupled mode. Melting points were determined using a Büchi B-540 apparatus. IR spectra were recorded on a Bruker Alpha Platinum ATR spectrometer at room temperature. Mass spectra were recorded using the following instruments: MS (EI): Finnigan MAT 8200 (70 eV), ESI-MS: Bruker ESQ3000, accurate mass determinations: Bruker APEX III FT-MS (7 T magnet) or Finnigan MAT 95. GC-MS samples were processed on a Shimadzu GCMS-QP2010 Ultrainstrument. Specific optical rotatory power ($[\boldsymbol{\alpha}]_\mathbf{D}$) was measured with an

A-Krüss Otronic Model P8000-t polarimeter at a wavelength of 589 nm. The values are given with respect to exact temperature, concentration (c/(10mg/mL)) and solvent.

Sugar Building Blocks

(2*R*,3*S*,6*R*)-2-Methoxy-6-methyl-4-oxotetrahydro-2*H*-pyran-3-yl benzoate (23). In a 25-mL round-bottom flask, benzoic acid anhydride (90% *w*/*w*, 686 mg, 2.73 mmol) and 4-(dimethylamino)pyridine (47.7 mg, 0.390 mmol) were added to a solution of ketol **21** (288 mg, 1.80 mmol; as a mixture with its dimer **22**) and pyridine (0.36 mL, 4.5 mmol) in dichloromethane (10 mL) at 0 °C (ice bath). The cooling bath was removed, and the reaction mixture was stirred for 6 h at room temperature. The mixture was diluted with dichloromethane (15 mL) and washed with saturated aqueous sodium bicarbonate solution (20 mL). The layers were separated, and the aqueous phase was extracted with dichloromethane (3 × 10 mL). The combined organic layers were dried over anhydrous sodium sulfate, the drying agent was filtered off, and the solvent was removed under reduced pressure. Purification of the residue by flash chromatography (hexanes/EtOAc, 7:1) furnished the title compound as a white solid (466 mg, 98% yield). ^{1}H NMR (400 MHz, $CDCl_3$): δ 8.15 – 8.04 (m, 2H), 7.65 – 7.55 (m, 1H), 7.51 – 7.42 (m, 2H), 5.30 (dd, *J* = 8.1, 1.0 Hz, 1H), 4.72 (d, *J* = 8.1 Hz, 1H), 3.85 (dqd, *J* = 10.5, 6.1, 3.4 Hz, 1H), 3.59 (s, 3H), 2.64 – 2.50 (m, 2H), 1.44 (d, *J* = 6.1 Hz, 3H). ^{13}C{^{1}H} NMR (101 MHz, $CDCl_3$): δ 199.3, 165.4, 133.5, 130.2, 129.4, 128.5, 102.6, 78.5, 68.4, 57.2, 48.6, 21.4.

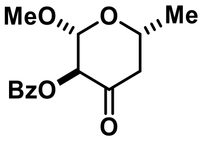

(2*R*,3*R*,4*S*,6*R*)-4-Hydroxy-2-methoxy-6-methyltetrahydro-2*H*-pyran-3-yl benzoate (24). In a 10-mL Schlenk tube, L-selectride® (1.0 M in THF, 0.15 mL, 0.15 mmol) was added dropwise to a solution of benzoyl ketol **23** (37.1 mg, 0.140 mmol) in THF (1.2 mL) at −78 °C. After stirring for 40 min at this temperature, ethyl acetate (3 mL) and saturated aqueous ammonium chloride solution (3 mL) were introduced, and the mixture was vigorously stirred for 30 min in a warm water bath (~30 °C). The layers were separated, and the aqueous phase was extracted with ethyl acetate (3 × 10 mL). The combined organic layers were dried over anhydrous sodium sulfate, the drying agent was filtered off, and the solvent was removed under reduced pressure. Purification of the residue by flash chromatography (hexanes/EtOAc, 3:1 → 2:1) furnished the title compound as a colorless syrup, which formed a white solid upon standing (27.8 mg, 75% yield). ^{1}H NMR (400 MHz, $CDCl_3$): δ 8.10 – 8.01 (m, 2H), 7.62 – 7.55 (m, 1H), 7.50 – 7.40 (m, 2H), 4.83 (dd, *J* = 9.2, 7.8 Hz, 1H), 4.43 (d, *J* = 7.7 Hz, 1H), 3.91 (ddd, *J* = 11.5, 9.2, 5.3 Hz, 1H), 3.66 (dqd, *J* = 12.8, 6.4, 2.2 Hz, 1H), 3.51 (s, 3H), 2.12 (ddd, *J* = 13.1, 5.3, 2.0 Hz, 1H), 1.57 (dt, *J* = 13.1, 11.4 Hz, 1H), 1.33 (d, *J* = 6.2 Hz, 3H).

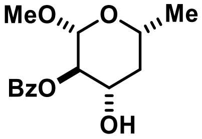

Oxime 25 and Hydrogenative Methylation Thereof.

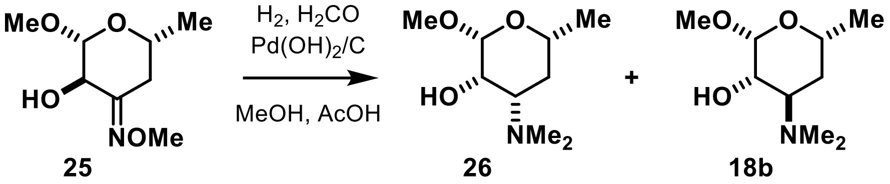

Methoxyamine hydrochloride (320 mg, 3.84 mmol) was added to a solution of ketol **21** (123 mg, 0.768 mmol; as a mixture with its dimer **22**) in methanol (1.0 mL) and pyridine (0.37 mL), and the resulting mixture was stirred at 45 °C (bath temperature) for 15 h. The mixture was then allowed to cool to room temperature before it was diluted with ethyl acetate (10 mL). The resulting white suspension was washed with saturated aqueous sodium bicarbonate solution (10 mL) and brine (10 mL). The aqueous layers were extracted with EtOAc (3 × 10 mL), the combined organic phases were dried over anhydrous sodium sulfate, the drying agent was filtered off, and the solvent was removed under reduced pressure. Remaining pyridine was azeotropically removed by co-evaporation twice with toluene, and the residue was purified by flash chromatography (hexane/EtOAc, 2:1) to furnish oxime **25** as a white solid (128 mg, 88% yield). ^{1}H NMR (400 MHz, $CDCl_3$): δ 4.18 (d, *J* = 7.7 Hz, 1H), 3.97 (d, *J* = 7.7 Hz, 1H), 3.90 (s, 3H), 3.62 – 3.53 (m, 1H), 3.59 (s, 3H), 3.21 (ddd, *J* = 14.8, 2.6, 0.6 Hz, 1H), 1.76 (ddd, *J* = 14.8, 11.4, 0.6 Hz, 1H), 1.33 (d, *J* = 6.1 Hz, 3H). ^{13}C{^{1}H} NMR (101 MHz, $CDCl_3$): δ 153.9, 105.9, 71.3, 69.3, 62.3, 57.0, 32.7, 21.3. IR (film): 3460, 2972, 2938, 2903, 1644, 1446, 1384, 1324, 1273, 1242, 1205, 1160, 1115, 1046, 1000, 965, 937, 885, 841, 683, 660 cm^{-1}.

A solution of oxime **25** (127 mg, 0.671 mmol) in methanol (2.0 mL) and then acetic acid (0.30 mL) were sequentially added to a suspension of $Pd(OH)_2/C$ (20% *w*/*w*, 48 mg, 68 µmol) in methanol (0.9 mL). After vigorous stirring for 30 h at room temperature under hydrogen atmosphere (balloon), TLC analysis (hexanes/EtOAc, 2:1) indicated only a small amount of remaining oxime. At this point, aqueous formaldehyde (37% *w*/*w*, 0.12 mL, 1.6 mmol) was introduced, and stirring continued for another 16 h under hydrogen atmosphere. The mixture was filtered through a short pad of Celite®, rinsing with methanol. The solvent and residual acetic acid were removed under reduced pressure (10^{-3} mbar), and the residue was subjected to flash chromatography (dichloromethane/methanol, 10:1, 0.3% *v*/*v* of triethylamine added). The product-containing fractions exhibited a single spot by TLC analysis (R_f = 0.13 with same solvent mixture), and furnished a light yellowish oil (122 mg). ^{1}H NMR analysis of this sample revealed a mixture of two compounds (ca. 3:1 ratio by integration), the major one of which was assigned as the isomerization product **26** (see Table S1).

Spectral data of the minor component, assigned as **18b**, from the mixture with **26***:* ^{1}H NMR (400 MHz, $CDCl_3$): δ 4.18 (d, *J* = 7.3 Hz, 1H), 3.60 – 3.52 (m, 1H), 3.56 (s, 3H), 3.26 (d, *J* = 10.3, 7.3 Hz, 1H), 2.60 – 2.49 (m, 1H), 2.30 (s, 6H), 1.72 (ddd, *J* = 12.7, 4.0, 2.0 Hz, 1H), 1.28 (d, *J* = 6.2 Hz, 3H), 1.26 – 1.20 (m, 1H). $^{13}C\{^1H\}$ NMR (101 MHz, $CDCl_3$): δ 105.1, 70.1, 69.8, 65.5, 56.8, 40.5, 28.8, 21.4.

Table S1. Assignment of the major isomer **26** obtained by hydrogenation/reductive methylation of oxime **25** by NMR spectroscopy (^{1}H: 400 MHz, $^{13}C\{^1H\}$: 101 MHz, $CDCl_3$). The decreased vicinal coupling between H1 and H2 and the small ^4J coupling (“W-shaped coupling”) between H2 and H4-eq are particularly indicative of stereo-inversion at C2.

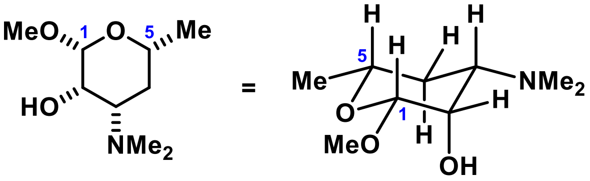

position	δ_H	multiplicity (*J*, Hz)	δ_C
1	4.69	d (3.4)	102.1
1-OMe	3.45	s	55.8
2	3.77	td (3.6, 0.9)	66.6
3	2.70	ddd (10.0, 4.9, 3.8)	57.2
3-NMe_2	2.35	s	42.8
4	ax, 2.00	ddd (13.6, 10.0, 5.6)	29.2
	eq, 1.54	dddd (13.4, 4.5, 4.1, 0.9)	
5	4.15	m	68.6
5-Me	1.34	d (6.8)	22.1

(2*R*,3*R*,4*R*,6*R*)-2-Methoxy-6-methyltetrahydro-2*H*-pyran-3,4-diol (27). In a 250-mL two-necked

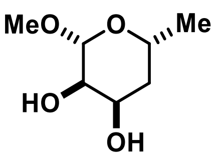

flask, aqueous hydrogen peroxide solution (35% *w*/*w*, 4.2 mL, 43 mmol) and aqueous sodium hydroxide solution (2 M, 0.90 mL) were sequentially added to a solution of compound **16** (2.07 g, 18.5 mmol)[2] in methanol (85 mL) at −45 °C. Stirring was continued at that temperature for 2 h before the mixture was neutralized with acetic acid (0.12 mL) at −45 °C. Trimethylphosphite (8.0 mL) was carefully added at this temperature and the mixture was allowed to warm to −20 °C over the course of 30 min. At this point, the same amount of trimethylphosphite was added again. After stirring for another 30 min at −20 °C, a peroxide test (Merck test strip) was negative. The mixture was warmed to ambient temperature and volatile components were

removed under reduced pressure (10^{-2} mbar, 30 °C). The residue was dissolved in ethyl acetate (40 mL) and the resulting solution was dried over anhydrous sodium sulfate. The drying agent was filtered off, and the solvent was removed under reduced pressure. Purification of the residue by flash chromatography (hexanes/EtOAc, 2:1) furnished a colorless gum (2.07 g), which was used in the next step without further characterization.

In a 500-mL two-necked flask, diisobutylaluminum hydride (25% *w*/*w* in toluene, 30 mL, 42 mmol) was added over 30 min to a solution of this material in tetrahydrofuran (70 mL) at −78 °C. The mixture was allowed to warm to room temperature over the course of 21 h. For work-up, the mixture was cooled in an ice bath, and the reaction was carefully quenched by the introduction of ethyl acetate (150 mL). Saturated aqueous Rochelle's salt solution (250 mL) was added, and the mixture was vigorously stirred at room temperature for 24 h to give a clear, biphasic mixture. The layers were separated and the aqueous phase was extracted with ethyl acetate (7 × 40 mL). The combined organic layers were dried over anhydrous sodium sulfate, the drying agent was filtered off, and the solvent was removed under reduced pressure. Purification of the residue by flash chromatography with hexanes/acetone (5:2) furnished the title compound as a colorless oil, which solidified upon standing at −20 °C (1.73 g, 58% yield). mp = 50 – 53 °C. $[\alpha]_D^{20}$ = −82.3 (*c* 0.53, $CHCl_3$). ^1H NMR (400 MHz, $CDCl_3$): δ 4.51 (d, *J* = 8.0 Hz, 1H), 4.16 (q, *J* = 3.2 Hz, 1H), 4.07 – 3.97 (m, 1H), 3.54 (s, 3H), 3.38 (dd, *J* = 8.0 Hz, 3.3 Hz, 1H), 2.53 (br s, 2H), 1.89 (ddd, *J* = 14.3, 3.3, 2.2 Hz, 1H), 1.52 (ddd, *J* = 14.3, 11.4, 2.8 Hz, 1H), 1.23 (d, *J* = 6.3 Hz, 3H). $^{13}C\{^1H\}$ NMR (101 MHz, $CDCl_3$): δ 101.5, 71.9, 67.6, 66.8, 57.0, 38.7, 20.8. IR (film): 3434, 2971, 2919, 2889, 2842, 1447, 1384, 1343, 1285, 1204, 1159, 1128, 1073, 1042, 922, 897, 828, 720, 646 cm^{-1}. HRMS-ESI *m*/*z*: $[M+Na]^+$ calcd for $C_7H_{14}O_4Na$ 185.0784; found 185.0787.

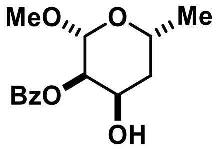

(2*R*,3*R*,4*R*,6*R*)-4-Hydroxy-2-methoxy-6-methyltetrahydro-2*H*-pyran-3-yl benzoate (28). In a 100-mL two-necked flask under argon, benzoyl chloride (1.24 mL, 10.7 mmol) was added to a solution of diol **27** (1.73 g, 10.7 mmol), 4-(dimethylamino)-pyridine (261 mg, 2.14 mmol) and pyridine (1.8 mL, 22 mmol) in dichloromethane (56 mL) at 0 °C (ice bath). The mixture was stirred for 24 h while allowing the cooling bath to warm to room temperature. For work-up, the mixture was diluted with dichloromethane (20 mL) and washed with a saturated aqueous solution of sodium bicarbonate (30 mL). The aqueous phase was extracted with dichloromethane (4 × 10 mL), the combined organic layers were dried over anhydrous sodium sulfate, the drying agent was filtered off, and the solvent was removed under reduced pressure. Purification of the residue by flash chromatography with hexanes/EtOAc (3:1) furnished the title compound as a colorless syrup (2.79 g, 92% yield). $[\alpha]_D^{20}$ = −47.5 (*c* 1.8, $CHCl_3$). ^1H NMR (400 MHz, $CDCl_3$): δ 8.10 – 8.00 (m, 2H), 7.62 – 7.55 (m, 1H), 7.50 – 7.42 (m, 2H), 4.94 (dd, *J* = 8.1, 3.0 Hz, 1H), 4.87 (d,

J = 8.1 Hz, 1H), 4.36 (q, J = 3.2 Hz, 1H), 4.19 – 4.08 (m, 1H), 3.51 (s, 3H), 2.13 (br s, 1H), 1.93 (ddd, J = 14.2, 3.7, 2.2 Hz, 1H), 1.69 (ddd, J = 14.1, 11.2, 2.7 Hz, 1H), 1.28 (d, J = 6.3 Hz, 3H). $^{13}C\{^1H\}$ NMR (101 MHz, $CDCl_3$): δ 165.5, 133.5, 129.9, 128.6, 99.4, 73.8, 67.2, 66.5, 56.8, 39.0, 20.9. IR (film): 2972, 2933, 2847, 1720, 1602, 1584, 1451, 1372, 1348, 1318, 1272, 1206, 1162, 1140, 1113, 1077, 1030, 1001, 905, 851, 712, 542 cm^{-1}. HRMS-ESI *m*/*z*: $[M+H]^+$ calcd for $C_{14}H_{19}O_5$ 267.1227; found 267.1225.

(2*R*,3*R*,4*R*,6*R*)-3-Hydroxy-2-methoxy-6-methyltetrahydro-2*H*-pyran-4-yl benzoate (30). In a 50-mL round-bottom flask equipped with a Dean-Stark trap, a mixture of diol **27** (204 mg, 1.26 mmol) and dibutyltin oxide (339 mg, 1.36 mmol) in toluene (30 mL) was stirred at reflux temperature for 20 h, with azeotropic removal of water. The resulting light amber, clear solution was cooled to room temperature and concentrated to a volume of 5 – 10 mL under reduced pressure. Benzoyl chloride (0.17 mL, 1.5 mmol) was added, and the mixture was stirred for 20 min. Volatile components were evaporated under reduced pressure, and the residue was purified by flash chromatography with hexanes/EtOAc (4:1 → 3:1 → 2:1) to furnish the title compound as a colorless syrup (269 mg, 80% yield). $[\alpha]_D^{20}$ = −64.3 (c 1.1, $CHCl_3$). ^1H NMR (400 MHz, $CDCl_3$): δ 8.09 – 8.03 (m, 2H), 7.61 – 7.55 (m, 1H), 7.49 – 7.42 (m, 2H), 5.60 (q, J = 3.2 Hz, 1H), 4.67 (d, J = 8.0 Hz, 1H), 4.08 – 3.98 (m, 1H), 3.61 (dt, J = 8.0, 3.4 Hz, 1H), 3.59 (s, 3H), 2.41 (d, J = 3.5 Hz, 1H), 2.03 (ddd, J = 14.7, 3.5, 2.1 Hz, 1H), 1.70 (ddd, J = 14.4, 11.4, 2.6 Hz, 1H), 1.26 (d, J = 6.2 Hz, 3H). $^{13}C\{^1H\}$ NMR (101 MHz, $CDCl_3$): δ 166.2, 133.4, 130.2, 129.8, 128.6, 102.0, 71.0, 67.2, 56.9, 37.5, 20.9. IR (film): 3485, 2973, 2932, 2874, 2844, 1717, 1602, 1584, 1451, 1383, 1354, 1315, 1275, 1206, 1160, 1117, 1068, 1040, 1028, 984, 715, 519 cm^{-1}. HRMS-ESI *m*/*z*: $[M+Na]^+$ calcd for $C_{14}H_{18}O_5Na$ 289.1046; found 289.1047.

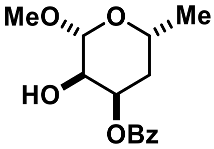

Tosylation of Diol 27 via a Stannylene Acetal.

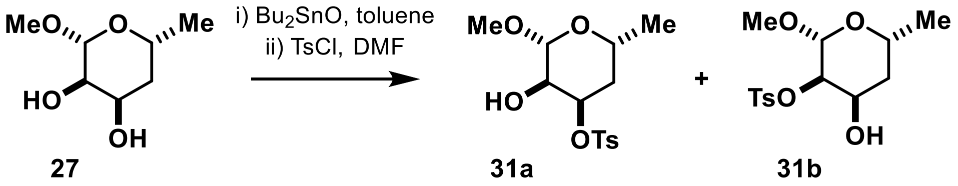

In a 50-mL round-bottom flask equipped with a Dean-Stark trap, a mixture of diol **27** (145 mg, 0.894 mmol) and dibutyltin oxide (240 mg, 0.964 mmol) in toluene (21 mL) was stirred at reflux for 19 h, with azeotropic removal of water. The resulting light amber, clear solution was cooled to room temperature and concentrated under reduced pressure. The residue was dissolved in DMF (4 mL), 4-toluenesulfonyl chloride (196 mg, 1.03 mmol) was added, and the mixture was stirred for 1 h. For work-up, the reaction mixture was diluted with *tert*-butyl methyl ether (30 mL) and washed with water (3 × 20 mL) and brine (20 mL). The aqueous

layers were extracted with *tert*-butyl methyl ether (3 × 10 mL), the combined organic phases were dried over anhydrous sodium sulfate, the drying agent was filtered off, and the solvent was removed under reduced pressure. The residue was purified by flash chromatography with hexanes/EtOAc (3:1 → 2:1 → 1:1), first eluting the equatorial tosylate **31b** (104 mg, 37% yield), followed by the axial tosylate **31a** (154 mg, 55% yield).

Data of compound **31a** (axial tosylate): colorless gum; ^{1}H NMR (400 MHz, $CDCl_3$): δ 7.86 – 7.81 (m, 2H), 7.37 – 7.32 (m, 2H), 4.95 (td, *J* = 3.5, 2.3 Hz, 1H), 4.48 (d, *J* = 8.0 Hz, 1H), 3.98 (dqd, *J* = 12.6, 6.3, 1.9 Hz, 1H), 3.51 (s, 3H), 3.42 – 3.36 (m, 1H), 2.45 (s, 3H), 2.13 (d, *J* = 4.4 Hz, 1H), 2.05 (ddd, *J* = 14.7, 3.7, 2.2 Hz, 1H), 1.61 – 1.51 (m, 1H), 1.21 (d, *J* = 6.3 Hz, 3H).

Data of compound **31b** (equatorial tosylate): colorless gum; ^{1}H NMR (400 MHz, $CDCl_3$): δ 7.83 – 7.76 (m, 2H), 7.36 – 7.29 (m, 2H), 4.57 (d, *J* = 7.9 Hz, 1H), 4.38 (br q, *J* = 3.1 Hz, 1H), 4.17 (dd, *J* = 7.9, 3.1 Hz, 1H), 4.06 – 3.95 (m, 1H), 3.19 (s, 3H), 2.44 (s, 3H), 1.91 (ddd, *J* = 14.3, 3.7, 2.1 Hz, 1H), 1.56 (ddd, *J* = 14.1, 11.3, 2.7 Hz, 1H), 1.18 (d, *J* = 6.2 Hz, 3H).

(2*R*,3*R*,4*R*,6*R*)-2-Methoxy-6-methyl-4-((methylsulfonyl)oxy)tetrahydro-2*H*-pyran-3-yl benzoate (S1).

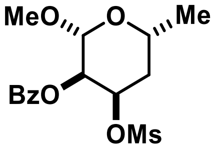

In a 100-mL two-necked flask under argon, methanesulfonyl chloride (1.1 mL, 14 mmol) and triethylamine (3.7 mL, 27 mmol) were sequentially added to a solution of alcohol **28** (2.79 g, 10.5 mmol) and 4-(dimethylamino)-pyridine (960 mg, 7.86 mmol) in dichloromethane (36 mL) at 0 °C (ice bath). After stirring for 10 min, the cooling bath was removed and stirring was continued for 1 h at room termperature. For work-up, saturated aqueous sodium bicarbonate solution (40 mL) was added and the resulting mixture was stirred for another hour at room temperature to destroy the excess of methanesulfonyl chloride. The mixture was diluted with EtOAc (40 mL), and the two layers were separated. The aqueous phase was extracted with ethyl acetate (4 × 15 mL), the combined organic layers were dried over anhydrous sodium sulfate, the drying agent was filtered off, and the solvent was removed under reduced pressure. The residue was purified by flash chromatography with hexanes/*tert*-butyl methyl ether (6:5) to furnish the title compound as a colorless foam (3.56 g, 99% yield). $[\alpha]_{D}^{20}$ = −57.5 (*c* 1.3, $CHCl_3$). ^{1}H NMR (400 MHz, $CDCl_3$): δ 8.09 – 8.03 (m, 2H), 7.62 – 7.55 (m, 1H), 7.49 – 7.43 (m, 2H), 5.27 (td, *J* = 3.5, 2.3 Hz, 1H), 4.94 (dd, *J* = 8.2, 3.2 Hz, 1H), 4.85 (d, *J* = 8.2 Hz, 1H), 4.18 – 4.07 (m, 1H), 3.53 (s, 3H), 2.89 (s, 3H), 2.18 (ddd, *J* = 14.8, 3.8, 2.1 Hz, 1H), 1.84 (ddd, *J* = 14.8, 11.1, 2.3 Hz, 1H), 1.31 (d, *J* = 6.3 Hz, 3H). ^{13}C{^{1}H} NMR (101 MHz, $CDCl_3$): δ 165.7, 133.7, 130.0, 129.5, 128.7, 99.2, 77.9, 71.1, 66.6, 56.9, 38.5, 38.3, 20.4. IR (film): 3069, 3029, 2973, 2937, 2881, 2849, 1723, 1602, 1585, 1451, 1404, 1353, 1317, 1274, 1207, 1177, 1163, 1132, 1110, 1070, 1030, 1002, 971, 909, 860, 814, 762, 713, 694, 665, 528, 514 cm^{-1}. HRMS-ESI *m/z*: $[M+Na]^+$ calcd for $C_{15}H_{20}O_7SNa$ 367.0822; found 367.0822.

(2*R*,3*R*,4*S*,6*R*)-4-Azido-2-methoxy-6-methyltetrahydro-2*H*-pyran-3-yl benzoate (29). In a 100-mL round-bottom flask, a mixture of mesylate **S1** (3.56 g, 10.3 mmol) and sodium azide (874 mg, 13.4 mmol) in DMF (50 mL) was stirred at 90 °C (bath temperature) for 15 h. For work-up, the mixture was diluted with *tert*-butyl methyl ether (200 mL) and washed with water (1 × 50 mL, 2 × 30 mL) and brine (30 mL). The combined water layers were extracted with *tert*-butyl methyl ether (1 × 50 mL, 2 × 30 mL), the organic phases were dried over anhydrous sodium sulfate, the drying agent was filtered off, and the solvent was removed under reduced pressure. Purification of the residue by flash chromatography with hexanes/EtOAc (11:2) furnished the title compound as a white, crystalline solid (2.47 g, 82% yield). mp = 102 – 103 °C. $[\alpha]_D^{20}$ = +47.6 (*c* 0.37, $CHCl_3$). ^1H NMR (400 MHz, $CDCl_3$): δ 8.09 – 8.05 (m, 2H), 7.62 – 7.55 (m, 1H), 7.49 – 7.43 (m, 2H), 5.03 (dd, *J* = 10.0, 7.8 Hz, 1H), 4.42 (d, *J* = 7.8 Hz, 1H), 3.76 – 3.66 (m, 2H), 3.46 (s, 3H), 2.09 (ddd, *J* = 13.1, 5.0, 1.9 Hz, 1H), 1.65 – 1.54 (m, 1H), 1.34 (d, *J* = 6.2 Hz, 3H). $^{13}C\{^1H\}$ NMR (101 MHz, $CDCl_3$): δ 165.5, 133.4, 130.0, 129.9, 128.5, 102.2, 73.7, 68.8, 60.6, 56.8, 37.9, 20.9. IR (film): 2977, 2934, 2872, 2844, 2106, 1728, 1450, 1391, 1370, 1338, 1314, 1305, 1275, 1215, 1190, 1164, 1143, 1106, 1071, 1028, 994, 705 cm^{-1}. HRMS-ESI *m*/*z*: $[M+Na]^+$ calcd for $C_{14}H_{17}O_4Na$ 314.1111; found 314.1108.

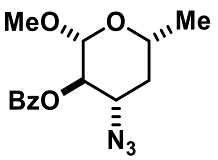

Methyl 2-O-benzoyl *β*-D-desosaminopyranoside (18c). In a 250-mL two-necked flask under hydrogen atmosphere (balloon), a solution of azide **29** (2.47 g, 8.48 mmol) in a mixture of ethyl acetate/ethanol (8.0 mL/10 mL; syringe and flask rinsed with ethanol, 2 × 10 mL) was added to a suspension of $Pd(OH)_2/C$ (20% *w*/*w*, 595 mg, 0.168 mmol) in ethanol (10 mL) at ambient temperature. After vigorous stirring for 1.5 h, TLC analysis (hexanes/EtOAc, 4:1) indicated full consumption of the azide. At this point, aqueous formaldehyde (37% *w*/*w*, 1.4 mL, 19 mmol) was added, and the mixture was stirred for further 17 h under hydrogen atmosphere. The mixture was filtered through a short pad of Celite®, rinsing with dichloromethane. The solvent was removed under reduced pressure and the residue was purified by flash chromatography with dichloromethane/methanol (25:1, 0.2% *v*/*v* of triethylamine added) to furnish the title compound as a colorless gum (2.44 g, 98% yield). $[\alpha]_D^{20}$ = −1.9 (*c* 1.1, $CHCl_3$). ^1H NMR (400 MHz, $CDCl_3$): δ 8.10 – 8.02 (m, 2H), 7.59 – 7.52 (m, 1H), 7.49 – 7.40 (m, 2H), 5.10 (dd, *J* = 10.5, 7.5 Hz, 1H), 4.39 (d, *J* = 7.5 Hz, 1H), 3.65 (dqd, *J* = 10.9, 6.1, 2.0 Hz, 1H), 3.45 (s, 3H), 3.03 – 2.91 (m, 1H), 2.33 (s, 6H), 1.95 – 1.81 (m, 1H), 1.54 – 1.42 (m, 1H), 1.32 (d, *J* = 6.1 Hz, 3H). $^{13}C\{^1H\}$ NMR (101 MHz, $CDCl_3$): δ 165.8, 133.0, 130.6, 130.0, 128.4, 103.4, 71.3, 69.5, 63.5, 56.8, 40.9, 32.1, 21.3. IR (film): 2971, 2937, 2866, 2833, 2782, 1721, 1602, 1584, 1451, 1389, 1342, 1314, 1294, 1271, 1228, 1164, 1124, 1108, 1068, 1053, 997, 975, 937, 888, 869, 840, 802, 710, 636, 549 cm^{-1}. HRMS-ESI *m*/*z*: $[M+H]^+$ calcd for $C_{16}H_{24}NO_4$ 294.1700; found 294.1705.

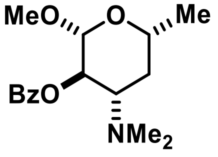

1-O-Acetyl-2-O-benzoyl D-desosaminopyranose (18d). In a round-bottom flask, a solution of concentrated sulfuric acid in acetic anhydride (1:33 *v*/*v*, 1.6 mL) was added to the methyl glycoside **18c** (213 mg, 0.726 mmol), and the mixture was stirred for 24 h at room temperature. For work up, the mixture was diluted with ethyl acetate (15 mL) and carefully poured into saturated aqueous sodium bicarbonate solution (15 mL). The layers were separated and the aqueous phase was extracted with ethyl acetate (3 × 10 mL). The combined organic layers were dried over anhydrous sodium sulfate, the drying agent was filtered off, and the filtrate was concentrated under reduced pressure. Most of the remaining acetic anhydride was evaporated under high vacuum. Purification of the residue by flash chromatography (dichloromethane/methanol, 35:1, 0.2% *v*/*v* of triethylamine added) furnished the title compound as a colorless gum (203 mg, 87% yield; ca. 6:1 mixture of α/β anomers). $[\alpha]_D^{20}$ = +91.3 (*c* 1.0, $CHCl_3$). ^1H NMR (400 MHz, $CDCl_3$): signals of the α-anomer: δ 8.03 – 7.96 (m, 2H), 7.59 – 7.53 (m, 1H), 7.47 – 7.39 (m, 2H), 6.34 (d, *J* = 3.7 Hz, 1H), 5.31 (dd, *J* = 11.0, 3.7 Hz, 1H), 4.10 (dqd, *J* = 12.2, 6.2, 2.2 Hz, 1H), 3.42 – 3.25 (m, 1H), 2.37 (br s, 6H), 2.13 (s, 3H), 2.02 – 1.91 (m, 1H), 1.60 – 1.48 (m, 1H), 1.25 (d, *J* = 6.2 Hz, 3H); signals of the β-anomer: δ 8.03 – 7.96 (m, 2H), 7.59 – 7.53 (m, 1H), 7.47 – 7.39 (m, 2H), 5.77 (d, *J* = 8.0 Hz, 1H), 5.20 (dd, *J* = 10.5, 8.0 Hz, 1H), 3.80 (dqd, *J* = 12.4, 6.2, 2.1 Hz, 1H), 3.04 – 2.92 (m, 1H), 2.32 (br s, 6H), 1.98 (s, 3H), 1.92 – 1.83 (m, 1H), 1.55 – 1.45 (m, 1H), 1.32 (d, *J* = 6.2 Hz, 3H). $^{13}C\{^1H\}$ NMR (101 MHz, $CDCl_3$): signals of mixed anomers reported collectively: δ 169.7, 169.5, 165.7, 165.6, 133.4, 133.2, 130.1, 129.93, 129.87, 128.58, 128.55, 93.84, 90.85, 70.8, 70.5, 69.4, 67.4, 63.5, 58.4, 40.9, 33.1, 31.4, 21.21, 21.16, 21.1. IR (film): 2974, 2939, 2870, 2833, 2782, 1750, 1721, 1602, 1584, 1452, 1373, 1316, 1272, 1228, 1161, 1138, 1113, 1080, 1070, 1044, 1011, 980, 943, 924, 873, 857, 803, 712, 600, 564, 507 cm^{-1}. HRMS-ESI *m*/*z*: $[M+H]^+$ calcd for $C_{17}H_{24}NO_5$ 322.1649; found 322.1645.

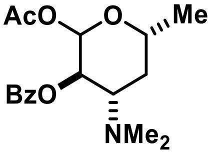

D-Desosamine (18a). In a round-bottom flask, anhydrous potassium carbonate (34.0 mg, 0.246 mmol) was added to a solution of compound **18d** (38.6 mg, 0.120 mmol) in methanol (1.2 mL), and the mixture was stirred vigorously for 1 h at room temperature. The reaction mixture was filtered through a short plug of silica gel (3 cm × 2.5 cm), rinsing with dichloromethane/methanol (20:1, 20 mL) followed by methanol (90 mL) to elute the product. The combined filtrates were concentrated under reduced pressure to furnish the title compound as a pale yellowish oil (20.2 mg, 96% yield; 1:2 mixture of α/β anomers). $[\alpha]_D^{25}$ = +57.9 (*c* 1.4, $CHCl_3$, after equilibration for ~90 min at 25 °C). ^1H NMR (400 MHz, [D4]-methanol): α-anomer: δ 5.09 (d, *J* = 3.6 Hz, 1H), 4.17 – 4.07 (m, 1H), 3.53 (dd, *J* = 10.6, 3.6 Hz, 1H), 2.96 (ddd, *J* = 12.3, 10.6, 4.0 Hz, 1H), 2.34 (s, 6H), 1.80 – 1.72 (m, 1H), 1.33 – 1.22 (m, 1H), 1.14 (d, *J* = 6.2 Hz, 3H); β-anomer: δ 4.42 (d, *J* = 7.4 Hz, 1H), 3.61 (dqd, *J* = 12.4, 6.2, 2.0 Hz, 1H), 3.20 (dd, *J* = 10.2, 7.4 Hz, 1H), 2.61 (ddd, *J* = 12.3, 10.2, 4.2 Hz, 1H), 2.33 (s, 6H), 1.80 – 1.72 (m, 1H), 1.31 – 1.17 (m, 1H), 1.22 (d, *J* = 6.2 Hz, 3H). $^{13}C\{^1H\}$ NMR

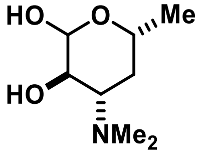

(101 MHz, [D_4]-methanol): α-anomer: δ 94.4, 70.8, 65.3, 60.8, 40.7, 33.1, 21.5; β-anomer: δ 99.4, 72.9, 70.5, 65.7, 40.9, 32.1, 21.5. IR (film): 3366, 2970, 2935, 2871, 2835, 2786, 1654, 1457, 1382, 1321, 1279, 1163, 1096, 1042, 990, 975, 937, 867, 854, 833, 806, 752, 723, 634, 569, 421 cm^{-1}. HRMS-ESI *m*/*z*: $[M+H]^+$ calcd for $C_8H_{18}NO_3$ 176.1281; found 176.1281.

2-O-Benzoyl D-desosamine (S2). In a round-bottom flask, a solution of concentrated sulfuric acid in

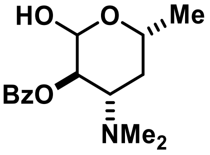

acetic anhydride (1:33 *v*/*v*, 15 mL) was added to methyl glycoside **18c** (2.44 g, 8.32 mmol), and the mixture was stirred for 24 h at room temperature. For work up, the mixture was diluted with ethyl acetate (30 mL) and carefully poured into saturated aqueous sodium bicarbonate solution (50 mL). The layers were separated and the organic phase was washed with saturated aqueous sodium bicarbonate solution (2 × 20 mL) and brine (20 mL). The combined aqueous layers were extracted with ethyl acetate (5 × 25 mL). The combined organic layers were dried over anhydrous sodium sulfate, the drying agent was filtered off, and the filtrate was concentrated under reduced pressure. Most of the remaining acetic anhydride was evaporated under high vacuum. Purification of the residue by flash chromatography (dichloromethane/methanol, 35:1, 0.2% *v*/*v* of triethylamine added) furnished the intermediate glycosyl acetate admixed with a small amount of the remaining methyl glycoside (2.77 g, light yellowish gum).

In a round-bottom flask, a solution of ammonia in methanol (7 M, 59 mL) was added to a solution of this material in THF (12 mL) at 0 °C (ice bath), and the reaction mixture was stirred at that temperature for 5 h. The mixture was then concentrated under reduced pressure to give a light yellowish gum. Purification of the residue by flash chromatography (hexanes/acetone, 2:1, 0.1% *v*/*v* of triethylamine added) furnished the pure title compound as a colorless foam (1.96 g, 85% yield; ca. 1:1 mixture of α/β anomers). $[\alpha]_D^{20}$ = +43.1 (*c* 0.71, MeOH). ^{1}H NMR (400 MHz, $CDCl_3$): α-anomer: δ 8.11 – 8.03 (m, 2H), 7.62 – 7.54 (m, 1H), 7.49 – 7.41 (m, 2H), 5.43 (d, *J* = 3.6 Hz, 1H), 5.18 (dd, *J* = 11.0, 3.6 Hz, 1H), 4.24 (dqd, *J* = 12.5, 6.3, 2.3 Hz, 1H), 3.62 – 3.45 (m, 1H), 2.44 (br s, 6H), 2.14 – 2.00 (m, 1H), 1.55 – 1.43 (m, 1H), 1.22 (d, *J* = 6.2 Hz, 3H); β-anomer: δ 8.11 – 8.03 (m, 2H), 7.62 – 7.54 (m, 1H), 7.49 – 7.41 (m, 2H), 5.00 (dd, *J* = 10.4, 7.6 Hz, 1H), 4.68 (d, *J* = 7.6 Hz, 1H), 3.68 (dqd, *J* = 10.9, 6.1, 2.0 Hz, 1H), 3.10 – 2.98 (m, 1H), 2.35 (br s, 6H), 1.96 – 1.85 (m, 1H), 1.55 – 1.43 (m, 1H), 1.30 (d, *J* = 6.2 Hz, 3H). ^{13}C{^{1}H} NMR (101 MHz, $CDCl_3$): signals of mixed anomers reported collectively: δ 167.2, 166.0, 133.4, 130.1, 130.0, 128.6, 128.5, 97.1, 91.3, 74.0, 71.5, 69.8, 64.6, 62.8, 57.6, 41.1, 40.9, 33.8, 33.0, 21.3, 21.2. IR (film): 3249, 2971, 2933, 2782, 1716, 1602, 1584, 1451, 138, 1368, 1315, 1269, 1160, 1121, 1057, 1028, 997, 937, 886, 839, 804, 711, 688, 634, 588 cm^{-1}. HRMS-ESI *m*/*z*: $[M+H]^+$ calcd for $C_{15}H_{22}NO_4$ 280.1543; found 280.1543.

2-O-Benzoyl α-D-desosaminopyranosyl fluoride (18e). In a 60-mL Nalgene™ screw-capped reaction vessel, hydrogen fluoride pyridine complex (70% HF *w*/*w*, 1.5 mL) was added to a solution of acetate **18d** (185 mg, 0.576 mmol) in dichloromethane (6.0 mL) at 0 °C (ice bath). The reaction mixture, which turned pale yellowish upon the addition, was stirred for 3 h at 0 °C. For work-up, the cold mixture was carefully poured into a saturated aqueous solution of sodium bicarbonate (30 mL). The layers were separated and the aqueous phase was extracted with dichloromethane (4 × 10 mL). The combined organic layers were dried over anhydrous sodium sulfate, the drying agent was filtered off, and the solvent was removed under reduced pressure. The residue was purified by flash chromatography (hexanes/acetone, 7:1, 0.1% *v*/*v* of triethylamine added) to furnish the title compound as a colorless oil (108 mg, 67% yield). $[\alpha]_D^{20}$ = +108.6 (*c* 1.0, $CHCl_3$). ^{1}H NMR (400 MHz, $CDCl_3$): δ 8.12 – 8.07 (m, 2H), 7.62 – 7.55 (m, 1H), 7.50 – 7.43 (m, 2H), 5.74 (dd, *J* = 54.5, 2.7 Hz, 1H), 5.19 (ddd, *J* = 23.9, 11.0, 2.8 Hz, 1H), 4.21 (dqd, *J* = 12.3, 6.3, 2.3 Hz, 1H), 3.46 – 3.29 (br m, 1H), 2.37 (br s, 6H), 2.09 – 1.90 (br m, 1H), 1.55 (q, *J* = 12.4 Hz, 1H), 1.29 (d, *J* = 6.2 Hz, 3H). ^{13}C{^{1}H} NMR (101 MHz, $CDCl_3$): δ 166.0, 133.5, 130.1, 129.8, 128.6, 105.8 (d, *J* = 224 Hz), 70.3 (d, *J* = 25.6 Hz), 67.7 (d, *J* = 4.0 Hz), 57.9, 40.9, 33.0, 21.0. ^{19}F NMR (282 MHz, $CDCl_3$): δ −147.2 (dd, *J* = 54.6, 24.1 Hz). IR (film): 2976, 2941, 2867, 2834, 2781, 1721, 1602, 1452, 1390, 1332, 1271, 1221, 1197, 1168, 1116, 1070, 1050, 1035, 982, 924, 875, 797, 711, 579 cm^{-1}. HRMS-EI *m*/*z*: $[M]^+$ calcd for $C_{15}H_{20}NO_3F$ 281.1422; found 281.1420.

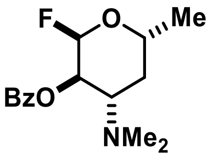

2-O-Benzoyl D-desosaminopyranosyl trichloroacetimidate (18f). Trichloroacetonitrile (0.34 mL, 3.4 mmol) and DBU (25 µL, 0.17 mmol) were sequentially added to a solution of lactol **S2** (234 mg, 0.838 mmol) in dichloromethane (4.0 mL) at room temperature. The originally colorless solution turned light brown upon the addition of DBU and the color intensified over the course of the reaction. After 1 h, the mixture was concentrated under reduced pressure. The residue was immediately subjected to flash chromatographic purification (hexanes/acetone 5:1 → 4:1, 0.2% *v*/*v* of triethylamine added) to furnish the title compound as a pale yellowish gum (283 mg, 80% yield). $[\alpha]_D^{20}$ = +36.2 (*c* 0.40, $CHCl_3$). ^{1}H NMR (400 MHz, $CDCl_3$): δ 8.53 (s, 1H), 8.02 – 7.97 (m, 2H), 7.55 – 7.50 (m, 1H), 7.44 – 7.37 (m, 2H), 5.89 (d, *J* = 7.7 Hz, 1H), 5.39 (dd, *J* = 10.3, 7.6 Hz, 1H), 3.87 (dqd, *J* = 12.4, 6.1, 2.1 Hz, 1H), 3.04 (ddd, *J* = 12.3, 10.3, 4.3 Hz, 1H), 2.34 (s, 6H), 1.93 – 1.84 (m, 1H), 1.66 – 1.55 (m, 1H), 1.36 (d, *J* = 6.1 Hz, 3H). ^{13}C{^{1}H} NMR (101 MHz, $CDCl_3$): δ 165.5, 161.8, 133.0, 130.4, 129.8, 128.4, 98.1, 90.9, 71.0, 70.5, 63.4, 40.9, 31.5, 21.3. ^{1}H NMR (400 MHz, C_6D_6): δ 8.54 (s, 1H), 8.21 – 8.15 (m, 2H), 7.08 – 6.94 (m, 3H), 6.21 (d, *J* = 7.9 Hz, 1H), 5.63 (dd, *J* = 10.6, 7.9 Hz, 1H), 3.40 (dqd, *J* = 12.3, 6.1, 2.2 Hz, 1H), 2.72 (ddd, *J* = 12.2, 10.5, 4.3 Hz, 1H), 2.10 (s, 6H), 1.28 (ddd, *J* = 13.1, 4.4, 2.2 Hz, 1H), 1.19 – 1.11 (m, 1H), 1.07 (d, *J* = 6.1 Hz, 3H). ^{13}C{^{1}H} NMR (101 MHz, C_6D_6): δ 165.5, 162.0, 132.8, 131.1, 130.0, 128.5, 128.4, 98.4,

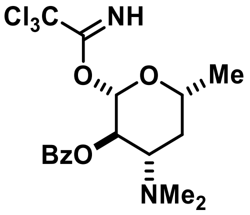

91.5, 70.8, 63.8, 40.7, 30.7, 21.1. IR (film): 3340, 2975, 2936, 2868, 2833, 2783, 1726, 1674, 1602, 1584, 1452, 1375, 1343, 1292, 1268, 1198, 1164, 1124, 1106, 1062, 1025, 934, 828, 796, 710, 646, 631, 579, 564 cm^{-1}. HRMS-ESI *m*/*z*: $[M+H]^+$ calcd for $C_{17}H_{22}N_2O_4Cl_3$ 423.0640; found 426.0638.

Completion of the Total Synthesis

Compound 33. Aqueous HCl (1.5 M, 0.20 mL, 0.30 mmol) was added to a solution of compound **15** (5.2 mg, 8.0 µmol)[3] in methanol (0.84 mL) at ambient temperature. After stirring for 8 h, the solution was diluted with water (3 mL) and ethyl acetate (3 mL), and vigorously stirred for 2 min. The layers were separated and the aqueous layer was extracted with ethyl acetate (3 x 2 mL). The combined organic layers were washed with saturated aqueous NaCl (2 mL), filtered through anhydrous sodium sulfate, and the filtrate was evaporated. Purification of the crude product by flash chromatography (silica, 15–40 µm particle size; loading as solution in CH_2Cl_2/hexanes = 1:1, eluting with EtOAc/hexanes = 1:2 to 1:1) afforded the title compound as a colourless solid material (1.7 mg, 52%). ^{1}H NMR (400 MHz, $CDCl_3$) δ 7.12 (ddd, *J* = 15.1, 11.0, 0.8 Hz, 1H), 6.61 (dd, *J* = 15.5, 9.9 Hz, 1H), 6.27–6.14 (m, 2H), 5.93 (dd, *J* = 15.2, 9.5 Hz, 1H), 5.77 (dd, *J* = 15.5, 0.7 Hz, 1H), 4.87 (ddd, *J* = 10.1, 9.0, 2.8 Hz, 1H), 4.68 (d, *J* = 6.6 Hz, 1H), 4.64 (d, *J* = 6.5 Hz, 1H), 3.80 (dd, *J* = 10.8, 4.1 Hz, 1H), 3.74 (dd, *J* = 10.8, 6.9 Hz, 1H), 3.41 (s, 3H), 3.15 (dd, *J* = 10.3, 1.5 Hz, 1H), 2.67–2.52 (m, 2H), 2.47 (tdd, *J* = 10.1, 6.8, 4.1 Hz, 1H), 1.83 (dqd, *J* = 14.7, 7.4, 2.8 Hz, 1H), 1.62–1.46 (m, 3H), 1.34–1.27 (m, 1H), 1.18 (d, *J* = 6.9 Hz, 3H), 1.10 (d, *J* = 6.7 Hz, 3H), 0.99 (d, *J* = 6.8 Hz, 3H), 0.96 (t, *J* = 7.6 Hz, 3H). ^{13}C NMR (101 MHz, $CDCl_3$) δ 203.3, 166.1, 151.4, 141.7, 140.6, 134.1, 123.5, 121.2, 99.3, 88.6, 73.5, 62.2, 56.3, 51.6, 44.6, 40.4, 34.2, 32.5, 25.4, 19.5, 17.6, 17.6, 9.6. HRMS-ESI *m*/*z*: $[M+Na]^+$ calcd for $C_{23}H_{36}O_6Na^+$: 431.2404, found: 431.2402.

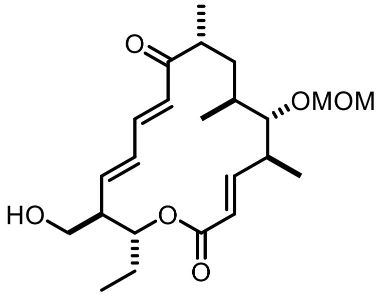

Compound 34. In a 10-mL Schlenk tube under argon, a solution of dimethylboron bromide in dichloromethane (0.80 M, 0.19 mL, 0.15 mmol) was added dropwise to a solution of MOM ether **15** (32.7 mg, 50.5 µmol)[3] in dichloromethane (1.0 mL) at −78 °C. After 1 h, a mixture of tetrahydrofuran (4 mL) and an aqueous solution of sodium carbonate (5% *w*/*w*, 4 mL) was introduced at that temperature. The mixture was warmed to room temperature by immersing the flask into a warm water bath, and vigorous stirring was continued for another 2 h. The mixture was diluted with ethyl acetate (10 mL) and washed with saturated aqueous sodium bicarbonate solution (10 mL). The aqueous phase was extracted with ethyl acetate (4 × 10 mL), the combined organic layers were dried over anhydrous sodium sulfate, the drying agent was filtered off, and the solvent was removed under reduced pressure.

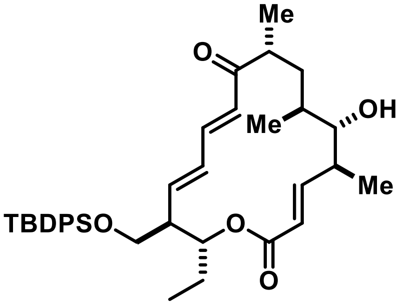

Purification of the residue by flash chromatography with hexanes/EtOAc (5:2) furnished the title compound as a colorless foam (24.4 mg, 80% yield). $[\alpha]_D^{20}$ = +47.6 (*c* 0.45, $CHCl_3$). ^{1}H NMR (400 MHz, $CDCl_3$): δ 7.69 – 7.59 (m, 4H), 7.48 – 7.35 (m, 6H), 7.18 – 7.10 (m, 1H), 6.60 (dd, *J* = 15.5, 9.9 Hz, 1H), 6.23 (d, *J* = 15.1 Hz, 1H), 6.15 – 6.04 (m, 2H), 5.77 (dd, *J* = 15.6, 0.7 Hz, 1H), 4.99 (ddd, *J* = 10.1, 9.0, 2.7 Hz, 1H), 3.79 – 3.69 (m, 2H), 3.29 (d, *J* = 10.3 Hz, 1H), 2.62 – 2.45 (m, 2H), 2.39 – 2.29 (m, 1H), 1.73 – 1.61 (m, 1H), 1.58 – 1.35 (m, 4H), 1.34 – 1.24 (m, 1H), 1.19 (d, *J* = 6.9 Hz, 3H), 1.12 (d, *J* = 6.7 Hz, 3H), 1.07 (s, 9H), 1.00 (d, *J* = 6.7 Hz, 3H), 0.87 (t, *J* = 7.3 Hz, 3H). ^{13}C{^{1}H} NMR (101 MHz, $CDCl_3$): δ 203.6, 166.3, 151.2, 142.5, 141.8, 135.8, 135.8, 133.33, 133.31, 132.95, 130.01, 129.98, 127.9, 123.0, 121.4, 80.4, 73.9, 62.9, 51.3, 45.0, 40.6, 33.9, 31.8, 27.0, 25.2, 19.5, 19.4, 17.9, 17.5, 9.88. IR (film): 3487, 3071, 2962, 2932, 2877, 2858, 1715, 1679, 1652, 1632, 1594, 1460, 1428, 1380, 1351, 1327, 1264, 1225, 1174, 1149, 1111, 988, 929, 8823, 790, 743, 703, 609, 504 cm^{-1}. HRMS-ESI *m/z*: $[M+Na]^+$ calcd for $C_{37}H_{50}O_5SiNa$ 625.3320; found 625.3323.

Compound 35. In a 10-mL Schlenk tube under argon, a solution of *tert*-butyldimethylsilyl trifluoromethanesulfonate in dichloromethane (0.088 M, 0.46 mL, 41 µmol) was added dropwise to a solution of alcohol **34** (24.4 mg, 40.5 µmol) and trichloro-acetimidate **18f** (129 mg, 0.304 mmol) in dichloromethane (1.3 mL) at room temperature. After stirring for 1.5 h, an additional amount of the silyl triflate stock solution (0.69 mL, 61 µmol) was added; the addition of silyl triflate addition (0.23 mL, 20 µmol) was repeated one more time after stirring for another 2.5 h. After further stirring for 1.5 h after the last addition, saturated aqueous sodium bicarbonate solution (10 mL) was introduced and the mixture was diluted with ethyl acetate (10 mL). The layers were separated and the aqueous phase was extracted with ethyl acetate (5 × 10 mL). The combined organic layers were dried over anhydrous sodium sulfate, the drying agent was filtered off, and the solvent was removed under reduced pressure. Purification of the residue by flash chromatography with hexanes/acetone (5:1, 0.1% *v*/*v* of triethylamine added) furnished the intermediate glycosylation product admixed with trichloroacetamide (a byproduct from glycosyl donor activation) as a light yellowish gum.

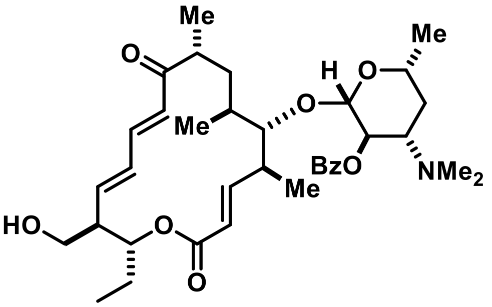

In a 10-mL Schlenk tube under argon, a solution of TBAF in THF (0.50 M, 0.17 mL, 85 µmol) was added to a solution of the material described above in THF (0.63 mL) at room temperature. After stirring for 1 h, the mixture was diluted with ethyl acetate (10 mL) and washed with saturated aqueous sodium bicarbonate solution (10 mL). The aqueous phase was extracted with ethyl acetate (5 × 10 mL), the combined organic layers were dried over anhydrous sodium sulfate, the drying agent was filtered off, and the solvent was

removed under reduced pressure. Purification of the residue by flash chromatography with hexanes/acetone (2:1, 0.1% *v*/*v* of triethylamine added) furnished the title compound as a white, amorphous solid (21.4 mg, 84% yield). $[\alpha]_D^{20}$ = +38.3 (*c* 0.41, $CHCl_3$). ^1H NMR (400 MHz, $CDCl_3$): δ 8.11 – 8.04 (m, 2H), 7.67 – 7.58 (m, 1H), 7.53 – 7.44 (m, 2H), 7.06 (dd. *J* = 15.1, 10.9 Hz, 1H), 6.48 (dd, *J* = 15.5, 9.9 Hz, 1H), 6.15 (d, *J* = 15.1 Hz, 1H), 6.09 (dd, *J* = 15.3, 10.9 Hz, 1H), 5.91 (dd, *J* = 15.3, 9.3 Hz, 1H), 5.59 (d, *J* = 15.5 Hz, 1H), 5.32 – 5.20 (br m, 1H), 4.85 (td, *J* = 9.6, 2.8 Hz, 1H), 4.57 (d, *J* = 7.2 Hz, 1H), 3.81 – 3.55 (m, 4H), 3.26 (d, *J* = 10.1 Hz, 1H), 2.83 – 2.48 (br m, 7H), 2.44 – 2.33 (m, 2H), 1.87 – 1.72 (m, 2H), 1.64 – 1.36 (m, 4H), 1.31 (d, *J* = 6.1 Hz, 3H), 1.25 – 1.19 (m, 1H), 1.14 (d, *J* = 6.9 Hz, 3H), 0.97 (d, *J* = 6.8 Hz, 3H), 0.93 – 0.83 (m, 6H). $^{13}C\{^1H\}$ NMR (101 MHz, $CDCl_3$): δ 203.7, 166.2, 165.3, 150.9, 141.7, 140.8, 134.0, 130.15, 130.06, 128.8, 123.8, 121.3, 101.8, 87.5, 73.8, 69.7, 68.6, 64.1, 62.1, 51.6, 44.8, 40.7, 34.2, 32.3, 31.9, 25.4, 20.8, 19.4, 17.8, 17.5, 9.8. IR (film): 3343, 2968, 2933, 2878, 1716, 1678, 1653, 1593, 1453, 1375, 1353, 1323, 1265, 1178, 1112, 1066, 1029, 999, 1066, 937, 755, 712 cm^{-1}. HRMS-ESI *m*/*z*: $[M+H]^+$ calcd for $C_{36}H_{52}NO_8$ 626.3687; found 626.3688.

When the glycosylation procedure described above was conducted with trimethyl- or triethylsilyl triflate as activator *added at or below 0 °C*, followed by warming of the mixture to room temperature, an unstable product was formed that could be isolated in ca. 30% yield (with TMSOTf, addition at −30 °C). By comparison to the literature precedence,[4] structure **36** could be assigned to this side product. The compound decomposed upon attempted re-purification of a small sample by flash chromatography. Moreover, the compound was not stable enough in $CDCl_3$ to deliver a satisfying $^{13}C\{^1H\}$ NMR spectrum by routine measurement overnight.

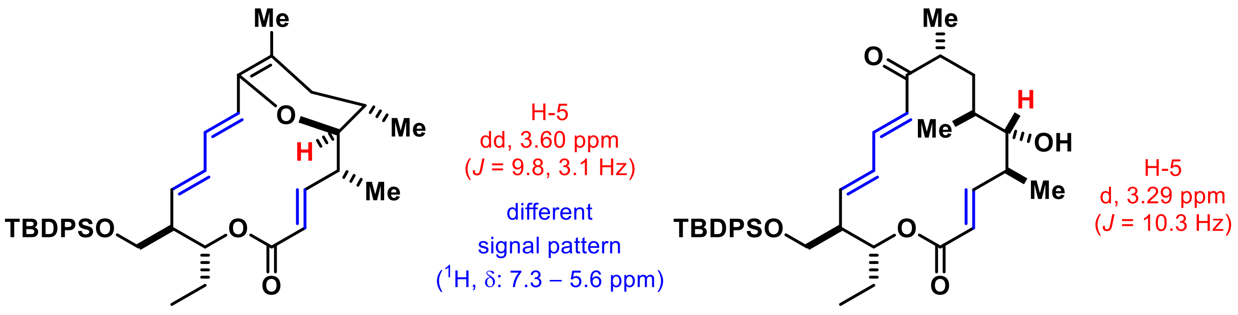

36 R_f = 0.27 (hexanes/EtOAc, 25:1)

34 R_f = 0.24 (hexanes/EtOAc, 5:2)

Following the same glycosylation procedure with either trimethyl- or triethylsilyl triflate as activator *added at room temperature*, the major product resulted from silylation of the acceptor alcohol (44% yield of **37** (R = Me).

Data of Compound **37a** (R = Me): ^{1}H NMR (400 MHz, $CDCl_3$): δ 7.68 – 7.60 (m, 4H), 7.48 – 7.36 (m, 6H), 7.18 – 7.09 (m, 1H), 6.60 (dd, *J* = 15.5, 10.0 Hz, 1H), 6.22 (d, *J* = 15.1 Hz, 1H), 6.16 – 6.02 (m, 2H), 5.74 (dd, *J* = 15.5, 0.7 Hz, 1H), 4.98 (ddd, *J* = 10.1, 9.0, 2.7 Hz, 1H), 3.79 – 3.69 (m, 2H), 3.32 (dd, *J* = 10.1, 1.3 Hz, 1H), 2.59 – 2.43 (m, 2H), 2.39 – 2.28 (m, 1H), 1.73 – 1.60 (m, 1H), 1.54 – 1.35 (m, 2H), 1.18 (d, *J* = 6.9 Hz, 3H), 1.06 (s, 9H), 1.03 (d, *J* = 6.8 Hz, 3H), 0.91 (d, *J* = 6.8 Hz, 3H), 0.90 – 0.82 (m, 5H), 0.13 (s, 9H).

Data of Compound **37b** (R = Et): ^{1}H NMR (400 MHz, $CDCl_3$): δ 7.68 – 7.58 (m, 4H), 7.48 – 7.35 (m, 6H), 7.17 – 7.08 (m, 1H), 6.60 (dd, *J* = 15.5, 10.0 Hz, 1H), 6.21 (d, *J* = 15.1 Hz, 1H), 6.15 – 6.01 (m, 2H), 5.72 (d, *J* = 15.5 Hz, 1H), 5.02 – 4.94 (m, 1H), 3.79 – 3.68 (m, 2H), 3.36 (d, *J* = 9.8 Hz, 1H), 2.60 – 2.43 (m, 2H), 2.39 – 2.28 (m, 1H), 1.72 – 1.59 (m, 1H), 1.54 – 1.35 (m, 2H), 1.17 (d, *J* = 6.9 Hz, 3H), 1.06 (s, 9H), 1.04 (d, *J* = 6.8 Hz, 3H), 0.97 (t, *J* = 7.9 Hz, 9H), 0.93 (d, *J* = 6.8 Hz, 3H), 0.90 – 0.84 (m, 5H), 0.69 – 0.58 (m, 6H).

Macrolide 38. In a 10-mL Schlenk tube under argon, a solution of *tert*-butyldimethylsilyl trifluoromethanesulfonate in dichloromethane (0.088 M, 0.20 mL, 18 µmol) was added dropwise to a solution of alcohol **35** (20.9 mg, 33.4 µmol) and trichloroacetimidate **39b** (39.0 mg, 103 µmol)[2] in a mixture of dichloromethane (1.5 mL) and acetonitrile (1.5 mL) at room temperature. After stirring for 1 h, an additional amount of the silyl triflate stock solution (0.57 mL, 50 µmol) was added, and stirring was continued for another 2 h. The mixture was diluted with ethyl acetate (10 mL) and washed with saturated aqueous sodium bicarbonate solution (10 mL). The layers were separated and the aqueous phase was extracted with ethyl acetate (5 × 10 mL). The combined organic layers were dried over anhydrous sodium sulfate, the drying agent was filtered off, and the solvent was removed under reduced pressure. Purification of the residue by flash chromatography with hexanes/acetone (4:1 → 3:1 → 2:1, 0.1% *v*/*v* of triethylamine added) furnished title compound as a colorless gum (9.2 mg, 33% yield). $[\alpha]_D^{20}$ = +28.5 (*c* 0.20, $CHCl_3$). ^{1}H NMR (400 MHz, $CDCl_3$): δ 8.16 – 8.01 (m, 2H), 7.71 – 7.62 (m, 1H), 7.57 – 7.47 (m, 2H), 7.06 (dd, *J* = 15.1, 10.3 Hz, 1H), 6.46 (dd, *J* = 15.6, 9.8 Hz, 1H), 6.11 (d, *J* = 15.0 Hz, 1H), 6.06 – 5.89 (m, 2H), 5.57 (d, *J* = 15.5 Hz, 1H), 5.39 – 5.29 (br m, 1H), 4.84 (td, *J* = 9.9, 2.6 Hz, 1H), 4.61 (d, *J* = 8.0 Hz, 1H), 4.59 – 4.52 (br m, 1H), 4.43 (dd, *J* = 9.9, 2.6 Hz, 1H), 4.00 (dd, *J* = 9.5, 3.8 Hz, 1H), 3.95 – 3.85 (m, 2H), 3.80 – 3.61 (br m, 2H), 3.55 – 3.45 (m, 7H), 3.26 (d, *J* = 10.0 Hz, 1H), 3.02 (dd, *J* = 8.0, 2.8 Hz, 1H), 2.81 (br s, 6H), 2.59 – 2.42 (m, 2H), 2.40 – 2.29 (m, 1H), 2.11 (s, 3H), 1.88 – 1.63 (m, 4H), 1.58 – 1.44 (m, 3H), 1.32 (d,

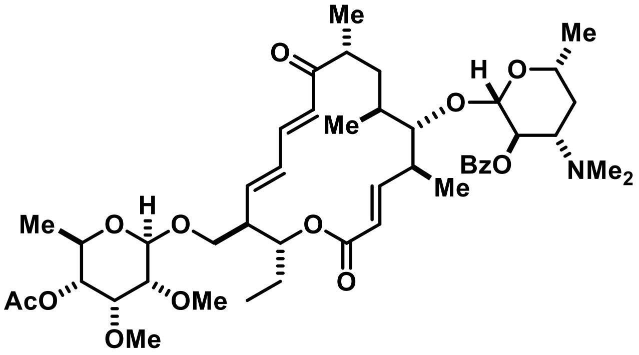

J = 6.1 Hz, 3H), 1.16 (d, J = 6.2 Hz, 3H), 1.13 (d, J = 7.0 Hz, 3H), 0.94 (d, J = 6.8 Hz, 3H), 0.91 – 0.84 (m, 6H). $^{13}C\{^1H\}$ NMR (101 MHz, $CDCl_3$): δ 203.6, 170.3, 166.0, 165.2, 150.5, 142.0, 141.6, 134.4, 133.1, 130.2, 129.1, 128.6, 123.4, 121.4, 101.5, 101.1, 87.3, 80.7, 77.9, 74.9, 74.0, 69.7, 68.8, 68.4, 67.5, 64.4, 61.7, 59.8, 49.3, 44.8, 44.5, 40.8, 37.2, 34.4, 34.1, 32.3, 25.3, 21.1, 20.6, 19.5, 17.9, 17.5, 17.4, 9.8. IR (film): 3373, 2968, 2933, 2880, 1726, 1679, 1653, 1595, 1453, 1373, 1325, 1262, 1236, 1170, 1090, 1067, 1001, 984, 754, 713 cm^{-1}. HRMS-ESI m/z: $[M+H]^+$ calcd for $C_{46}H_{68}NO_{13}$ 842.4685; found 842.4684.

Analytical and spectral data of the α-anomer isolated in an experiment using fluoride **39a** *as the glycosyl donor*: $[\alpha]_D^{20}$ = +75.9 (c 0.27, $CHCl_3$). ^1H NMR (400 MHz, $CDCl_3$): δ 8.10 – 8.03 (m, 2H), 7.62 – 7.55 (m, 1H), 7.51 – 7.42 (m, 2H), 7.10 (dd, J = 15.0, 10.9 Hz, 1H), 6.49 (dd, J = 15.5, 10.0 Hz, 1H), 6.15 (d, J = 15.1 Hz, 1H), 6.06 (dd, J = 15.3, 10.9 Hz, 1H), 5.92 (dd, J = 15.3, 9.1 Hz, 1H), 5.60 (d, J = 15.5 Hz, 1H), 5.25 – 5.10 (br m, 1H), 4.87 – 4.79 (m, 1H), 4.81 (d, J = 4.0 Hz, 1H), 4.49 (d, J = 7.4 Hz, 1H), 4.45 (dd, J = 10.0, 2.7 Hz, 1H), 4.19 – 4.09 (m, 1H), 3.94 (t, J = 2.8 Hz, 1H), 3.77 (dd, J = 9.6, 7.0 Hz, 1H), 3.62 – 3.48 (m, 2H), 3.48 (s, 3H), 3.40 (s, 3H), 3.30 (dd, J = 4.2, 2.9 Hz, 1H), 3.24 (d, J = 9.9 Hz, 1H), 3.19 – 3.01 (br m, 1H), 2.65 – 2.34 (m, 9H), 2.12 (s, 3H), 1.87 – 1.74 (m, 1H), 1.73 – 1.38 (m, 3H), 1.32 – 1.19 (m, 3H), 1.29 (d, J = 6.1 Hz, 3H), 1.14 (d, J = 7.0 Hz, 3H), 1.13 (d, J = 6.3 Hz, 3H), 0.98 (d, J = 6.9 Hz, 3H), 0.92 (d, J = 6.8 Hz, 3H), 0.89 (t, J = 7.3 Hz, 3H). $^{13}C\{^1H\}$ NMR (101 MHz, $CDCl_3$): δ 204.0, 170.5, 166.1, 165.4, 150.9 (br), 142.2, 141.8, 133.5, 133.2, 130.0, 128.7, 123.2, 121.3, 102.4 (br), 97.1, 87.6, 78.7, 75.2, 74.5, 74.1, 71.3 (br), 68.9 (br), 68.3, 64.1, 61.8, 61.0, 57.4, 48.8, 44.9, 40.9, 34.2, 32.3, 25.4, 21.2, 20.9, 19.4, 17.9, 17.5, 17.1, 9.8.. IR (film): 3407, 2971, 2934, 2879, 1723, 1680, 1652, 1632, 1594, 1454, 1375, 1354, 1323, 1266, 1236, 1177, 1108, 1051, 1001, 984, 752, 712, 665 cm^{-1}. HRMS-ESI m/z: $[M+H]^+$ calcd for $C_{46}H_{68}NO_{13}$ 842.4685; found 842.4684.

Mycinamicin IV (1). In a 10-mL Schlenk tube equipped with a cooling finger under argon,

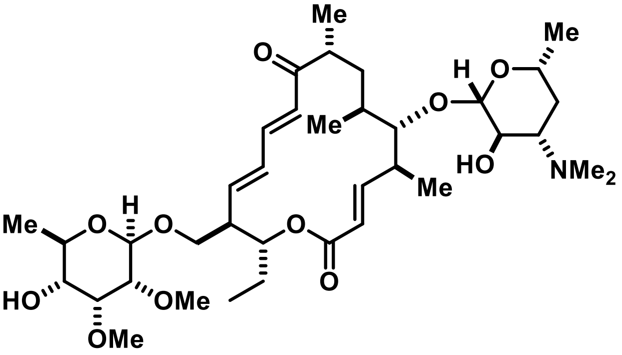

macrolide **38** (9.2 mg, 11 µmol) was treated with a mixture of methanol, triethylamine and water (5:1:1 $v/v/v$, 0.80 mL) and the resulting mixture was stirred at 70 °C (bath temperature) for 6.5 h. For work-up, the mixture was cooled to room temperature, diluted with ethyl acetate (10 mL) and washed with saturated aqueous sodium carbonate solution (10 mL). The layers were separated, and the aqueous phase was extracted with ethyl acetate (5 × 10 mL). The combined organic layers were dried over anhydrous sodium sulfate, the drying agent was filtered off, and the solvent was removed under reduced pressure. Purification of the residue by flash chromatography with dichloromethane/methanol (9:1, 0.1% v/v of triethylamine added) furnished the

title compound as a white, amorphous solid containing trace impurities (5.4 mg, 71% yield). Analytically pure Mycinamicin IV was obtained by preparative HPLC: Agilent 1260 Infinity pump, 150 mm length × 10 mm diameter YMC Triart C18 5 µm column, methanol/aq. 20 mM NH_4HCO_3 pH 9.0 (70:30 *v*/*v*, 4.7 mL/min, 10.5 MPa, 300 K) eluent, UV-detection at 210 nm.

$[\alpha]_D^{25}$ = +7.5 (*c* 0.3, $CHCl_3$). ^{1}H NMR (400 MHz, C_6D_6): δ 7.44 – 7.34 (m, 1H, 11-H), 6.85 (dd, *J* = 15.5, 10.0 Hz, 1H, 3-H), 6.13 (d, *J* = 15.0 Hz, 1H, 10-H), 6.01 – 5.94 (m, 2H, 12-H, 13-H), 5.62 (d, *J* = 15.5 Hz, 1H, 2-H), 5.17 – 5.09 (m, 1H, 15-H), 4.66 (d, *J* = 7.8 Hz, 1H, 1''-H), 4.14 (d, *J* = 7.2 Hz, 1H, 1'-H), 3.92 (dd, *J* = 9.5, 3.7 Hz, 1H, 21a-H), 3.60 – 3.49 (m, 1H, 5''-H), 3.41 – 3.32 (m, 8H, 7''-H_3, 8''-H_3, 2'-H, 3''-H), 3.17 (dd, *J* = 9.5, 6.1 Hz, 1H, 21b-H), 3.11 (d, *J* = 10.3 Hz, 1H, 5-H), 3.07 – 2.98 (m, 2H, 5'-H, 4''-H), 2.84 (dd, *J* = 7.9, 2.8 Hz, 1H, 2''-H), 2.84 – 2.66 (m, 2H, 8-H, 4-H), 2.43 – 2.32 (m, 1H, 14-H), 2.24 – 2.15 (m, 1H, 3'-H), 1.86 (s, 6H, 7'-H_6), 1.85 – 1.75 (m, 1H, 7a-H), 1.72 – 1.59 (m, 2H, 7b-H, 16a-H), 1.52 – 1.43 (m, 1H, 6-H), 1.41 – 1.34 (m, 7H, 6''-H_3, 16b-H, 18-H_3), 1.28 (d, *J* = 6.8 Hz, 3H, 19-H_3), 1.13 – 1.00 (m, 7H, 4'a-H, 6'-H_3, 20-H_3), 0.94 (t, *J* = 7.3 Hz, 3H, 17-H_3), 0.93 – 0.80 (m, 1H, 4'b-H). ^{13}C{^{1}H} NMR (101 MHz, C_6D_6): δ 201.9 (9-C), 166.1 (1-C), 152.1 (3-C), 141.6 (11-C), 141.3 (13-C), 133.6 (12-C), 124.1 (10-C), 121.6 (2-C), 105.4 (1'-C), 101.7 (1''-C), 88.0 (5-C), 82.6 (2''-C), 80.7 (3''-C), 73.7 (15-C), 73.0 (4''-C), 70.9 (5''-C), 70.6 (2'-C), 69.2 (5'-C), 68.6 (21-C), 66.4 (3'-C), 61.6 (8''-C), 59.7 (7''-C), 49.5 (14-C), 45.3 (8-C), 41.6 (4-C), 39.9 (7'-C), 34.7 (6-C), 33.1 (7-C), 28.5 (4'-C), 25.5 (16-C), 21.3 (6'-C), 19.7 (18-C), 18.3 (6''-C), 17.9 (19-C), 17.8 (20-C), 10.0 (17-C). ^{1}H NMR (600 MHz, $CDCl_3$): δ 7.11 (ddd, *J* = 15.1, 11.0, 0.7 Hz, 1H, 11-H), 6.61 (dd, *J* = 15.5, 9.9 Hz, 1H, 3-H), 6.21 (d, *J* = 15.0 Hz, 1H, 10-H), 6.12 (ddd, *J* = 15.3, 11.0, 0.7 Hz, 1H, 12-H), 5.99 (dd, *J* = 15.3, 9.2 Hz, 1H, 13-H), 5.76 (dd, *J* = 15.4, 0.7 Hz, 1H, 2-H), 4.89 (ddd, *J* = 10.2, 9.0, 2.7 Hz, 1H, 15-H), 4.57 (d, *J* = 7.8 Hz, 1H, 1''-H), 4.24 (d, *J* = 7.3 Hz, 1H, 1'-H), 4.04 (dd, *J* = 9.5, 3.8 Hz, 1H, 21a-H), 3.75 (t, *J* = 3.1 Hz, 1H, 3''-H), 3.62 (s, 3H, 8''-H_3), 3.54 (dd, *J* = 9.5, 6.7 Hz, 1H, 21b-H), 3.53 – 3.40 (m, 7H, 5''-H, 7''-H_3, 5'-H, 2'-OH, 4''-OH), 3.28 (d, *J* = 10.5 Hz, 1H, 5-H), 3.24 (dd, *J* = 10.2, 7.3 Hz, 1H, 2'-H), 3.21 – 3.15 (br m, 1H, 4''-H), 3.03 (dd, *J* = 7.8, 2.8 Hz, 1H, 2''-H), 2.79 – 2.71 (m, 1H, 4-H), 2.59 – 2.50 (m, 2H, 8-H, 14-H), 2.47 (ddd, *J* = 12.3, 10.2, 3.9 Hz, 1H, 3'-H), 2.27 (s, 6H, 7'-H_6), 1.88 – 1.80 (m, 1H, 16a-H), 1.67 – 1.52 (m, 4H, 4'a-H, 16b-H, 7-H_2), 1.29 – 1.22 (m, 2H, 4'b-H, 6-H), 1.27 (d, *J* = 6.3 Hz, 3H, 6''-H_3), 1.24 (d, *J* = 6.8 Hz, 3H, 18-H_3), 1.23 (d, *J* = 6.1 Hz, 3H, 6'-H_3), 1.13 (d, *J* = 6.9 Hz, 3H, 20-H_3), 0.99 (d, *J* = 6.9 Hz, 3H, 19-H_3), 0.94 (t, *J* = 7.3 Hz, 3H, 17-H_3). ^{13}C{^{1}H} NMR (151 MHz, $CDCl_3$): δ 203.8 (9-C), 166.2 (1-C), 151.8 (3-C), 141.8 (11-C), 141.4 (13-C), 133.1 (12-C), 123.2 (10-C), 120.9 (2-C), 105.0 (1'-C), 101.1 (1''-C), 87.9 (5-C), 81.9 (2''-C), 79.9 (3''-C), 73.7 (15-C), 72.7 (4''-C), 70.6 (5''-C), 70.4 (2'-C), 69.5 (5'-C), 68.6 (21-C), 65.9 (3'-C), 61.8 (8''-C), 59.9 (7''-C), 49.2 (14-C), 44.9 (8-C), 41.4 (4-C), 40.3 (7'-C), 34.1 (6-C), 32.6 (7-C), 28.2 (4'-C), 25.3 (16-C), 21.2 (6'-C), 19.4 (18-C), 17.8 (6''-C), 17.7 (20-C), 17.4 (19-C), 9.7 (17-C). IR (film): 3453, 2968, 2932, 2878, 1714, 1679, 1651, 1593, 1458, 1379, 1353,

1324, 1277, 1233, 1167, 1073, 984, 963, 936, 835, 713, 546 cm^{-1}. HRMS-ESI *m/z*: $[M+H]^+$ calcd for $C_{37}H_{62}NO_{11}$ 696.4317; found 696.4310.

Table S2. Comparison of the NMR Data of Natural and Synthetic Mycinamicin IV (**1**): Signals of the aglycone (numbering as shown in the Insert; indiscernible signals are reported without designating any multiplicity)

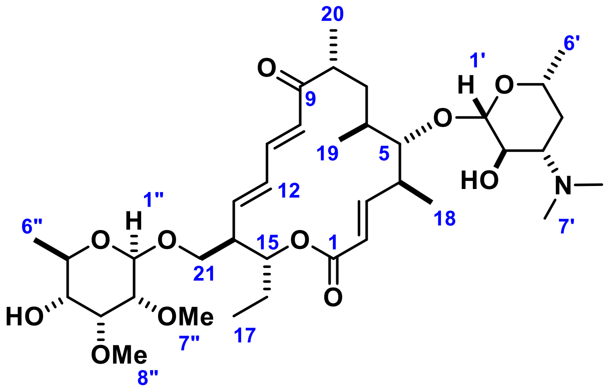

Position	δ_C natural	δ_C synthetic	$\Delta\delta_C$	δ_H (*J*/Hz) synthetic
1	166.1	166.2	+0.1	-
2	120.9	120.9	0.0	5.76 dd (15.4, 0.7)
3	151.6	151.8	+0.2	6.61 dd (15.5, 9.9)
4	41.3	41.4	+0.1	2.75
5	87.9	87.9	0.0	3.28 d (10.5)
6	34.1	34.1	0.0	1.26
7	32.6	32.6	0.0	1.63
				1.56
8	44.9	44.9	0.0	2.56
9	203.4	203.8	+0.4	-
10	123.2	123.2	0.0	6.21 d (15.0)
11	141.7	141.8	+0.1	7.11 ddd (15.1, 11.0, 0.7)
12	133.0	133.1	+0.1	6.12 ddd (15.3, 11.0, 0.7)
13	141.3	141.4	+0.1	5.99 dd (15.3, 9.2)
14	49.2	49.2	0.0	2.53
15	72.7	73.7	+1.0	4.89 ddd (10.2, 9.0, 2.7)
16	25.3	25.3	0.0	1.84
				1.56
17	9.6	9.7	+0.1	0.94 t (7.3)
18	19.4	19.4	0.0	1.24 d (6.8)
19	17.4	17.4	0.0	0.99 d (6.9)
20	17.8	17.7	-0.1	1.13 d (6.9)
21	68.6	68.6	0.0	4.04 dd (9.5, 3.8)
				3.54 dd (9.5, 6.7)

Table S3. Comparison of the NMR Data of Natural and Synthetic Mycinamicin IV (**1**): Carbohydrate Signals (numbering as shown in the Insert to Table S2; indiscernible signals reported without designating multiplicity)

Position	δ_C natural	δ_C synthetic	$\Delta\delta_C$	δ_H (*J*/Hz) synthetic
1’	104.9	105.0	+0.1	4.24 d (7.3)
2’	70.4	70.4	0.0	3.24 dd (10.2, 7.3)
3’	65.8	65.9	+0.1	2.47 ddd (12.3, 10.2, 3.9)
4’	28.3	28.2	-0.1	1.65
				1.26
5’	69.5	69.5	0.0	3.49
6’	21.2	21.2	0.0	1.23 d (6.1)
7’	40.2	40.3	+0.1	2.27
1”	101.0	101.1	+0.1	4.57 d (7.8)
2”	81.9	81.9	0.0	3.03 dd (7.8, 2.8)
3”	79.9	79.9	0.0	3.75 t (3.1)
4”	72.7	72.7	0.0	3.18
5”	70.5	70.6	+0.1	3.52
6”	17.8	17.8	0.0	1.27 d (6.3)
7”	59.7	59.9	+0.2	3.51
8”	61.7	61.8	+0.1	3.62

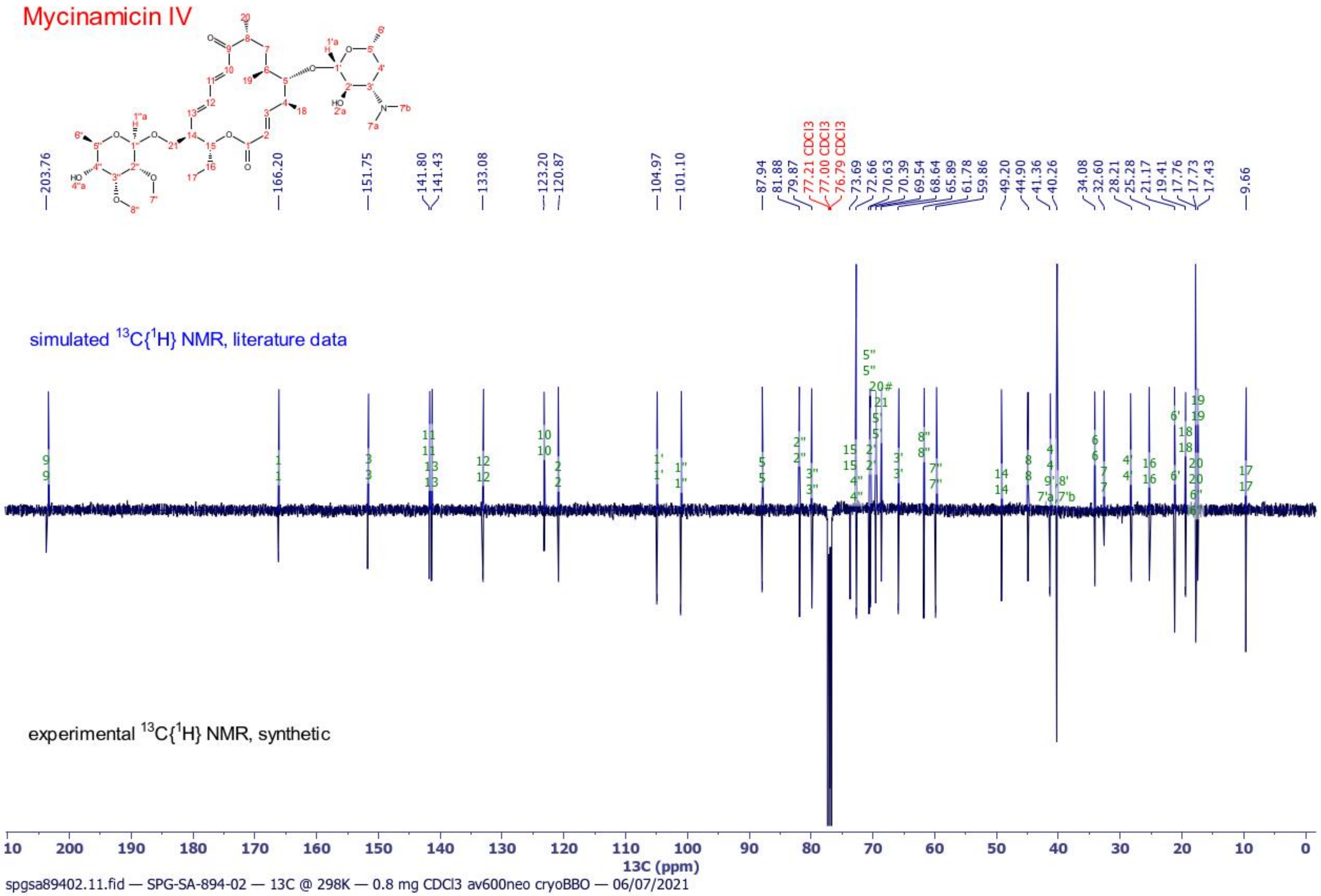

Fig. S1. Comparison of ^{13}C NMR data in $CDCl_3$ of natural sample (top, generated from tabulated data) and synthetic **1** (bottom)

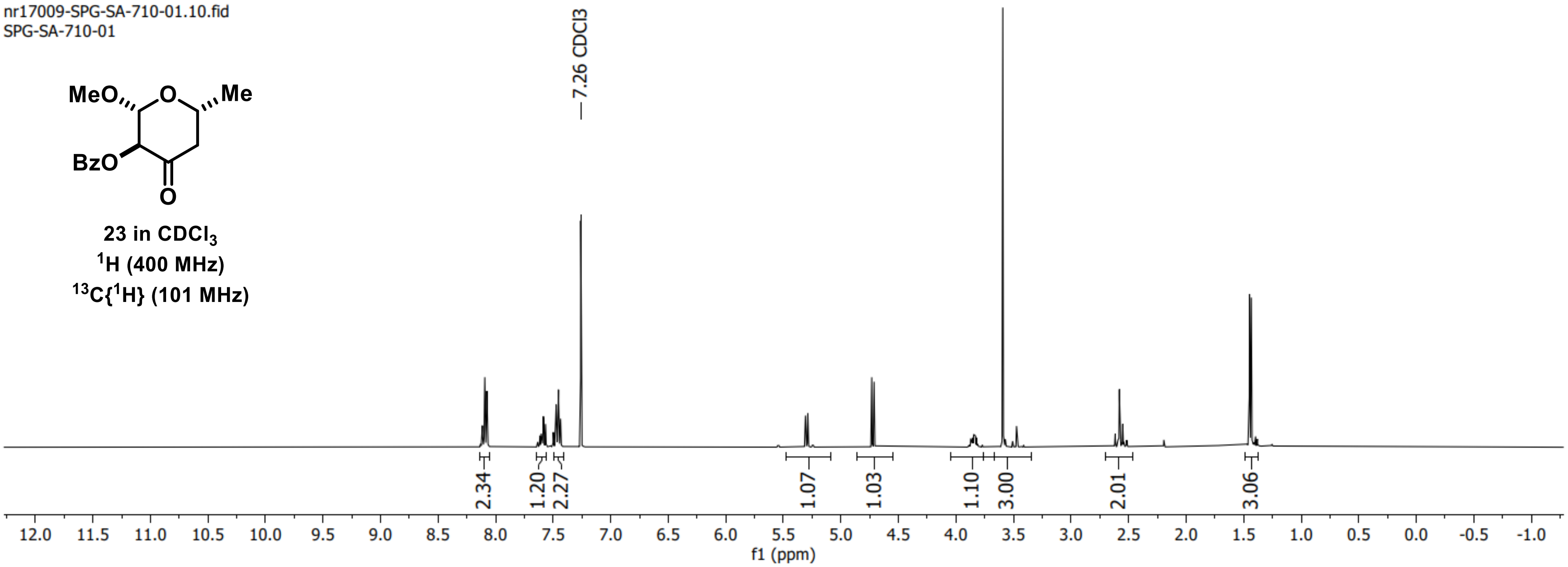

23 in $CDCl_3$
^1H (400 MHz)
$^{13}C\{^1H\}$ (101 MHz)

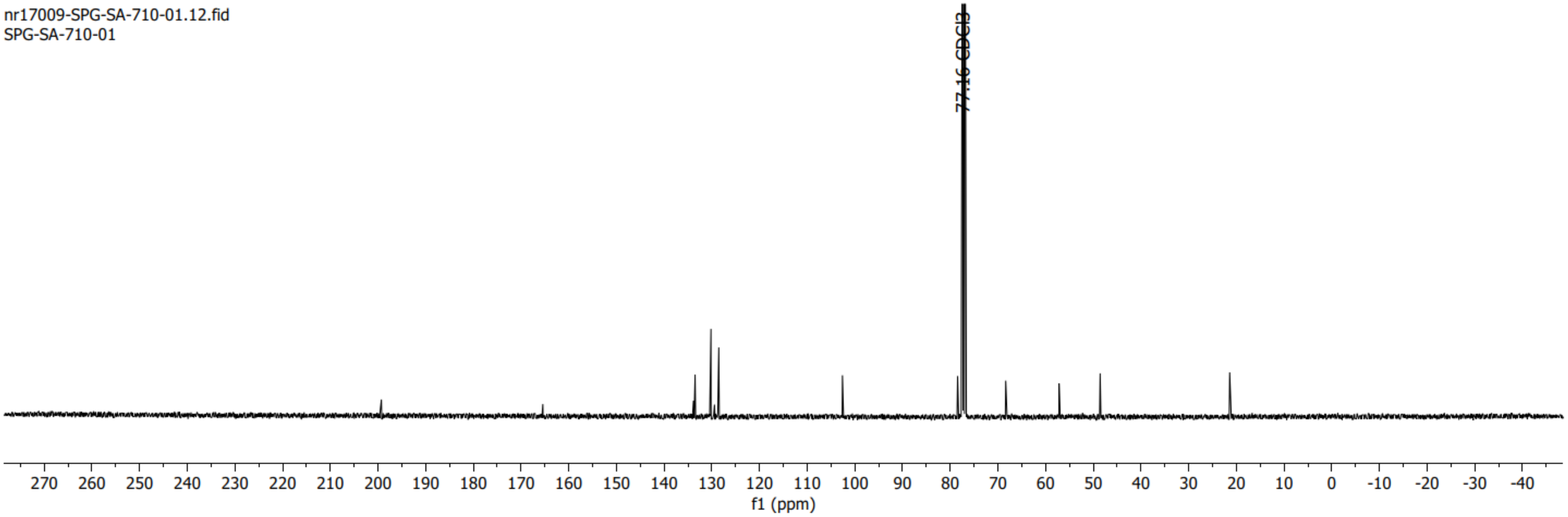

sr02016-SPG-SA-660-02.10.fid
SPG-SA-660-02

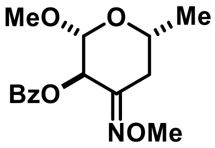

25 in $CDCl_3$
^1H (400 MHz)
$^{13}C\{^1H\}$ (101 MHz)

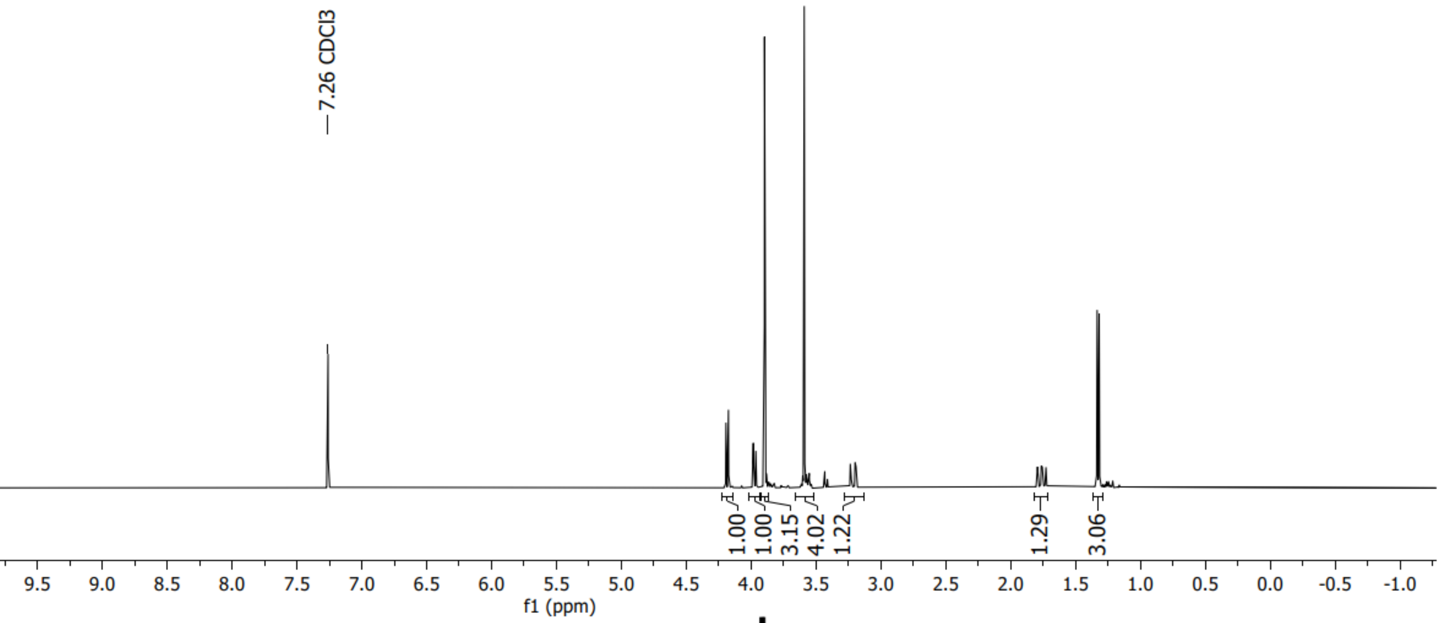

sr02016-SPG-SA-660-02.12.fid
SPG-SA-660-02

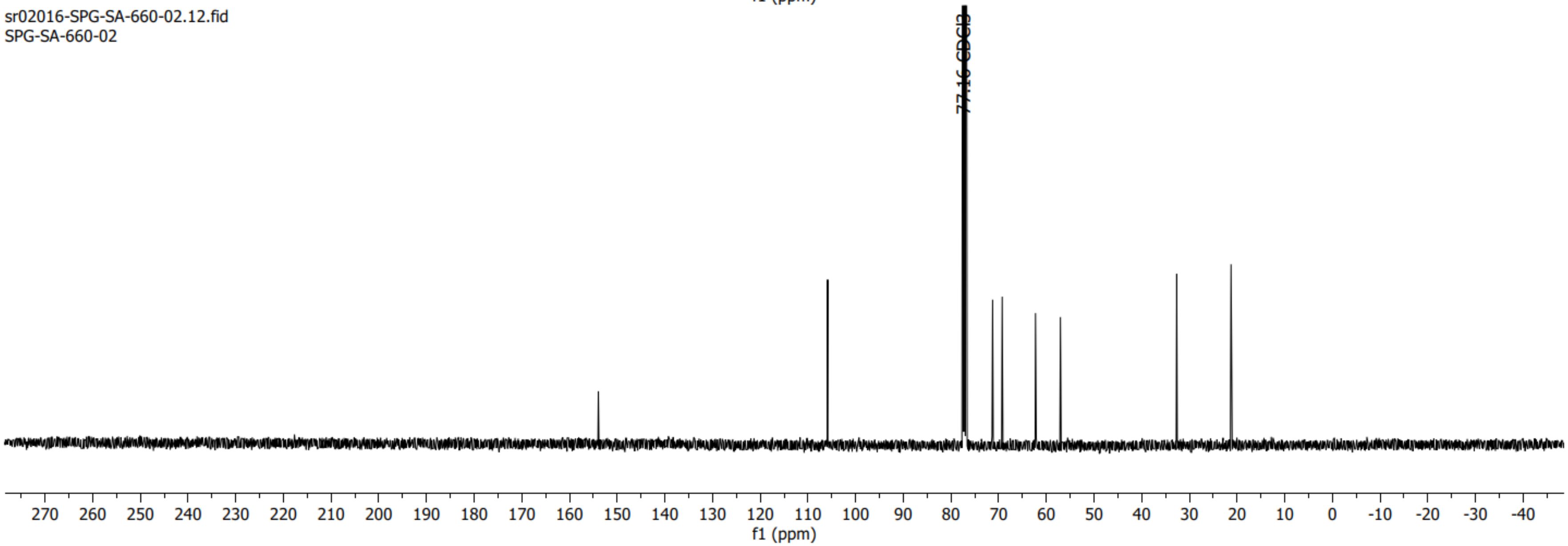

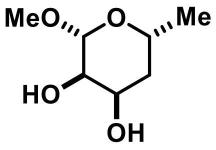

27 in $CDCl_3$
^{1}H (400 MHz)
$^{13}C\{^{1}H\}$ (101 MHz)

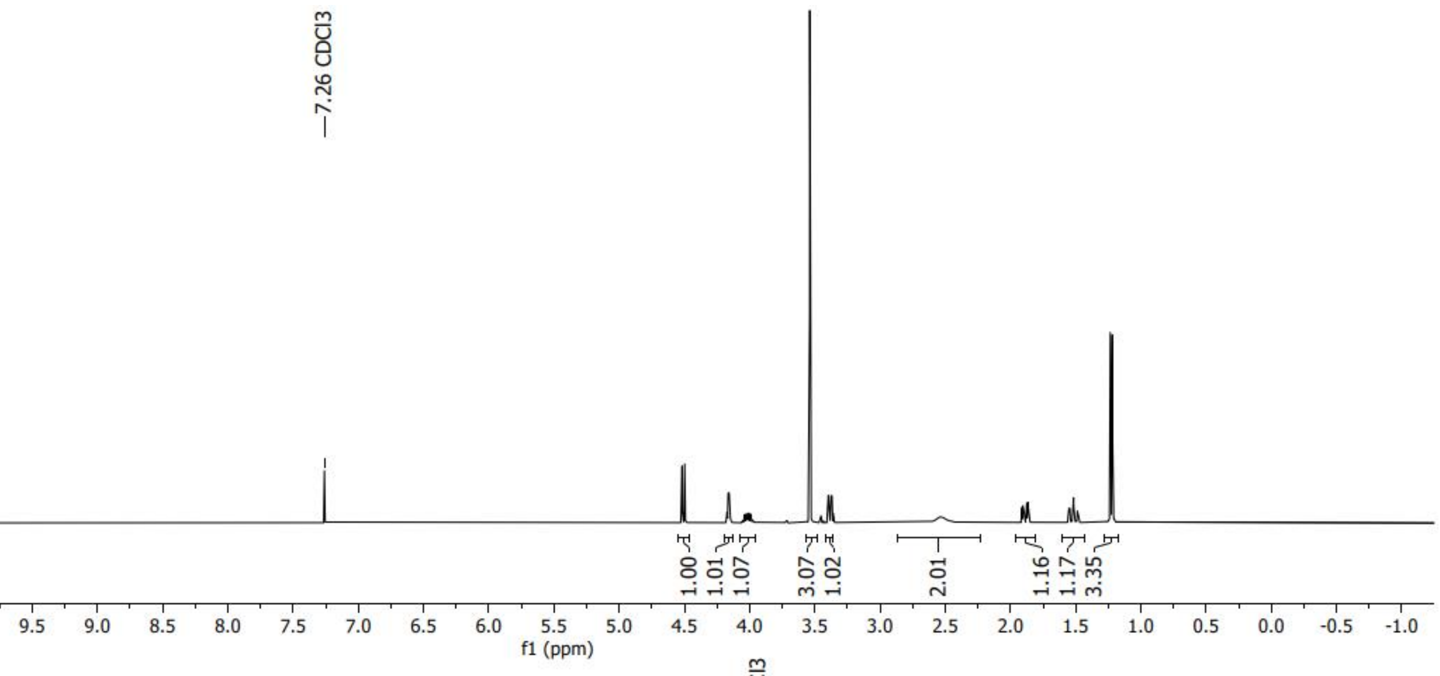

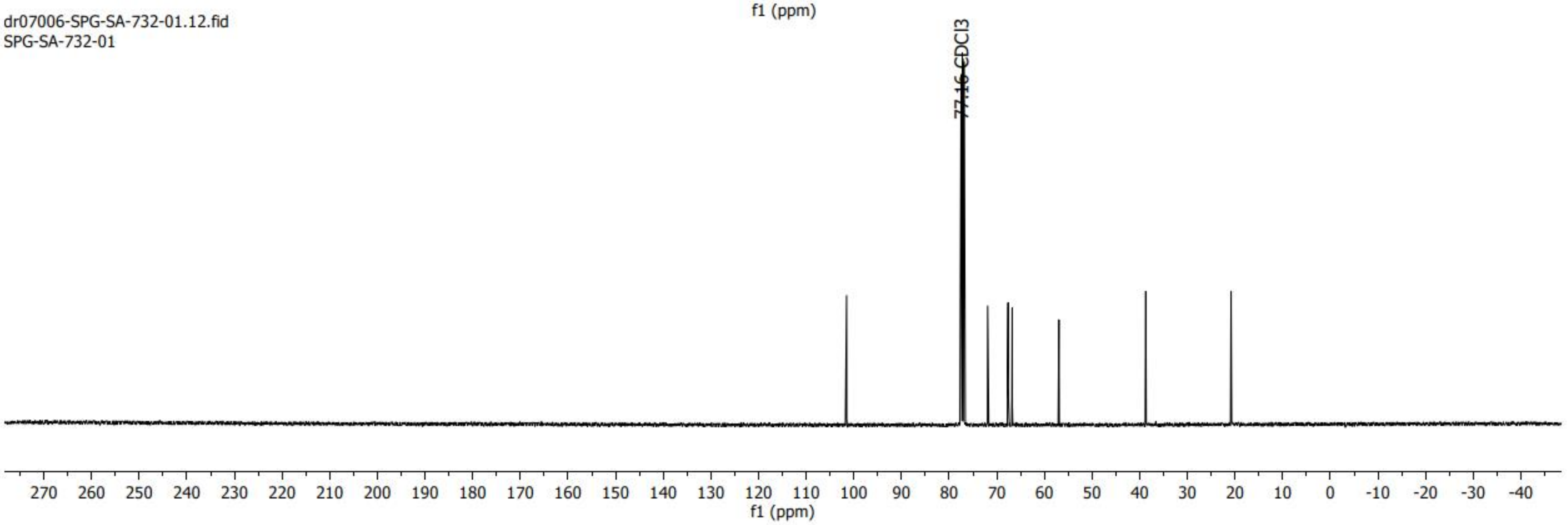

jr19074-SPG-SA-742-02.10.fid
SPG-SA-742-02

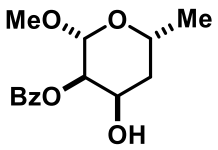

28 in $CDCl_3$
^1H (400 MHz)
$^{13}C\{^1H\}$ (101 MHz)

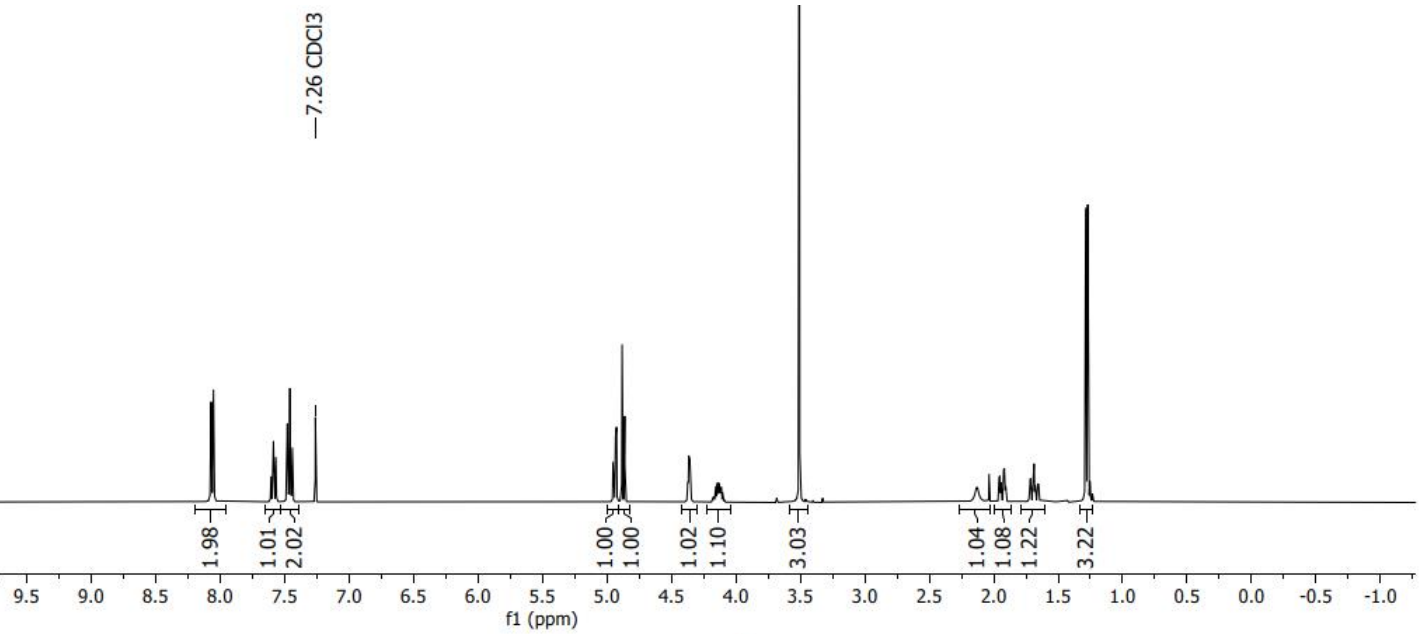

jr19074-SPG-SA-742-02.12.fid
SPG-SA-742-02

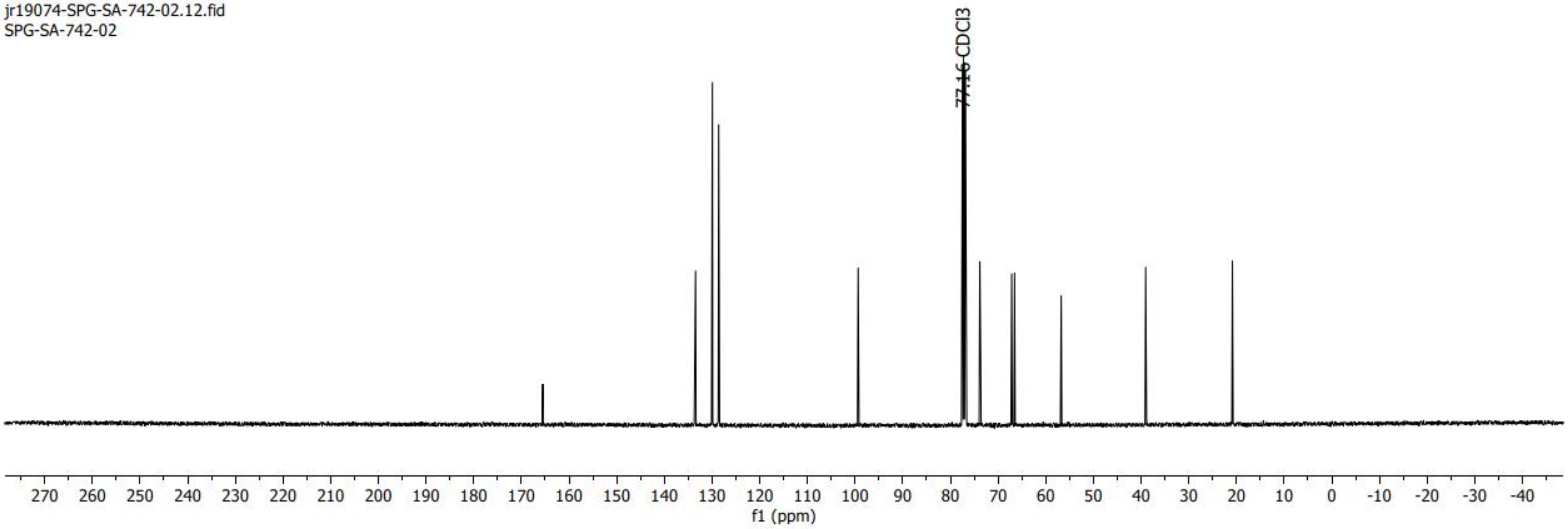

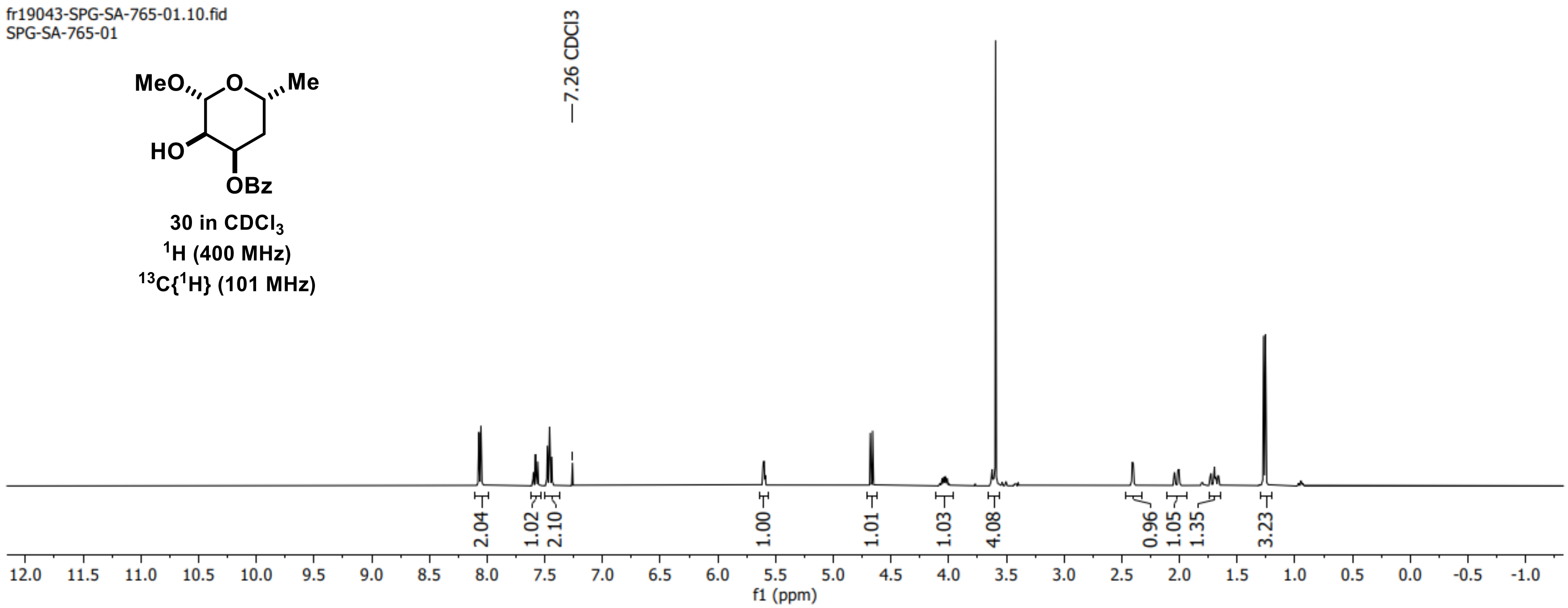
fr19043-SPG-SA-765-01.10.fid
SPG-SA-765-01
MeO
O
Me
HO
OBz
30 in $CDCl_3$
^1H (400 MHz)
$^{13}C\{^1H\}$ (101 MHz)
7.26 CDCl3
2.04
1.02
2.10
1.00
1.01
1.03
4.08
0.96
1.05
1.35
3.23
f1 (ppm)

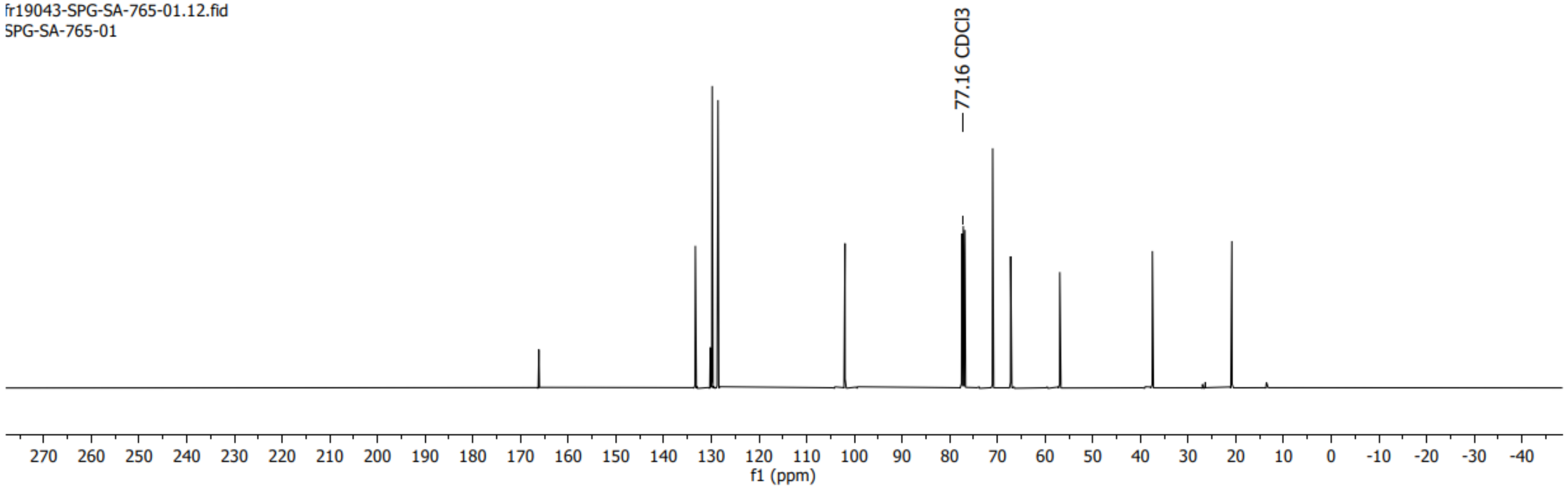
fr19043-SPG-SA-765-01.12.fid
SPG-SA-765-01
77.16 CDCl3
f1 (ppm)

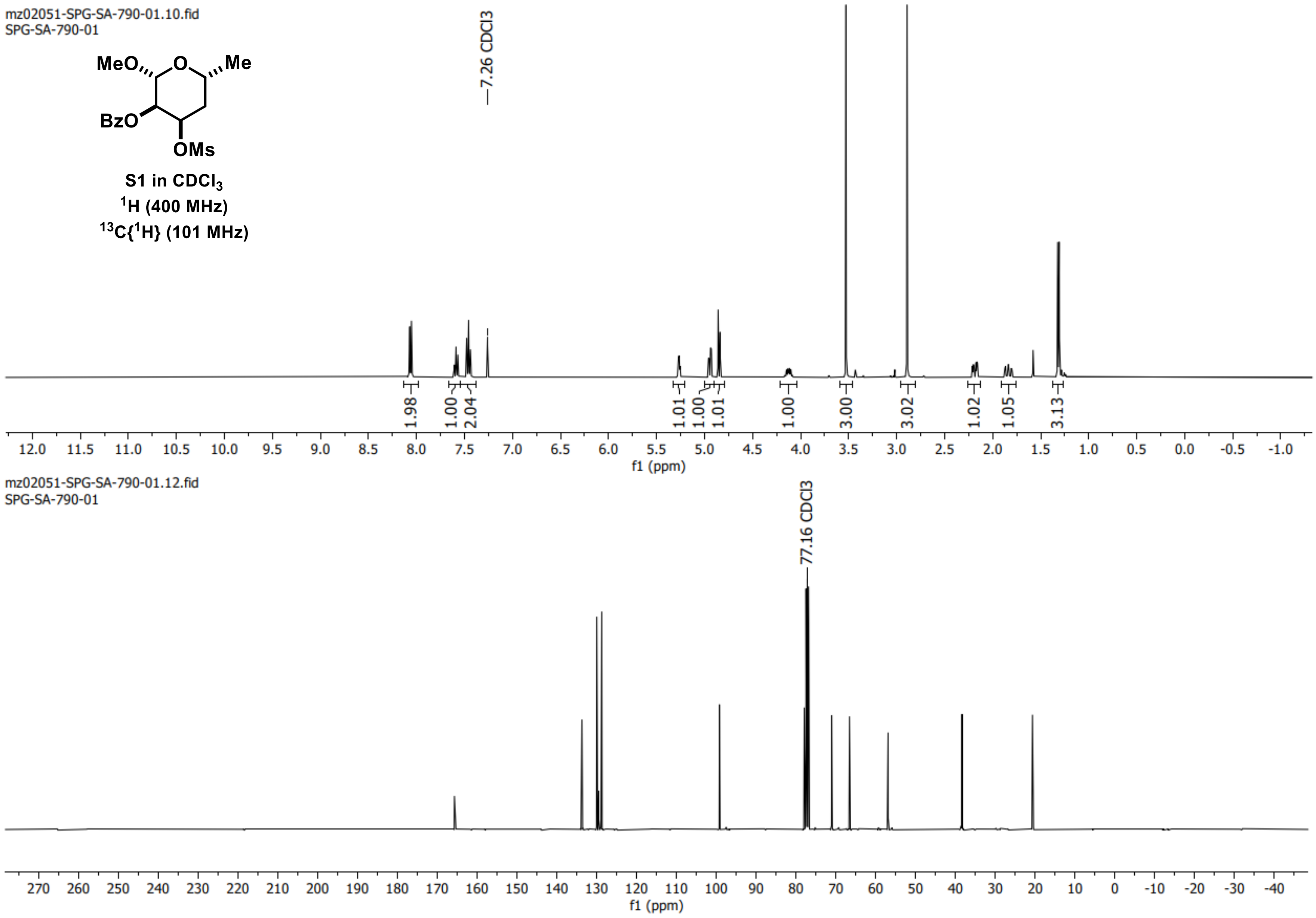
mz02051-SPG-SA-790-01.10.fid
SPG-SA-790-01
MeO
O
Me
BzO
OMs
S1 in CDCl3
1H (400 MHz)
13C{1H} (101 MHz)
7.26 CDCl3
1.98
1.00
2.04
1.01
1.00
1.01
1.00
3.00
3.02
1.02
1.05
3.13
f1 (ppm)
mz02051-SPG-SA-790-01.12.fid
SPG-SA-790-01
77.16 CDCl3
f1 (ppm)

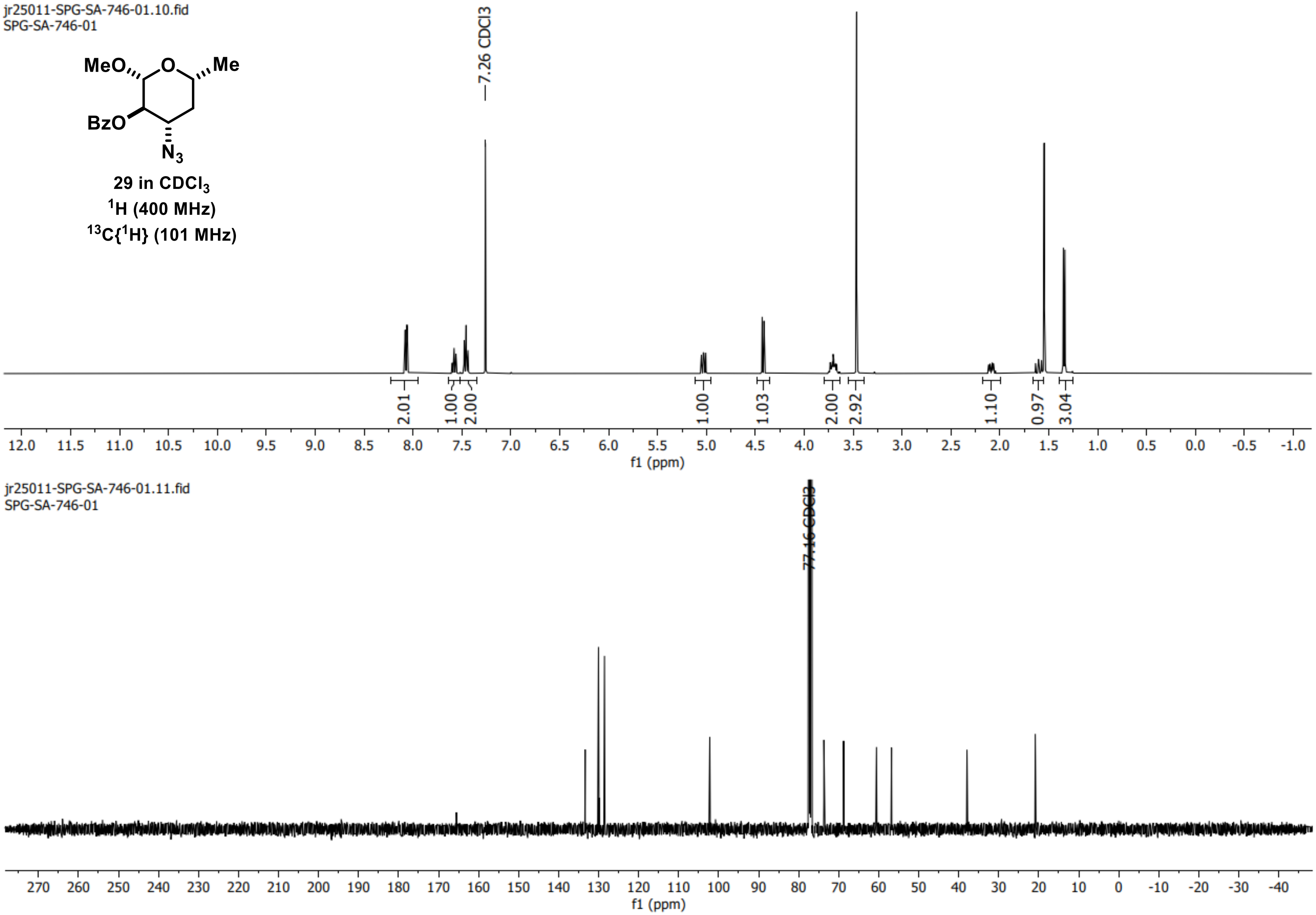

jr25011-SPG-SA-746-01.10.fid
SPG-SA-746-01
MeO
O
Me
BzO
N3
29 in CDCl3
1H (400 MHz)
13C{1H} (101 MHz)
7.26 CDCl3
2.01
1.00
2.00
1.00
1.03
2.00
2.92
1.10
0.97
3.04
f1 (ppm)
jr25011-SPG-SA-746-01.11.fid
SPG-SA-746-01
77.16 CDCl3
f1 (ppm)

mz04056-SPG-SA-794-02.10.fid
SPG-SA-794-02

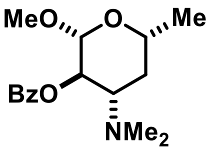

18c in $CDCl_3$
^{1}H (400 MHz)
$^{13}C\{^1H\}$ (101 MHz)

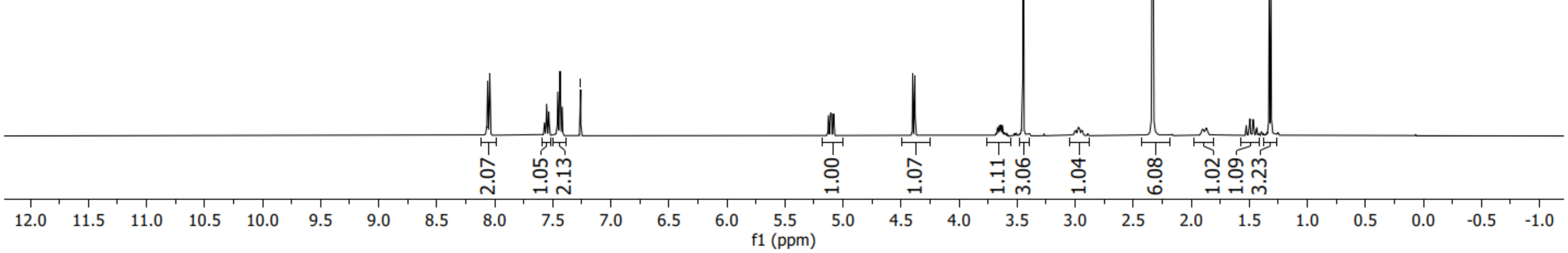

mz04056-SPG-SA-794-02.12.fid
SPG-SA-794-02

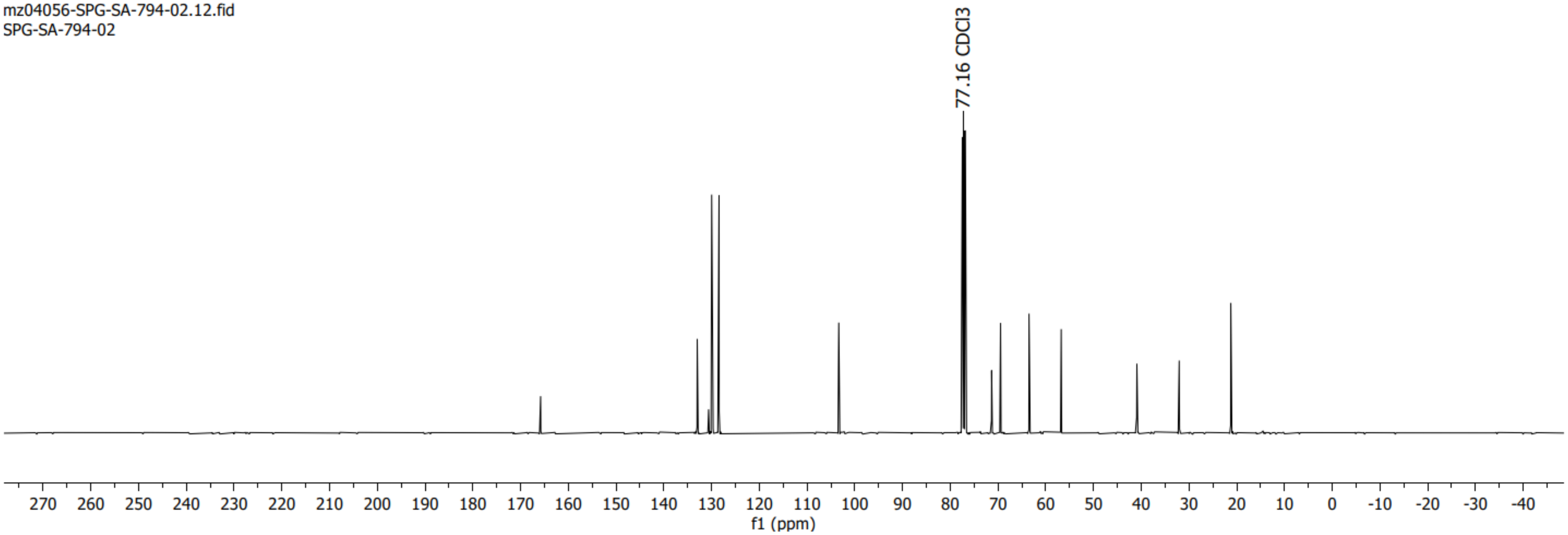

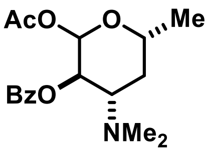

18d in $CDCl_3$
^{1}H (400 MHz)
^{13}C{^{1}H} (101 MHz)

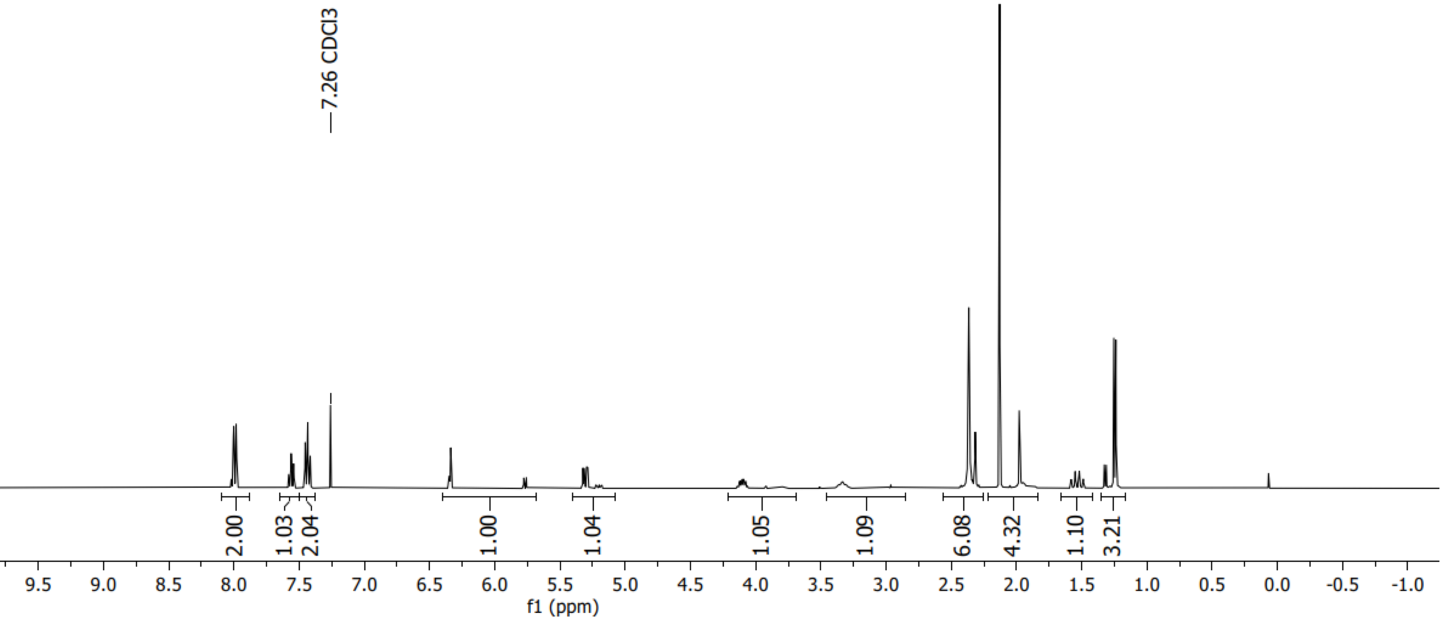

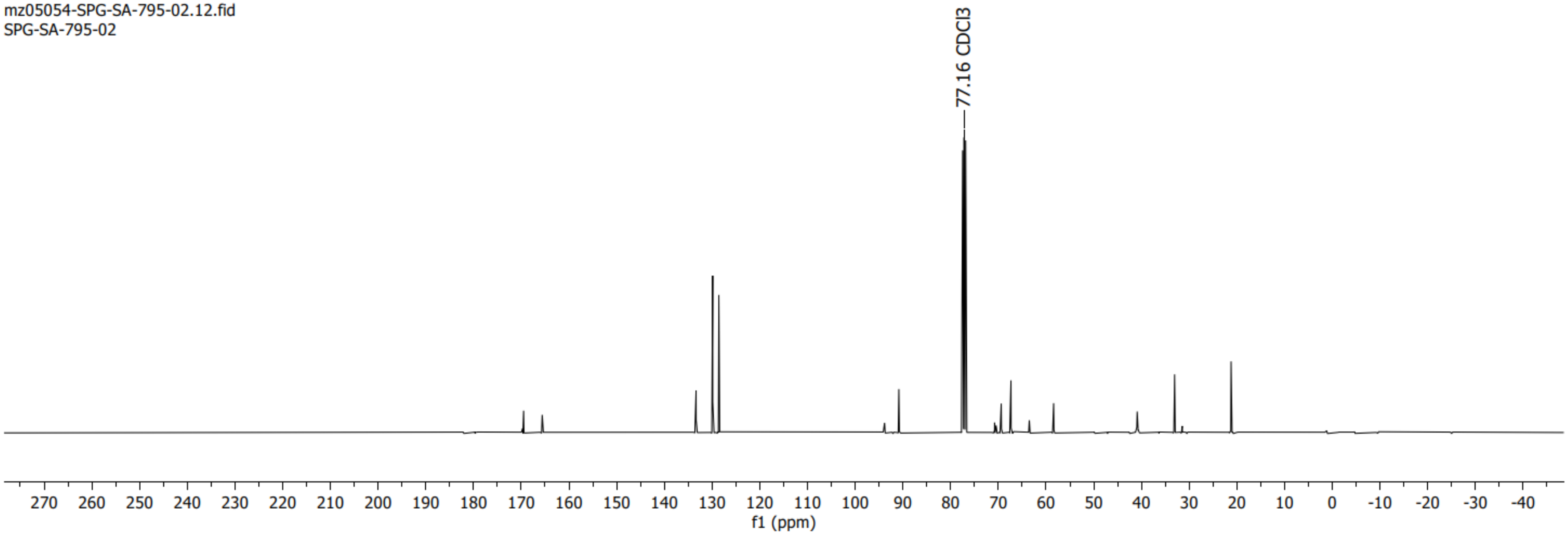

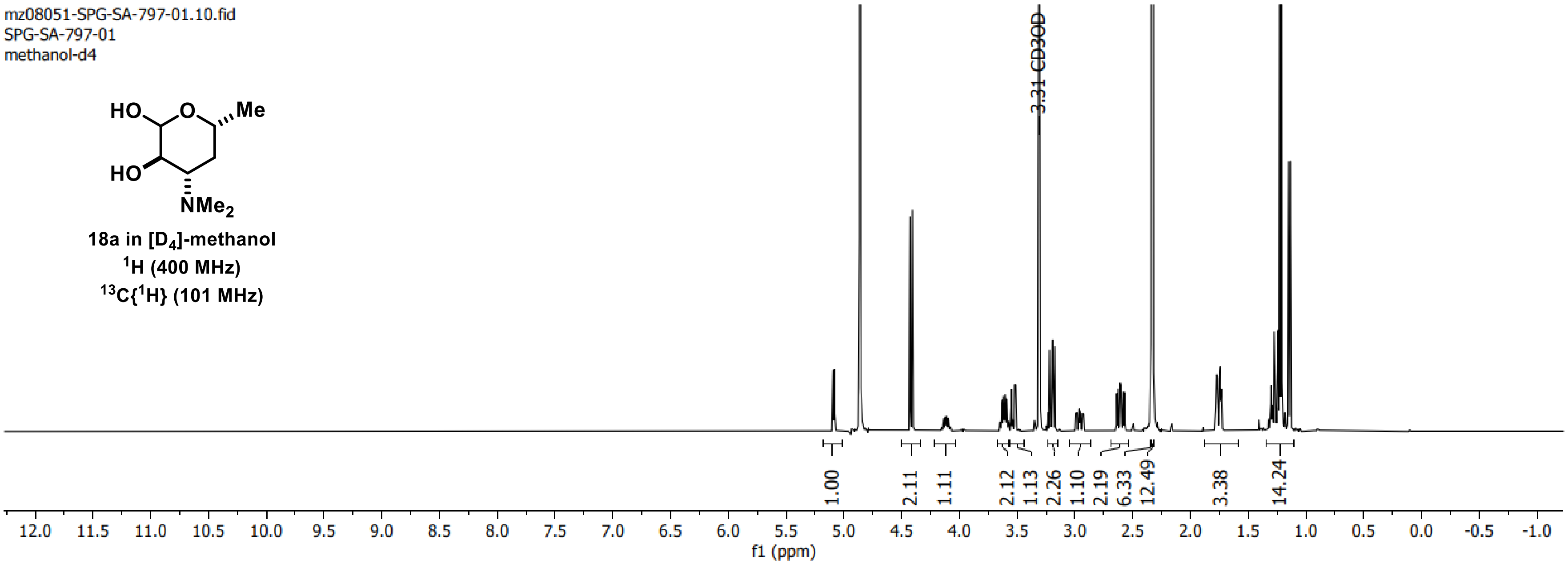
mz08051-SPG-SA-797-01.10.fid
SPG-SA-797-01
methanol-d4
HO
O
Me
HO
NMe2
18a in [D4]-methanol
1H (400 MHz)
13C{1H} (101 MHz)
3.31 CD3OD
1.00
2.11
1.11
2.12
1.13
2.26
1.10
2.19
6.33
12.49
3.38
14.24
12.0 11.5 11.0 10.5 10.0 9.5 9.0 8.5 8.0 7.5 7.0 6.5 6.0 5.5 5.0 4.5 4.0 3.5 3.0 2.5 2.0 1.5 1.0 0.5 0.0 -0.5 -1.0
f1 (ppm)

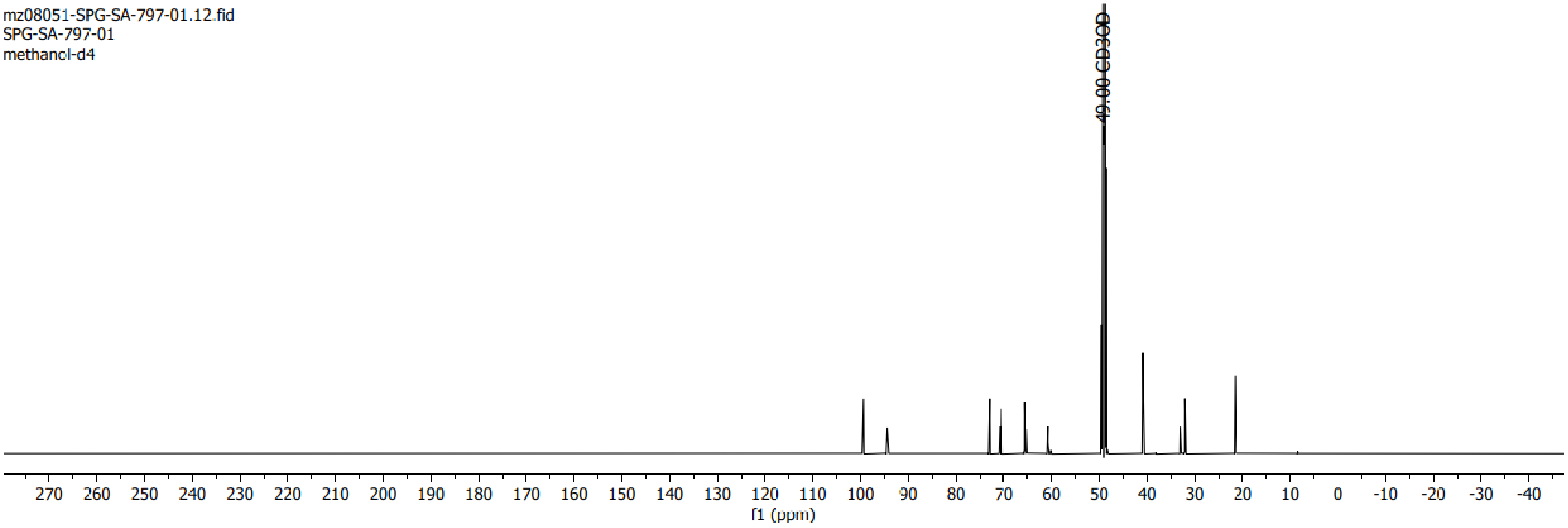
mz08051-SPG-SA-797-01.12.fid
SPG-SA-797-01
methanol-d4
49.00 CD3OD
270 260 250 240 230 220 210 200 190 180 170 160 150 140 130 120 110 100 90 80 70 60 50 40 30 20 10 0 -10 -20 -30 -40
f1 (ppm)

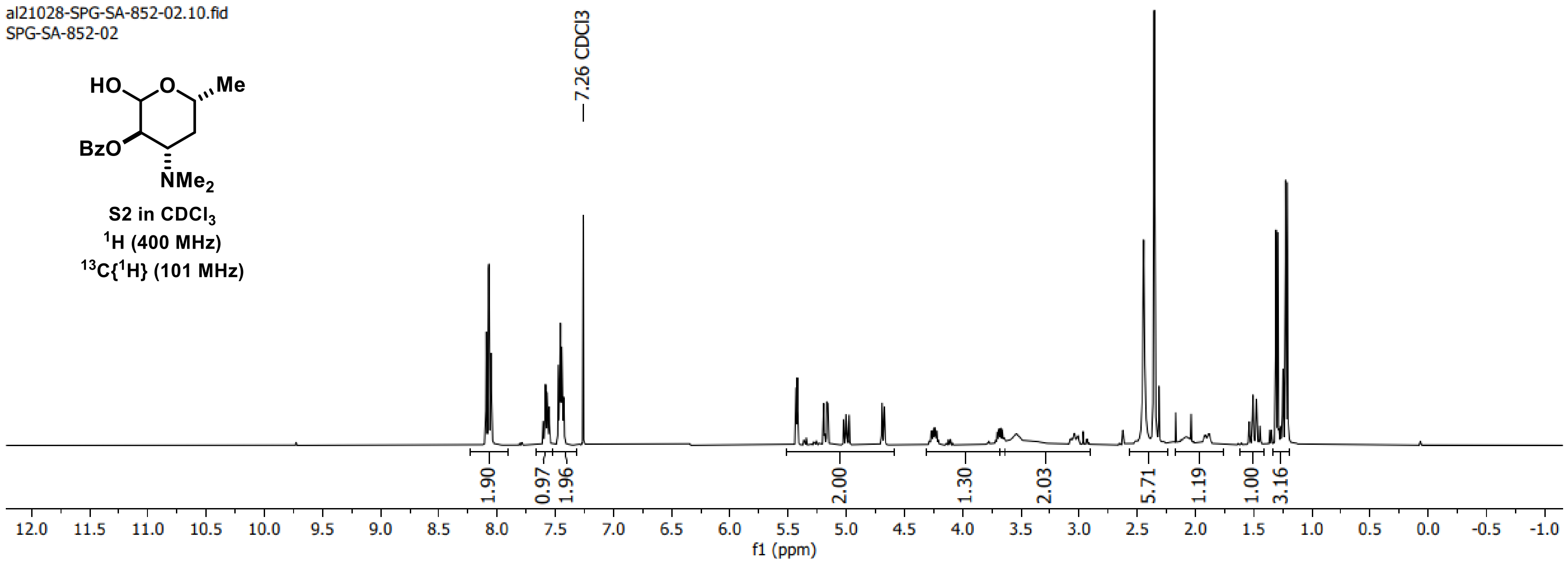
al21028-SPG-SA-852-02.10.fid
SPG-SA-852-02
HO
O
Me
BzO
NMe2
S2 in CDCl3
1H (400 MHz)
13C{1H} (101 MHz)
7.26 CDCl3
1.90
0.97
1.96
2.00
1.30
2.03
5.71
1.19
1.00
3.16
f1 (ppm)

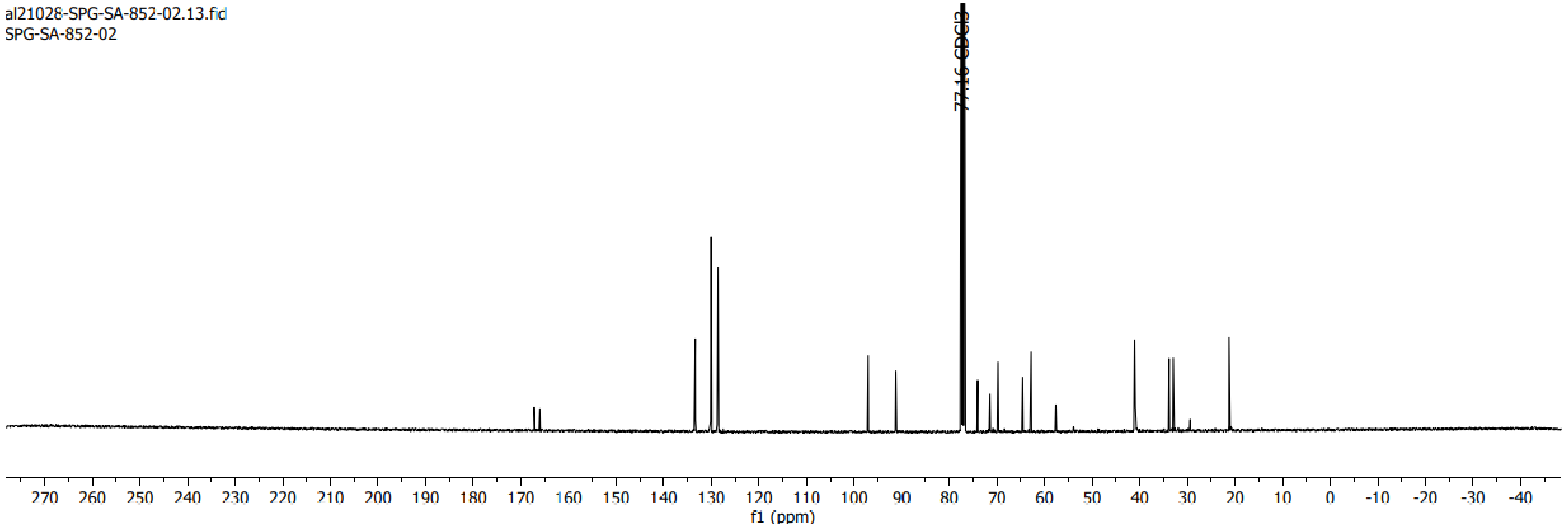
al21028-SPG-SA-852-02.13.fid
SPG-SA-852-02
77.16 CDCl3
f1 (ppm)

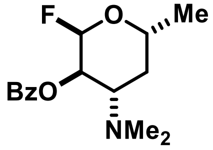

18e in $CDCl_3$
^{1}H (400 MHz)
^{13}C{^{1}H} (101 MHz)

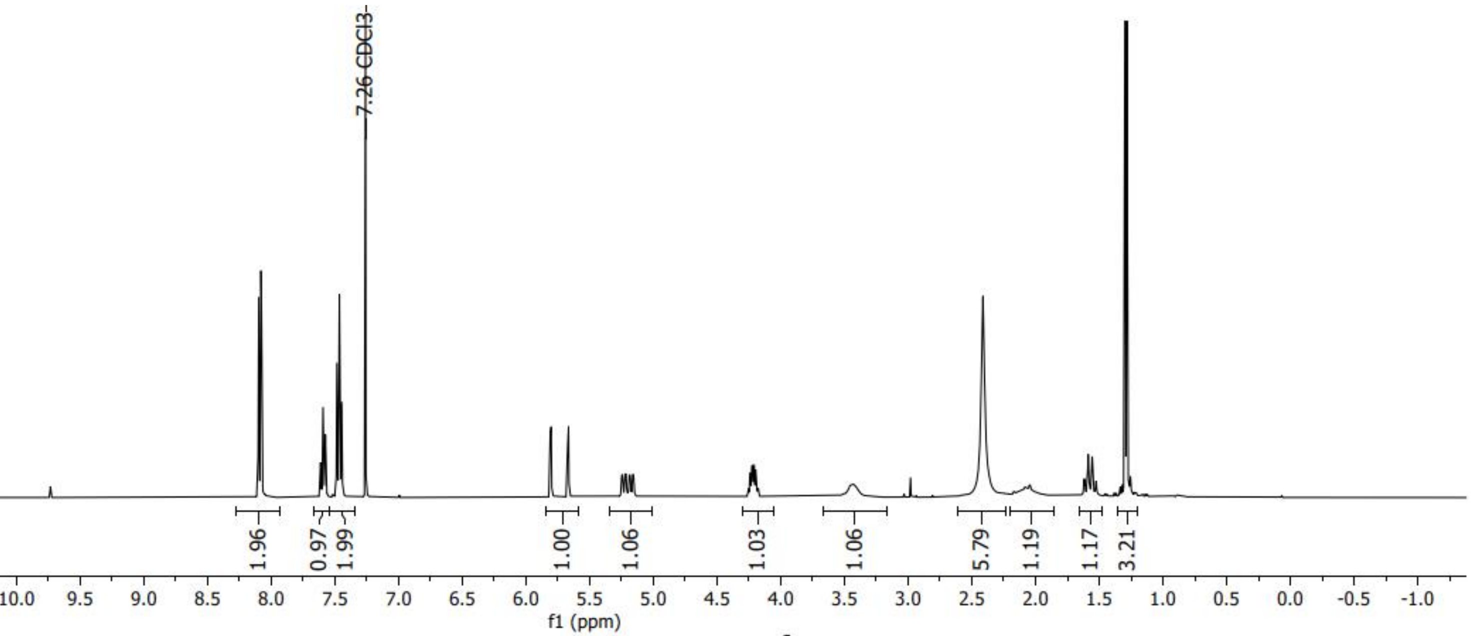

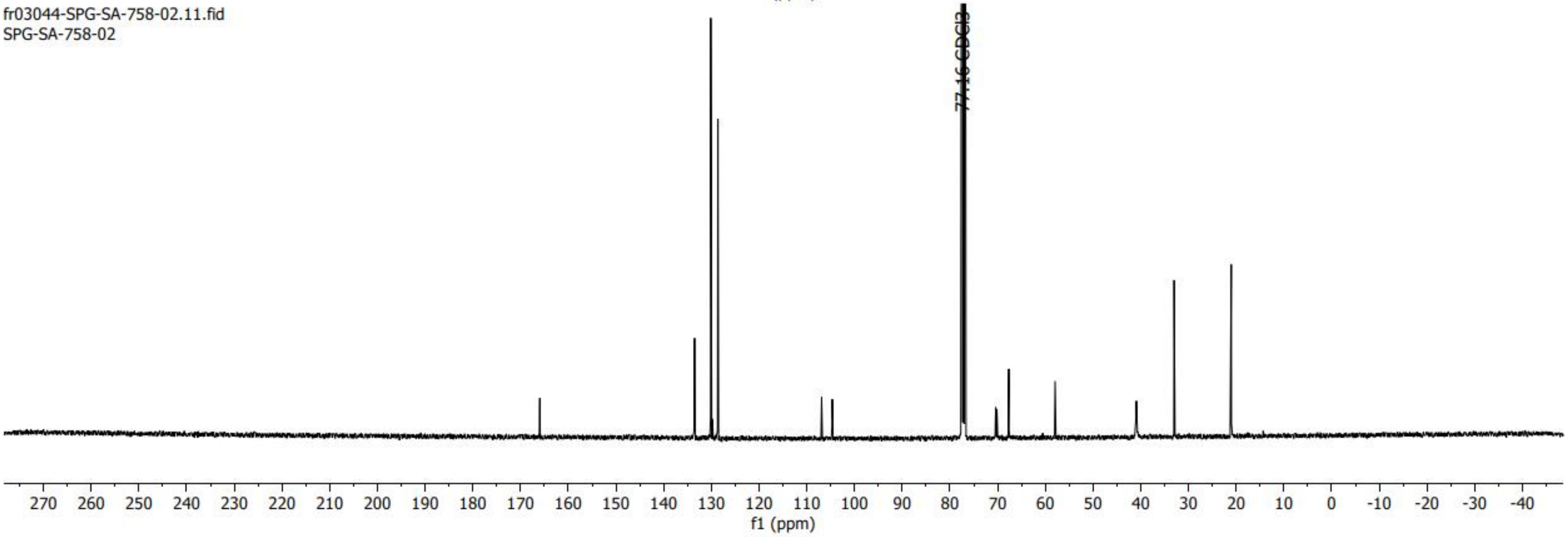

Feb03003-SPG-SA-755-02.12.fid
SPG-SA-755-02

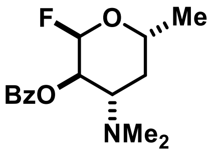

18e in $CDCl_3$
^{19}F (282 MHz)

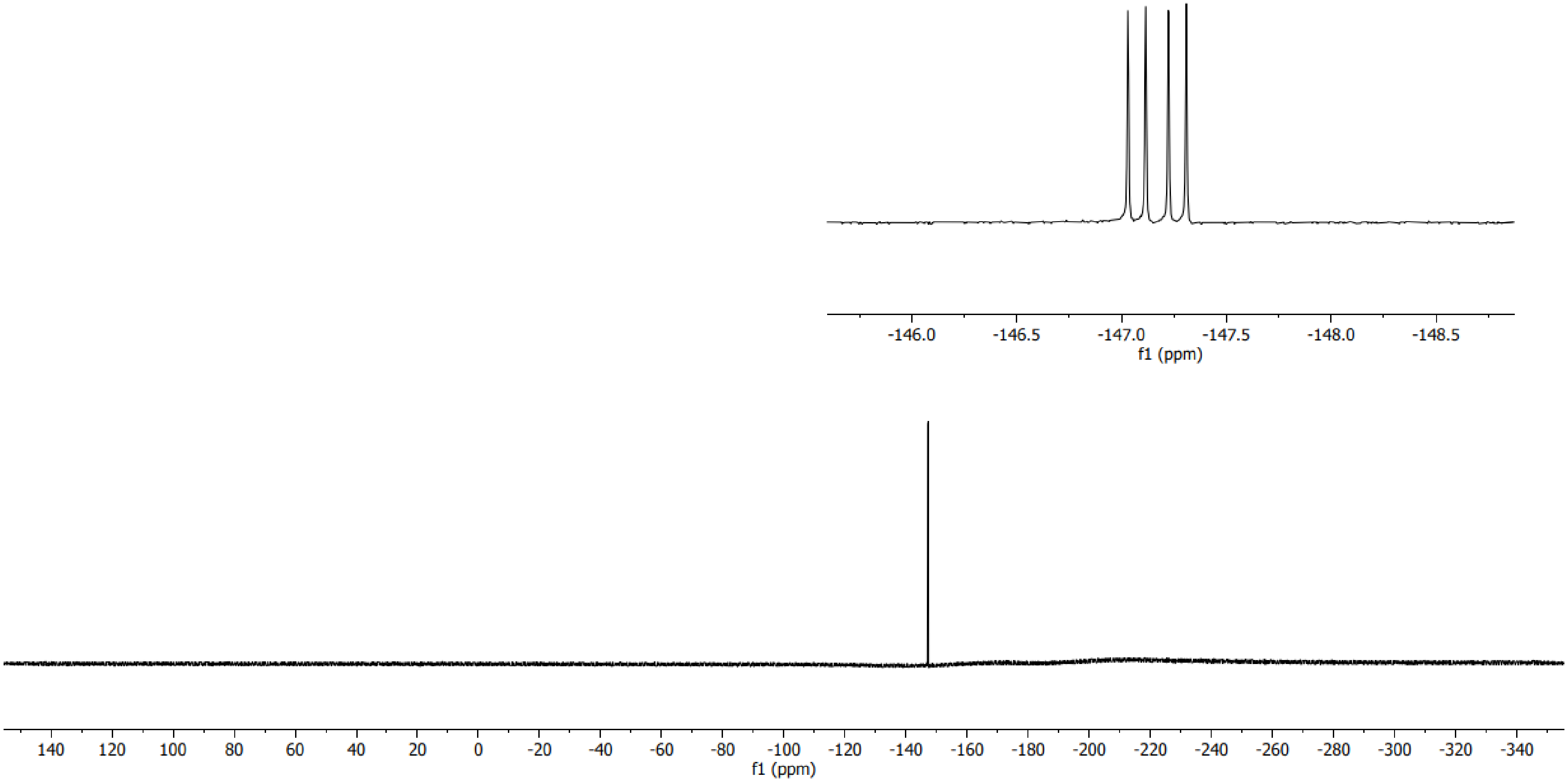

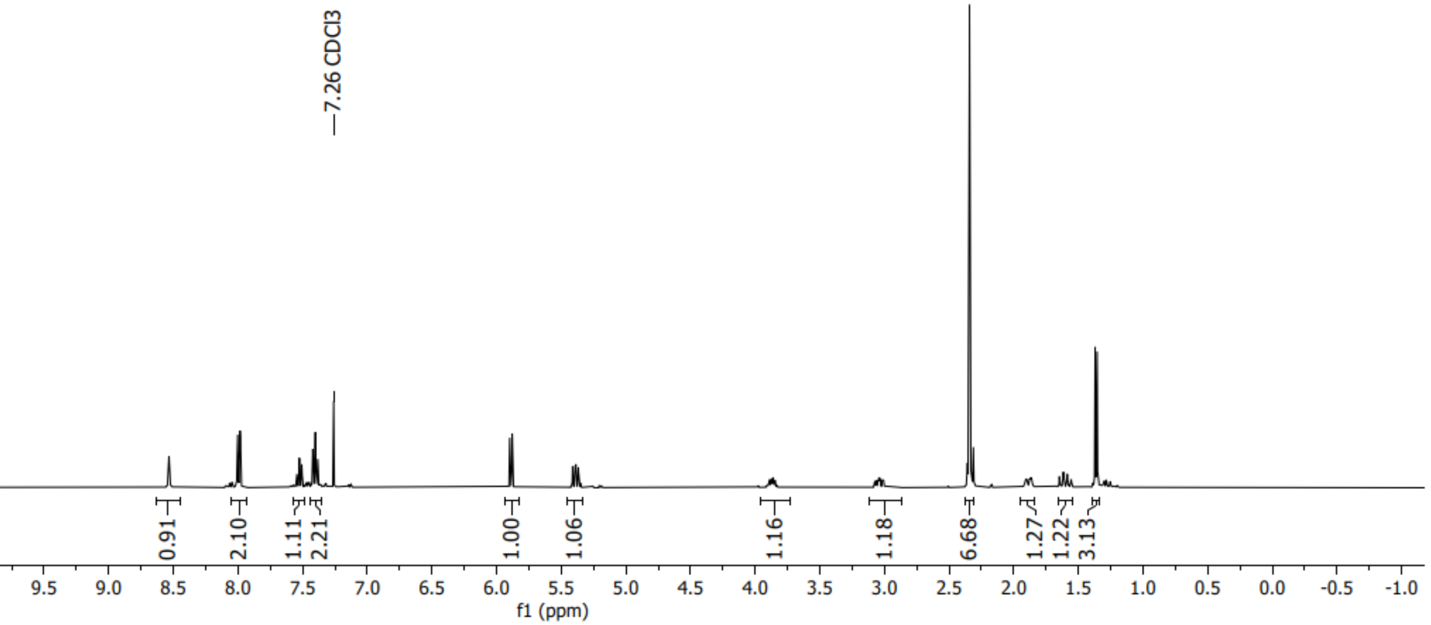

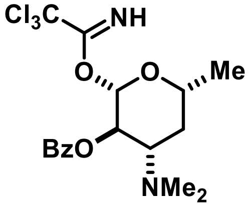

18f in $CDCl_3$

^{1}H (400 MHz)

$^{13}C\{^{1}H\}$ (101 MHz)

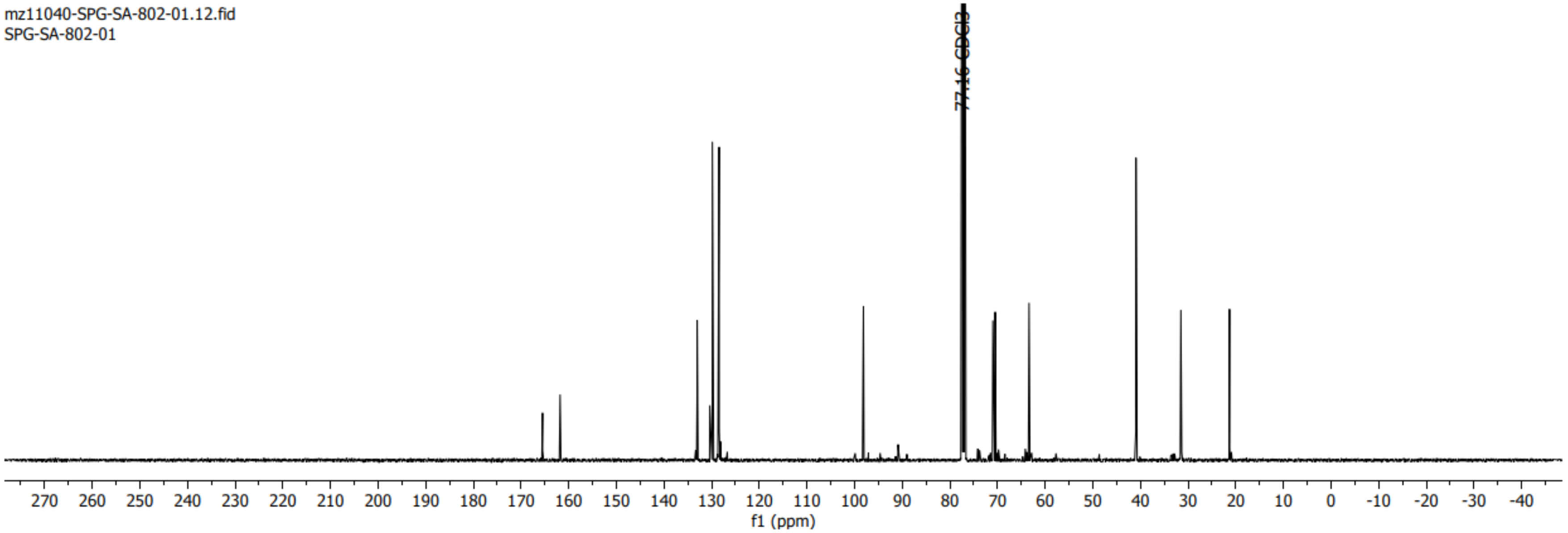

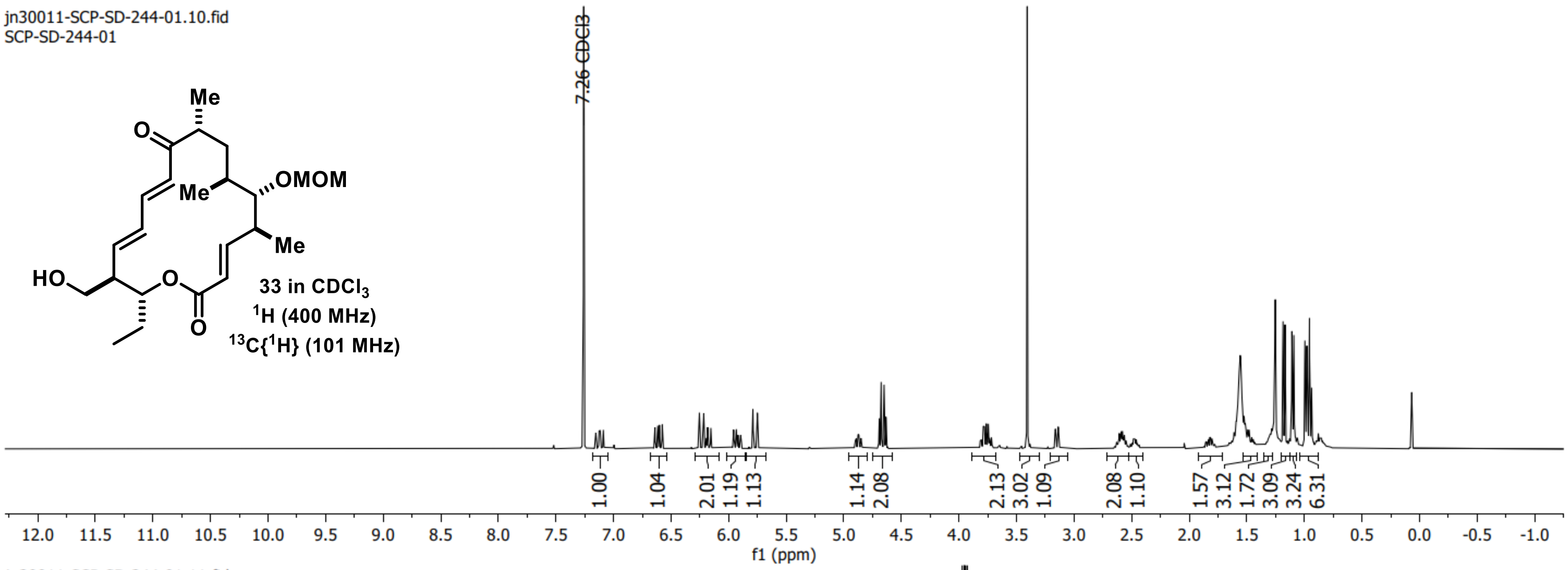
jn30011-SCP-SD-244-01.10.fid
SCP-SD-244-01
Me
O
OMOM
Me
Me
HO
O
O
33 in $CDCl_3$
^1H (400 MHz)
$^{13}C\{^1H\}$ (101 MHz)
7.26 CDCl3
1.00
1.04
2.01
1.19
1.13
1.14
2.08
2.13
3.02
1.09
2.08
1.10
1.57
3.12
1.72
3.09
3.24
6.31
f1 (ppm)

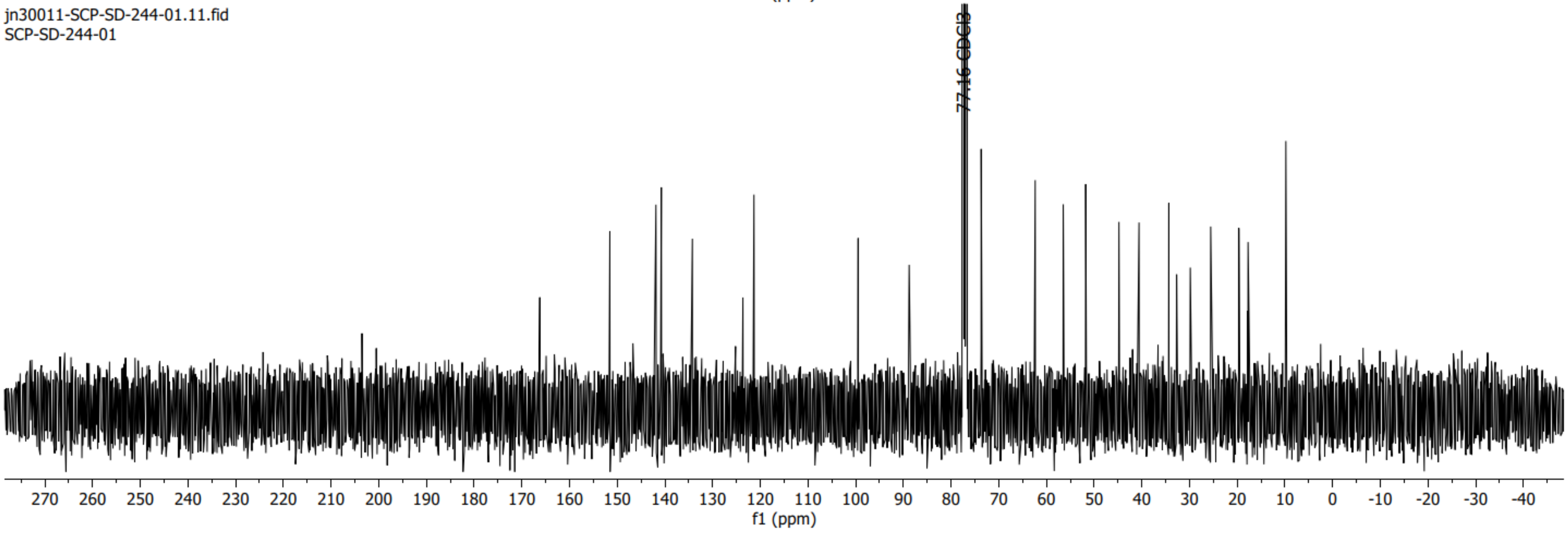
jn30011-SCP-SD-244-01.11.fid
SCP-SD-244-01
77.16 CDCl3
f1 (ppm)

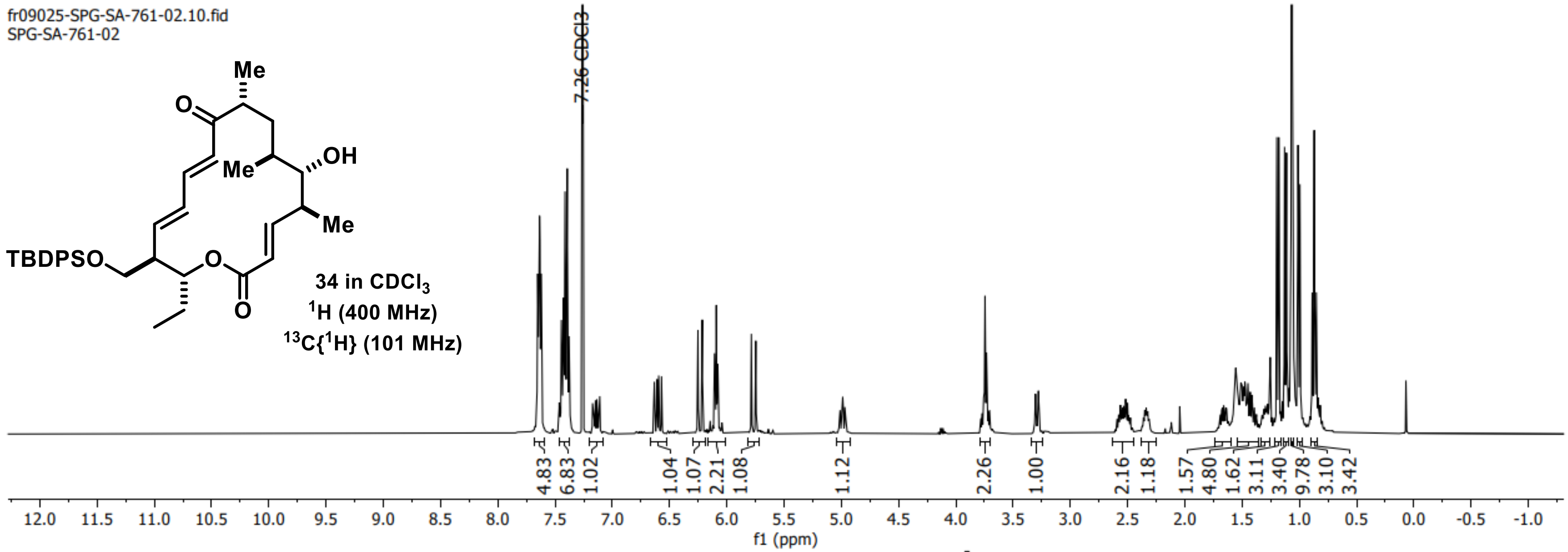

fr09025-SPG-SA-761-02.10.fid
SPG-SA-761-02
Me
O
OH
Me
Me
TBDPSO
O
O
34 in CDCl3
1H (400 MHz)
13C{1H} (101 MHz)
7.26 CDCl3
4.83
6.83
1.02
1.04
1.07
2.21
1.08
1.12
2.26
1.00
2.16
1.18
1.57
4.80
1.62
3.11
3.40
9.78
3.10
3.42
12.0 11.5 11.0 10.5 10.0 9.5 9.0 8.5 8.0 7.5 7.0 6.5 6.0 5.5 5.0 4.5 4.0 3.5 3.0 2.5 2.0 1.5 1.0 0.5 0.0 -0.5 -1.0
f1 (ppm)

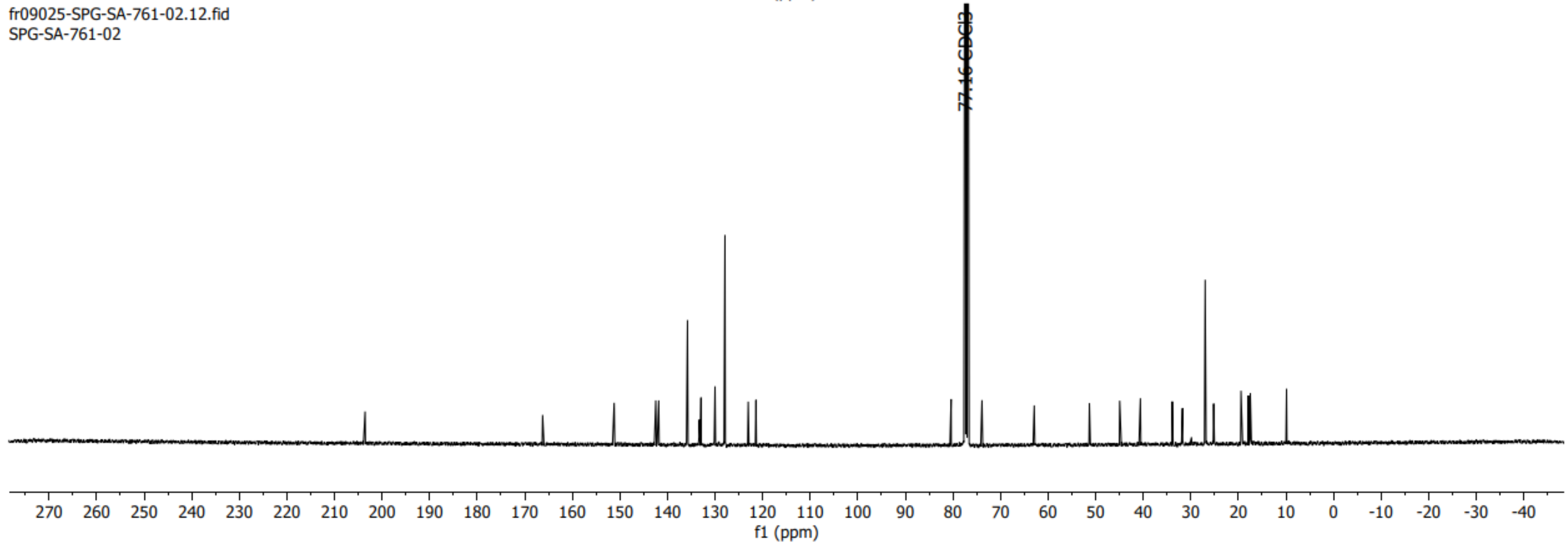

fr09025-SPG-SA-761-02.12.fid
SPG-SA-761-02
77.16 CDCl3
270 260 250 240 230 220 210 200 190 180 170 160 150 140 130 120 110 100 90 80 70 60 50 40 30 20 10 0 -10 -20 -30 -40
f1 (ppm)

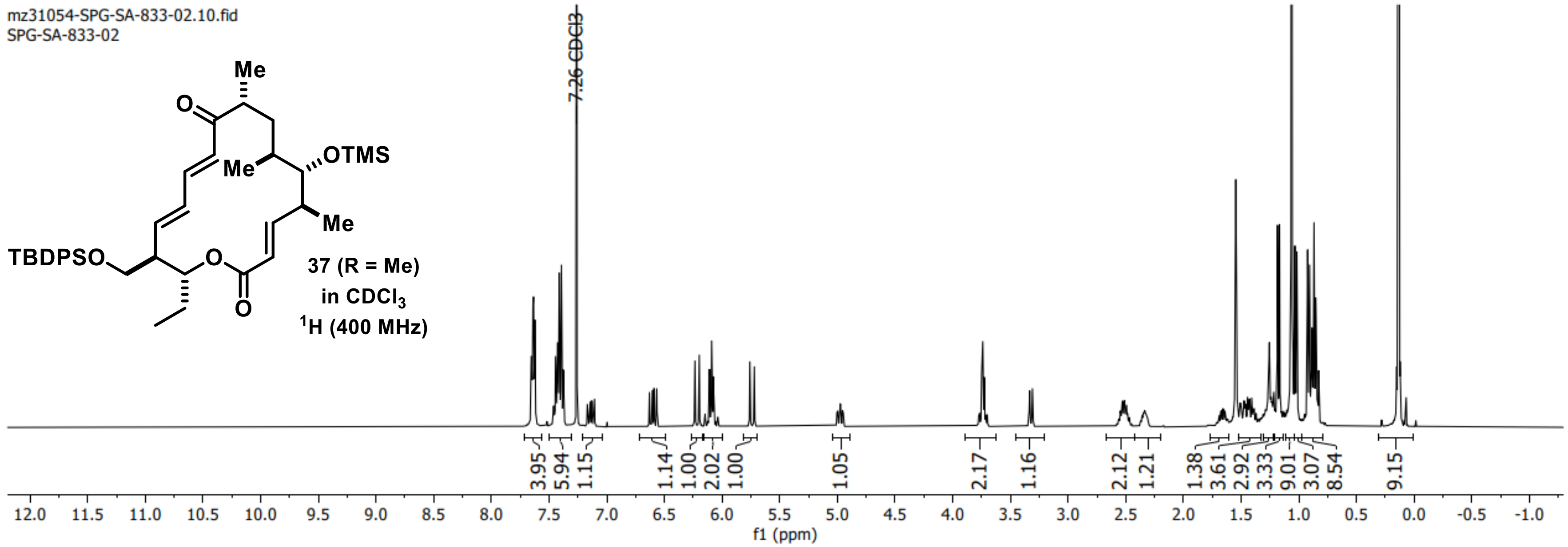
mz31054-SPG-SA-833-02.10.fid
SPG-SA-833-02
Me
O
OTMS
Me
Me
TBDPSO
O
O
37 (R = Me)
in CDCl3
1H (400 MHz)
7.26 CDCl3
3.95
5.94
1.15
1.14
1.00
2.02
1.00
1.05
2.17
1.16
2.12
1.21
1.38
3.61
2.92
3.33
9.01
3.07
8.54
9.15
f1 (ppm)

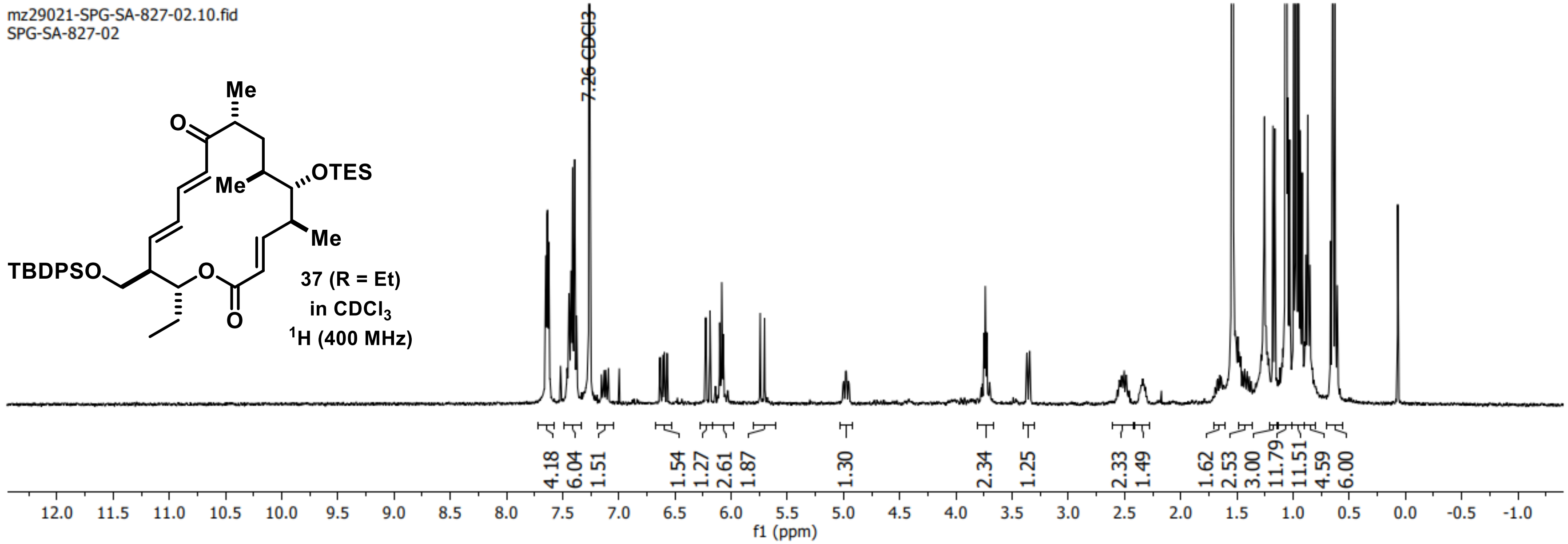
mz29021-SPG-SA-827-02.10.fid
SPG-SA-827-02
Me
O
OTES
Me
Me
TBDPSO
O
O
37 (R = Et)
in CDCl3
1H (400 MHz)
7.26 CDCl3
4.18
6.04
1.51
1.54
1.27
2.61
1.87
1.30
2.34
1.25
2.33
1.49
1.62
2.53
3.00
11.79
11.51
4.59
6.00
f1 (ppm)

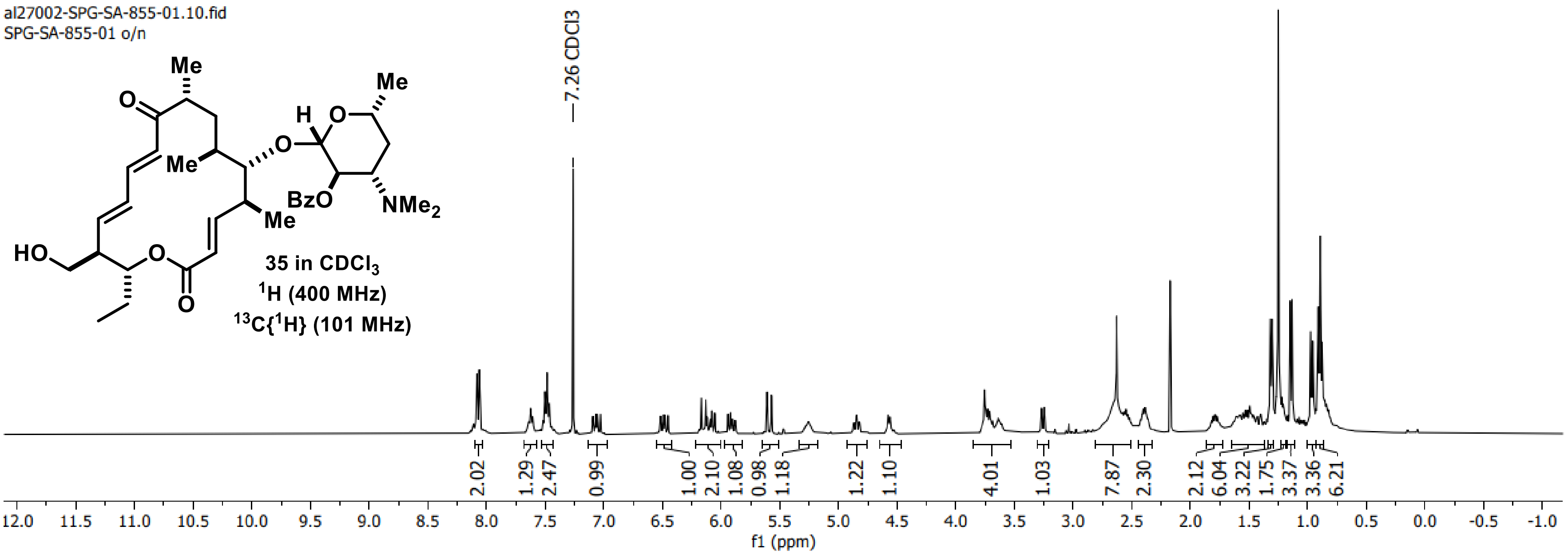
al27002-SPG-SA-855-01.10.fid
SPG-SA-855-01 o/n
Me
O
Me
H
O
O
Me
BzO
NMe2
Me
HO
O
O
35 in CDCl3
1H (400 MHz)
13C{1H} (101 MHz)
7.26 CDCl3
2.02
1.29
2.47
0.99
1.00
2.10
1.08
0.98
1.18
1.22
1.10
4.01
1.03
7.87
2.30
2.12
6.04
3.22
1.75
3.37
3.36
6.21
12.0
11.5
11.0
10.5
10.0
9.5
9.0
8.5
8.0
7.5
7.0
6.5
6.0
5.5
5.0
4.5
4.0
3.5
3.0
2.5
2.0
1.5
1.0
0.5
0.0
-0.5
-1.0
f1 (ppm)

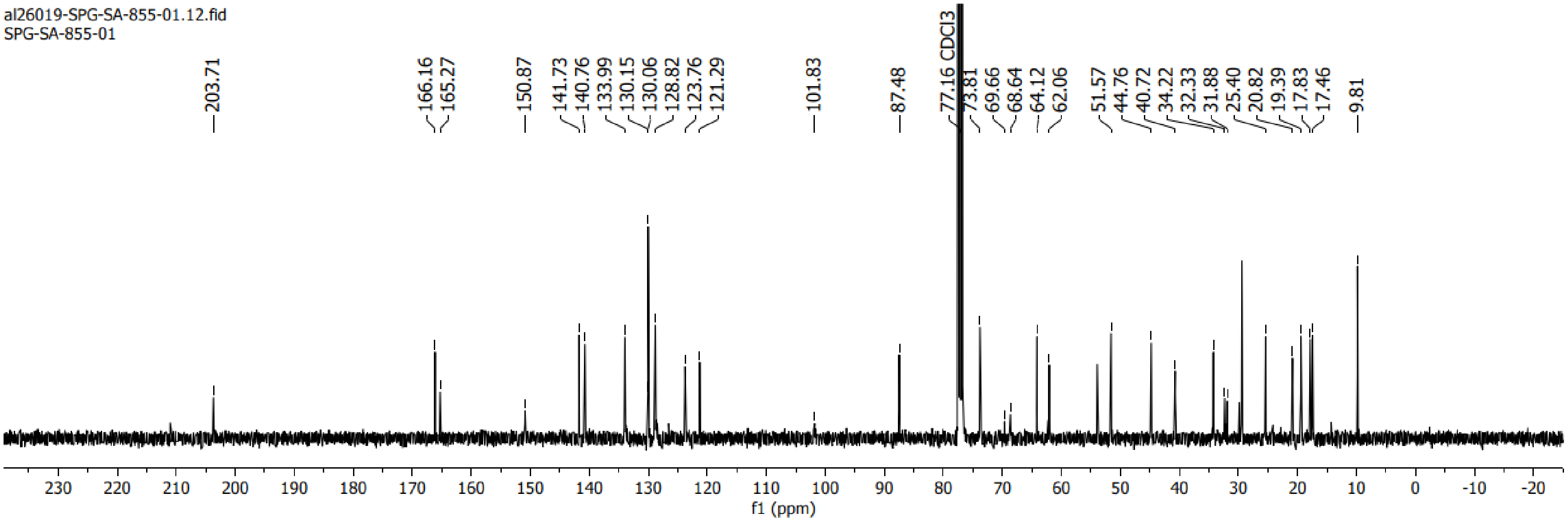
al26019-SPG-SA-855-01.12.fid
SPG-SA-855-01
203.71
166.16
165.27
150.87
141.73
140.76
133.99
130.15
130.06
128.82
123.76
121.29
101.83
87.48
77.16 CDCl3
73.81
69.66
68.64
64.12
62.06
51.57
44.76
40.72
34.22
32.33
31.88
25.40
20.82
19.39
17.83
17.46
9.81
230
220
210
200
190
180
170
160
150
140
130
120
110
100
90
80
70
60
50
40
30
20
10
0
-10
-20
f1 (ppm)

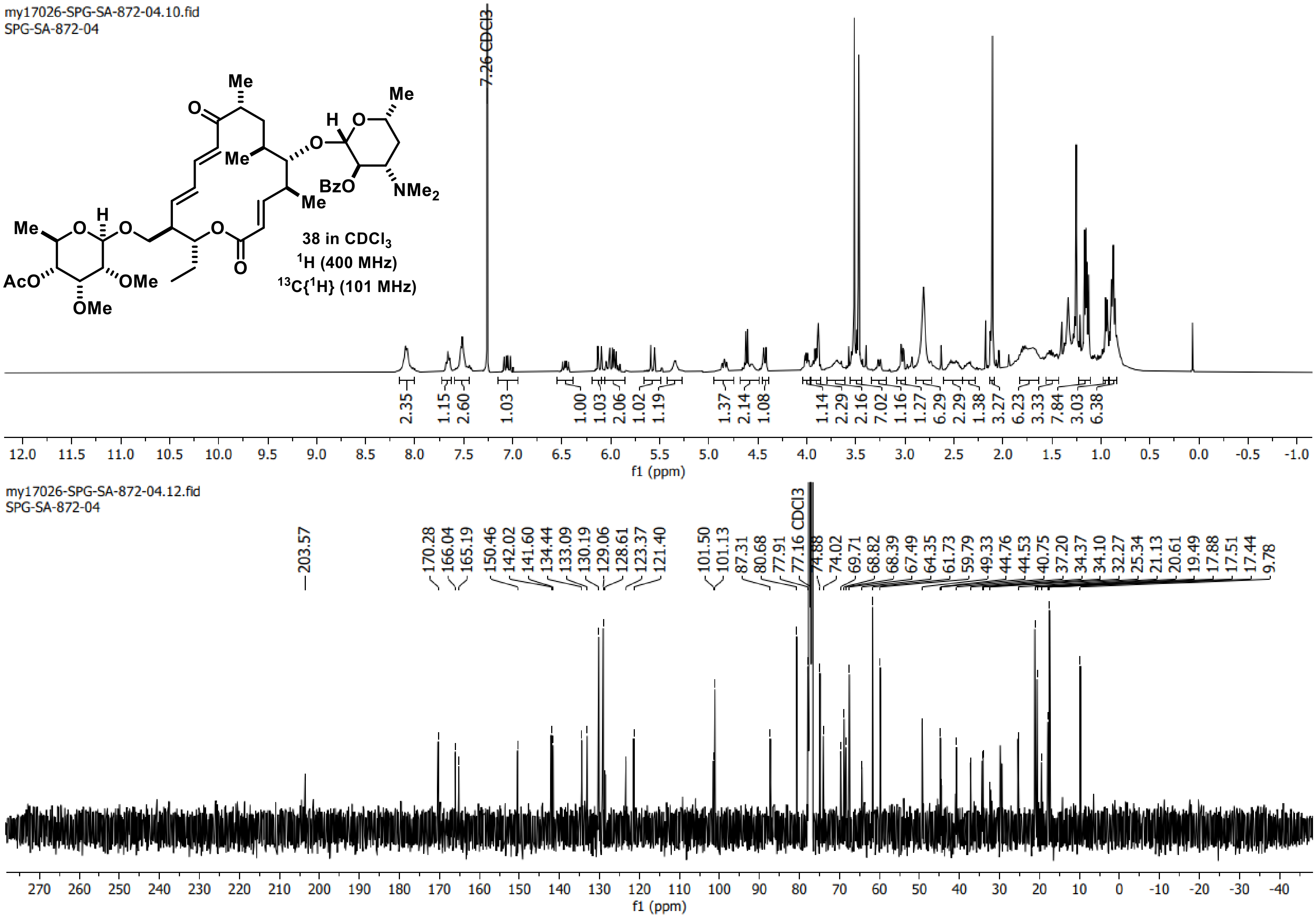
my17026-SPG-SA-872-04.10.fid
SPG-SA-872-04
38 in CDCl3
1H (400 MHz)
13C{1H} (101 MHz)
7.26 CDCl3
2.35 1.15 2.60 1.03 1.00 1.03 2.06 1.02 1.19 1.37 2.14 1.08 1.14 2.29 2.16 7.02 1.16 1.27 6.29 2.29 1.38 3.27 6.23 3.33 7.84 3.03 6.38
f1 (ppm)
my17026-SPG-SA-872-04.12.fid
SPG-SA-872-04
203.57
170.28 166.04 165.19 150.46 142.02 141.60 134.44 133.09 130.19 129.06 128.61 123.37 121.40 101.50 101.13 87.31 80.68 77.91 77.16 CDCl3 74.88 74.02 69.71 68.82 68.39 67.49 64.35 61.73 59.79 49.33 44.76 44.53 40.75 37.20 34.37 34.10 32.27 25.34 21.13 20.61 19.49 17.88 17.51 17.44 9.78
f1 (ppm)

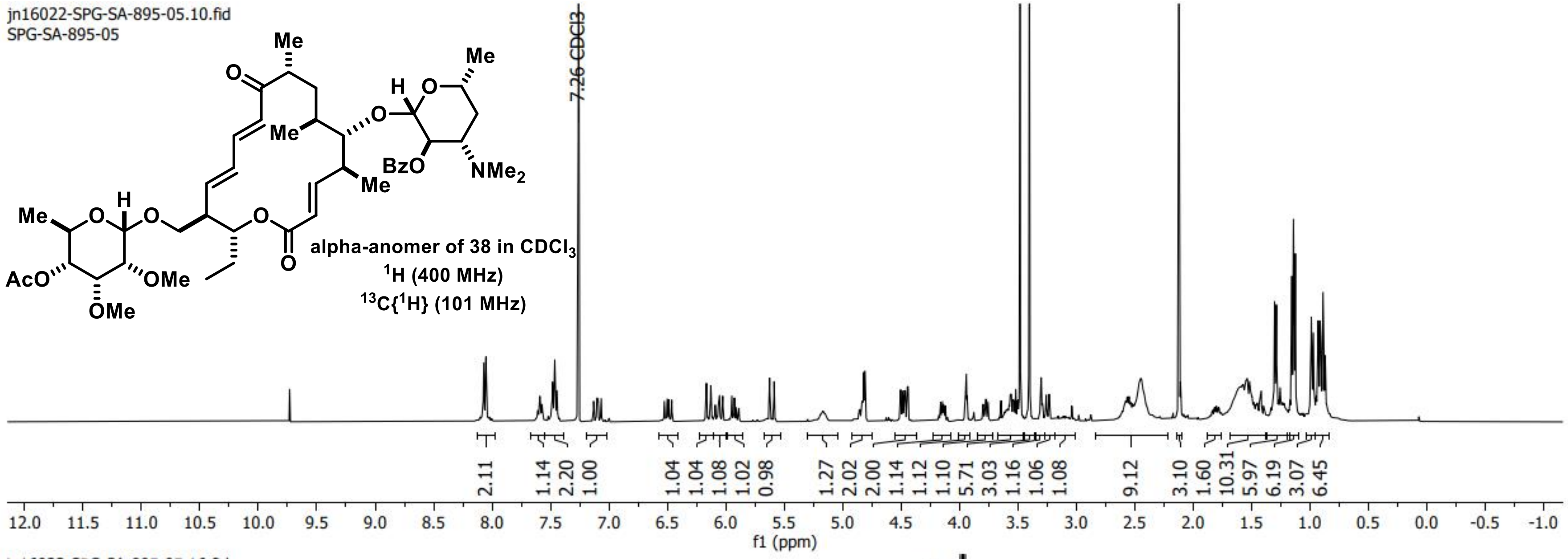
jn16022-SPG-SA-895-05.10.fid
SPG-SA-895-05
alpha-anomer of 38 in CDCl3
1H (400 MHz)
13C{1H} (101 MHz)
7.26 CDCl3
2.11
1.14
2.20
1.00
1.04
1.04
1.08
1.02
0.98
1.27
2.02
2.00
1.14
1.12
1.10
5.71
3.03
1.16
1.06
1.08
9.12
3.10
1.60
10.31
5.97
6.19
3.07
6.45
f1 (ppm)

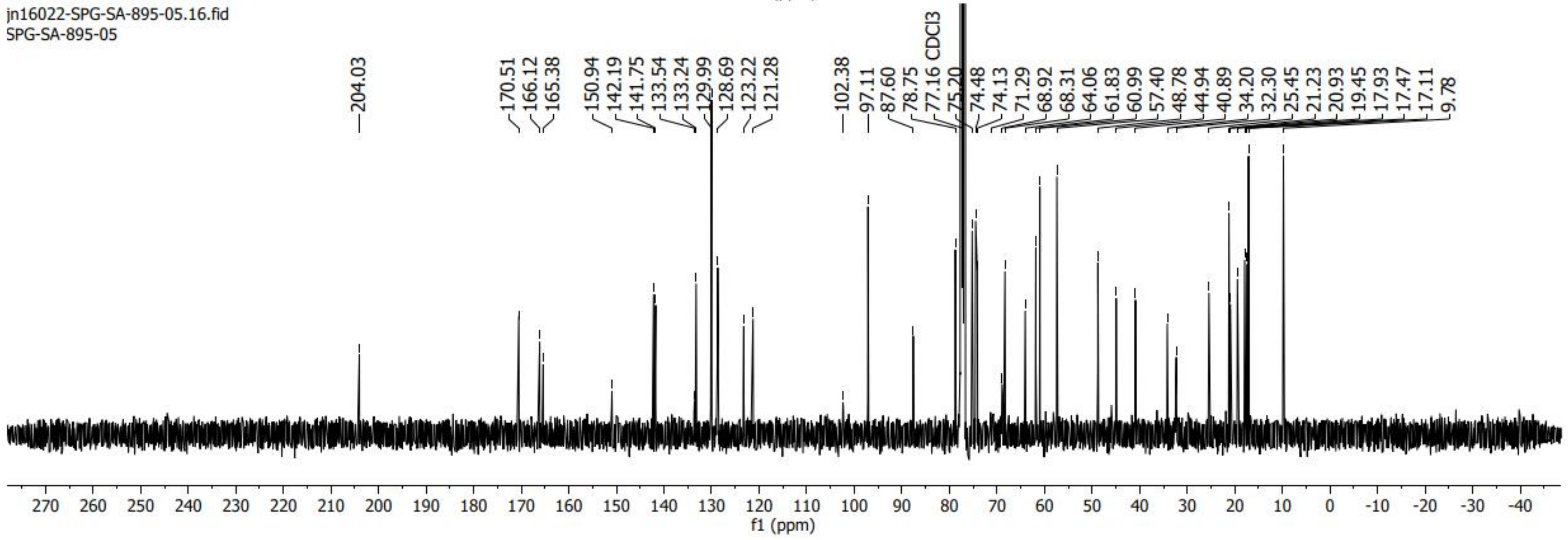
jn16022-SPG-SA-895-05.16.fid
SPG-SA-895-05
204.03
170.51
166.12
165.38
150.94
142.19
141.75
133.54
133.24
129.99
128.69
123.22
121.28
102.38
97.11
87.60
78.75
77.16 CDCl3
75.20
74.48
74.13
71.29
68.92
68.31
64.06
61.83
60.99
57.40
48.78
44.94
40.89
34.20
32.30
25.45
21.23
20.93
19.45
17.93
17.47
17.11
9.78
f1 (ppm)

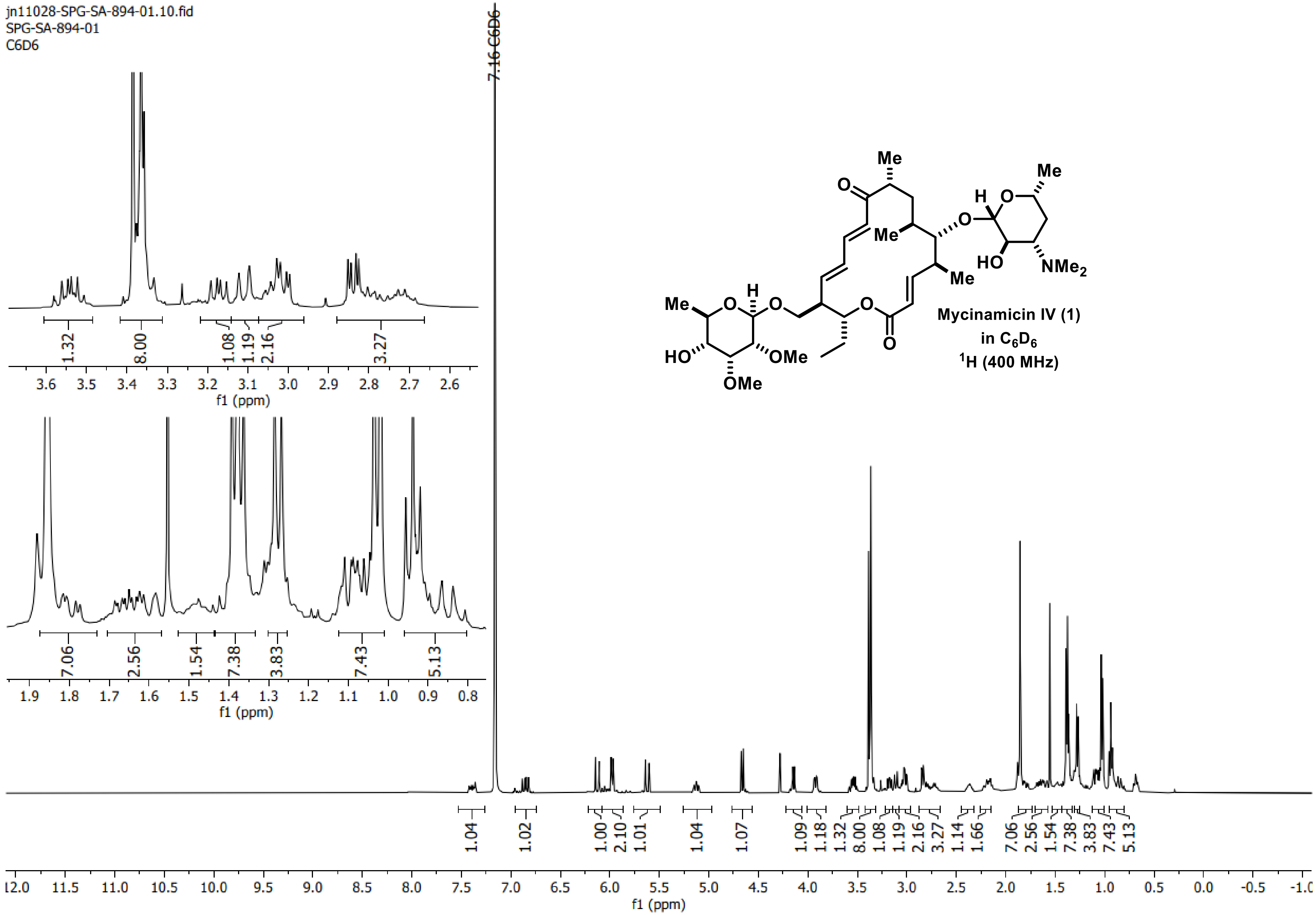
jn11028-SPG-SA-894-01.10.fid
SPG-SA-894-01
C6D6
7.16 C6D6
Mycinamicin IV (1)
in C_6D_6
^{1}H (400 MHz)
Me
O
H
NMe2
HO
OMe
f1 (ppm)

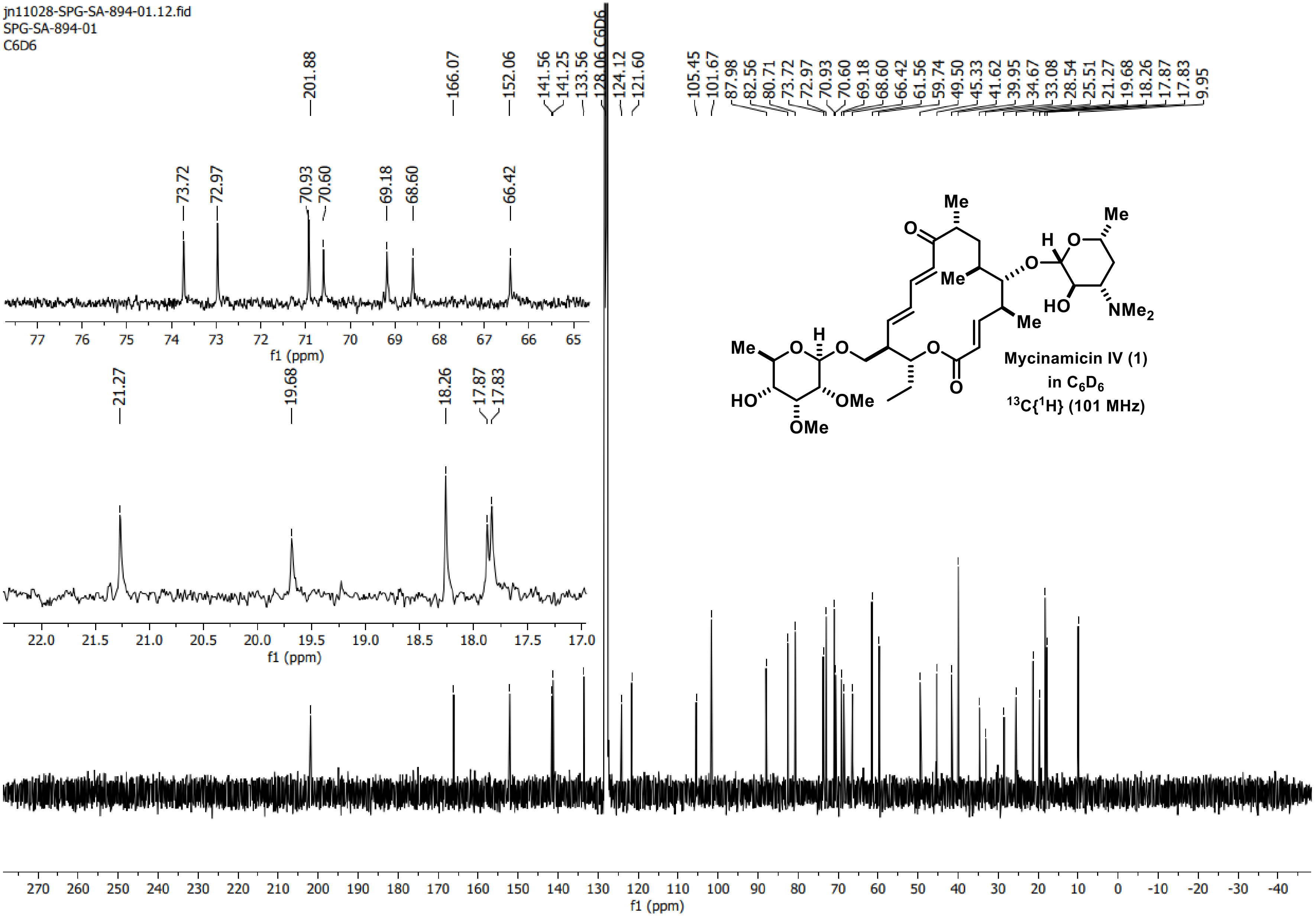
jn11028-SPG-SA-894-01.12.fid
SPG-SA-894-01
C6D6
201.88
166.07
152.06
141.56
141.25
133.56
128.06 C6D6
124.12
121.60
105.45
101.67
87.98
82.56
80.71
73.72
72.97
70.93
70.60
69.18
68.60
66.42
61.56
59.74
49.50
45.33
41.62
39.95
34.67
33.08
28.54
25.51
21.27
19.68
18.26
17.87
17.83
9.95
f1 (ppm)
Mycinamicin IV (1)
in C_6D_6
$^{13}C\{^1H\}$ (101 MHz)

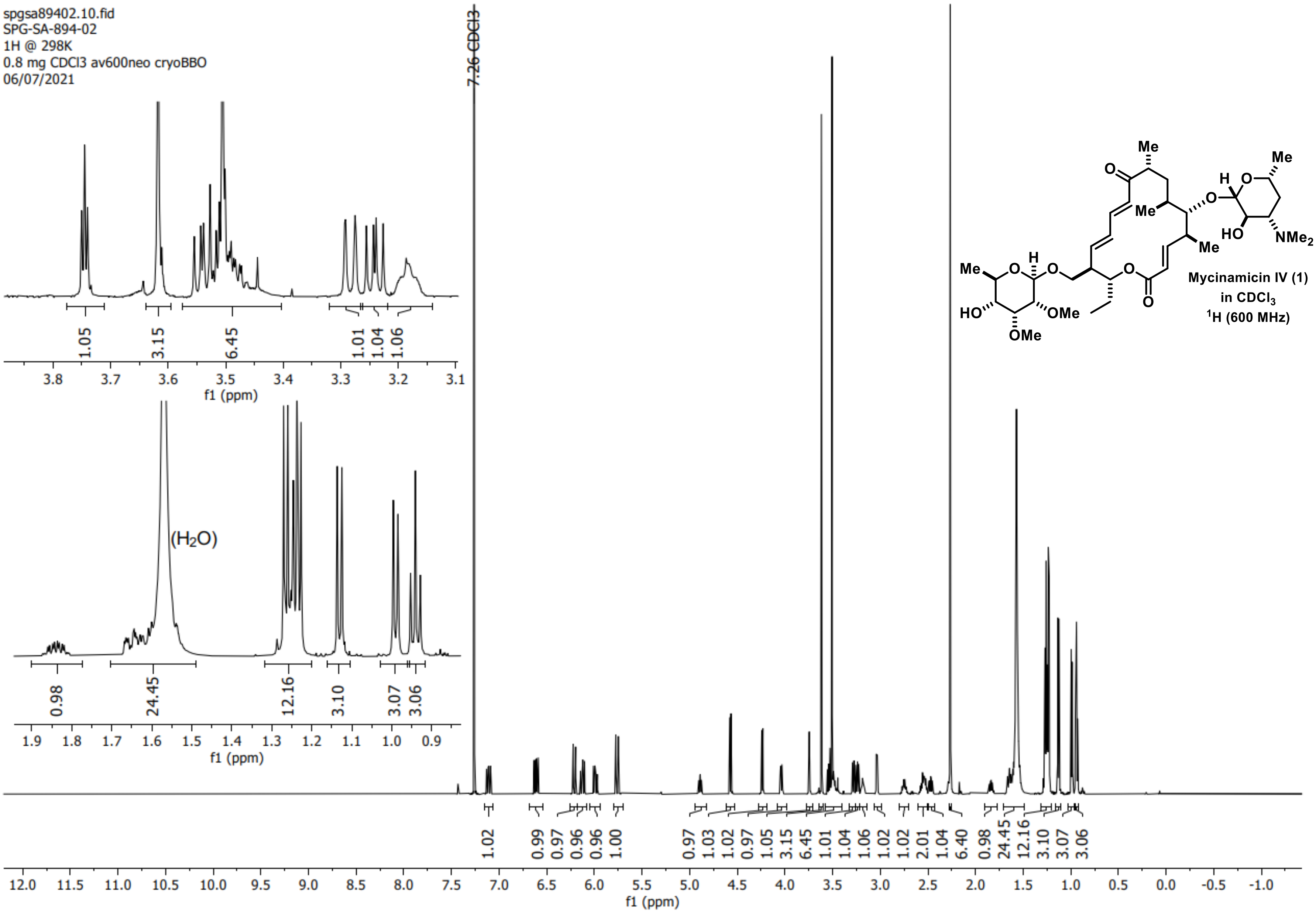
spgsa89402.10.fid
SPG-SA-894-02
1H @ 298K
0.8 mg CDCl3 av600neo cryoBBO
06/07/2021
7.26 CDCl3
(H2O)
Mycinamicin IV (1)
in CDCl3
1H (600 MHz)
f1 (ppm)

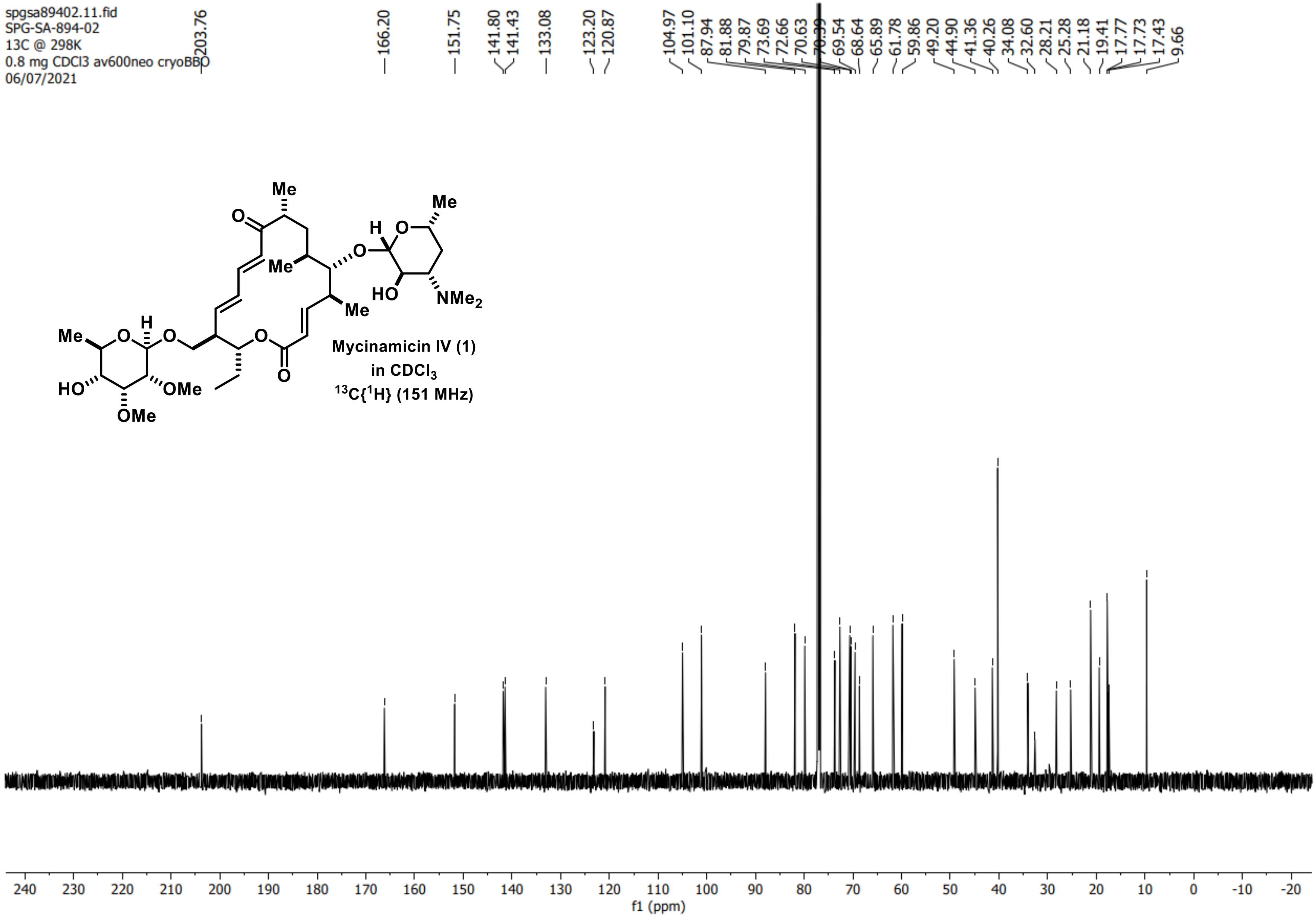
spgsa89402.11.fid
SPG-SA-894-02
13C @ 298K
0.8 mg CDCl3 av600neo cryoBBO
06/07/2021
203.76
166.20
151.75
141.80
141.43
133.08
123.20
120.87
104.97
101.10
87.94
81.88
79.87
73.69
72.66
70.63
69.54
68.64
65.89
61.78
59.86
49.20
44.90
41.36
40.26
34.08
32.60
28.21
25.28
21.18
19.41
17.77
17.73
17.43
9.66
Me
O
H
O
Me
Me
O
HO
NMe2
Me
Me
O
H
O
O
HO
OMe
OMe
O
O
Mycinamicin IV (1)
in CDCl3
13C{1H} (151 MHz)
240 230 220 210 200 190 180 170 160 150 140 130 120 110 100 90 80 70 60 50 40 30 20 10 0 -10 -20
f1 (ppm)

References

[1] H. Nöth, H. Vahrenkamp, *J. Organomet. Chem.* **1968**, *11*, 399-405.

[2] G. Späth, A. Fürstner, *Angew. Chem. Int. Ed.* **2021**, *60*, 7900-7905.

[3] B. Herlé, G. Späth, L. Schreyer, A. Fürstner, *Angew. Chem. Int. Ed.* **2021**, *60*, 7893-7899.

[4] T. Matsumoto, H. Maeta, K. Suzuki, G. Tsuchihashi, *Tetrahedron Lett.* **1988**, *29*, 3575-3578.
